# The malaria parasite *Plasmodium falciparum* Vps15 is a Phosphatidylinositol 3-kinase interactor important for host-cell cytosol delivery to the food vacuole and apicoplast and mitochondrion dynamics

**DOI:** 10.1371/journal.ppat.1014526

**Published:** 2026-08-18

**Authors:** Alexandra Bourgeois, Louis-David Martin, Harpreet Kaur, Dominic Gagnon, Joel B. Dacks, Dave Richard

**Affiliations:** 1 Department of Microbiology-Infectious Diseases and Immunology, Faculty of Medicine, Université Laval, Quebec City, Quebec, Canada; 2 CHU de Québec-Université Laval Research Centre, Québec, Québec, Canada; 3 Centre de Recherche en Infectiologie de l’Université Laval, Québec, Québec, Canada; 4 Division of Infectious Diseases, Department of Medicine, and Department of Biological Sciences, University of Alberta, Edmonton, Canada; 5 Centre for Life’s Origins and Evolution, Department of Genetics, Evolution and Environment, University College London, London, United Kingdom; 6 Institute of Parasitology, Biology Centre, Czech Academy of Sciences, České Budějovice (Budweis), Czech Republic; University of Geneva Faculty of Medicine: Universite de Geneve Faculte de Medecine, SWITZERLAND

## Abstract

The malaria parasite *Plasmodium falciparum* is an obligate intracellular organism that spends an important part of its lifecycle inside human erythrocytes. The endocytosis of host-cell cytosol and its delivery to a lysosome-like organelle called the food vacuole are critical for the parasite’s survival and proliferation. Recent work has started to identify some of the molecular players involved in this pathway, but much remains to be discovered. Evidence suggests that phosphatidylinositol-3-phosphate (PI3P) plays a central role in this process. In unicellular eukaryotes, such as yeast, PI3P is generated by a single PI3-kinase, whose activity is regulated by a pseudokinase called Vps15. *P. falciparum* also possesses a PI3K that generates PI3P and bioinformatics analysis has revealed the presence of an uncharacterized putative orthologue of Vps15. We here present our characterization of PfVps15. We first show that it is constitutively expressed throughout the asexual erythrocytic cycle and that it interacts with PfPI3K, but unlike in yeast and mammalian cells, it is potentially not part of a heterotetrameric complex. The removal of PfVps15 from its site of action by knock sideways led to rapid parasite death. Phenotypic analyses revealed a decrease in PI3P levels, the abrogation of the delivery of host-cell cytosol containing vesicles to the food vacuole, and defects in apicoplast biogenesis and mitochondrial fission. Collectively, our data has identified a protein critical for the synthesis of PI3P and provides molecular evidence for the importance of this lipid in the vesicular trafficking pathway of host-cell cytosol, and apicoplast and mitochondrion dynamics.

## Author summary

The malaria parasite causes one of the most devastating infectious diseases in the world. In humans, the parasite invades and then develops inside red blood cells. To cover some of its nutritional needs and generate space for its growth, the parasite needs to transport hemoglobin from the host red blood cell into a structure that is called the food vacuole, where it will be digested. This process is essential for the survival of the parasite so it represents an attractive target for the development of drugs against malaria. The work that we present here describes a protein named PfVps15 that is critical for the parasite to deliver hemoglobin to the food vacuole, for the generation of an organelle named the apicoplast and the separation of the mitochondrion. In the absence of PfVps15, the parasite cannot survive. It might thus potentially represent a new antimalarial target.

## Introduction

The *Plasmodium falciparum* parasite causes the most severe form of malaria, a disease that continues to have a devastating impact on humanity, with over 282 million cases and 610,000 deaths reported in 2024 [1]. While significant progress in reducing malaria cases and deaths has been made over the past two decades, this decline has unfortunately stagnated in recent years [1]. The spread of resistance to the first-line treatment, artemisinin (ART), in Southeast Asia [2–4] and reports of partial ART resistance in Africa [5–7] highlight the urgent need to identify new therapeutic targets.

Phosphoinositides (PIPs) are minor lipids that play essential roles in various cellular processes in eukaryotic cells such as the cell cycle, autophagy and vesicular trafficking [8,9]. PIPs are derived from the phosphorylation of phosphatidylinositol at positions 3, 4, and 5, generating seven distinct PIP species through the action of lipid kinases and phosphatases. These species localize to specific organelles and membrane microdomains in order to facilitate the recruitment of proteins containing specialized domains with varying affinities for different PIP species [10]. Several studies have highlighted the critical roles of PIPs in the biology of the malaria parasite [11,12], such as in merozoite formation [13], red blood cell invasion [14–16] and in apicoplast dynamics [17,18].

A crucial phase of the parasite’s lifecycle in the human host occurs within red blood cells. The parasite relies on extensive endocytosis of host cell cytosol (HCC), composed in majority of hemoglobin (Hb), for its needs in amino acids [19,20] and also to create space for its development [21,22]. The endocytosis of HCC occurs at structures named cytostomes, invaginations of the parasite plasma membrane and the parasitophorous vacuole membrane [23,24]. Recent work has revealed that several proteins localized at the cytostomal collar were critical for endocytosis, one of which is Kelch13 (K13), a kelch domain-containing protein and a well-established marker of ART resistance [25–27]. Until recently, in opposition to other eukaryotes, it was thought that clathrin did not seem to be involved in the process [25,28]. However, recent work has shown that conditionally knocking down the clathrin heavy chain leads to elongated cytostomes and impaired hemoglobin digestion [29]. The molecular effectors underlying the trafficking of HCC to the food vacuole (FV), a lysosome-like organelle where the digestion of Hb by parasite proteases occurs, were unknown until the recent demonstration that the *P. falciparum* homologue of Vps45, a conserved protein involved in endolysosomal transport in other eukaryotes, was essential for this process [30].

Evidence supports the importance of PI3P in the vesicular trafficking of HCC to the FV [31–34]. Indeed, PI3P is found at the FV membrane [18,35] and incubation of parasites with PI3K inhibitors [32] or the conditional inactivation of *PfPI3K* [34] result in the accumulation of vesicles filled with Hb. In addition, the conditional inactivation of PfVps45, PfRab5b and PfRbsn5L also leads to an increase in HCC-filled vesicles, some of which are labelled with PI3P [30], [36]. Furthermore, the PI3P binding protein PfPX1 [31] has also been revealed to be important for the delivery of HCC vesicles to the FV.

The apicoplast is a plastid-like organelle that arose from a secondary endosymbiosis event that is found in apicomplexan parasites like *P. falciparum* and *Toxoplasma gondii* [37,38]. In the malaria parasite, this organelle is essential for the generation of isoprenoid precursors, iron-sulfur cluster cofactors and Coenzyme A (reviewed in [39]). Conditional abrogation of PfPI3K or TgPI3K leads to defects in the biogenesis of the apicoplast, suggesting that PI3P is critical in this process in both parasite species [34,40].

In mammalian cells, PI3P is generated by class II and class III PI3Ks, however unicellular eukaryotes, such as yeast and *P. falciparum,* only possess a single class III PI3K (named Vps34 in yeast). In model cells, this PI3K is part of two different heterotetrameric complexes referred to as complex 1 and 2 [41–43]. In addition to class III PI3K, both complexes share the regulatory scaffold protein Vps15, and Atg6 (named Beclin 1 in mammalian cells). Complex 1 additionally contains Autophagy-related 14 (Atg14) and plays roles in autophagy [44–46] whilst Atg14 is replaced by Vps38 (UV resistance associated (UVRAG) in mammalian cells) in complex 2, which is implicated in endocytic sorting, autophagy and cytokinesis [47–49].

Vps15 is a conserved pseudokinase that is critical for the regulation of class III PI3K activity [48,50–52]. Vps15 orthologues are present in apicomplexan parasites [53,54] and in the model apicomplexan *T. gondii*, TgVps15 is required for the biogenesis of the apicoplast and autophagy [55]. The role of the putative *P. falciparum* Vps15 orthologue is currently unknown.

We here present our characterization of the *P. falciparum* homologue of Vps15. We first show by immunoprecipitation that it forms a complex with PfPI3K, but intriguingly no obvious additional components were consistently identified. The potential absence of the heterotetrameric complexes found in other eukaryotes suggests *Plasmodium*-specific adaptations. We next demonstrate that PfVps15 is constitutively expressed throughout the asexual erythrocytic stages and that it primarily localizes to structures labelled with endosomal markers and additionally to the FV, to cytosolic foci found near branching apicoplasts and the mitochondrion. Finally, conditional inactivation by knock sideways revealed that PfVps15 is critical for the generation of PI3P, the delivery of HCC to the FV, apicoplast dynamics and mitochondrial fission.

## Results and discussion

### PfVps15 interacts with PfPI3K

In order to characterize the role of PfVps15, we used the selection-linked integration (SLI) strategy to endogenously tag the C terminus of its gene with GFP following a single cross-over recombination event [56], as represented in S1A Fig. In addition to the GFP tag, a double FK506 binding protein (FKBP) domain was also inserted to allow the functional characterization of PfVps15 by knock sideways (KS) [56]. Polymerase chain reaction (PCR) was used to confirm the insertion of the plasmid at the correct genomic locus and to verify the absence of the WT allele, as demonstrated in S1B Fig. One clonal line containing the 2xFKBP-GFP tagged PfVps15 was then used to follow the expression of the protein throughout the *P. falciparum* asexual cycle. To do this, parasite protein extracts were taken at different times during the asexual cycle and analyzed by Western Blot using an anti-GFP antibody. This revealed a single band at each time point corresponding to a slightly larger than the expected size of the 201.3 kDa PfVps15-2xFKBP-GFP fusion protein (S1C Fig). An antibody against Aldolase was used as a constitutively expressed control.

**Fig 1:**
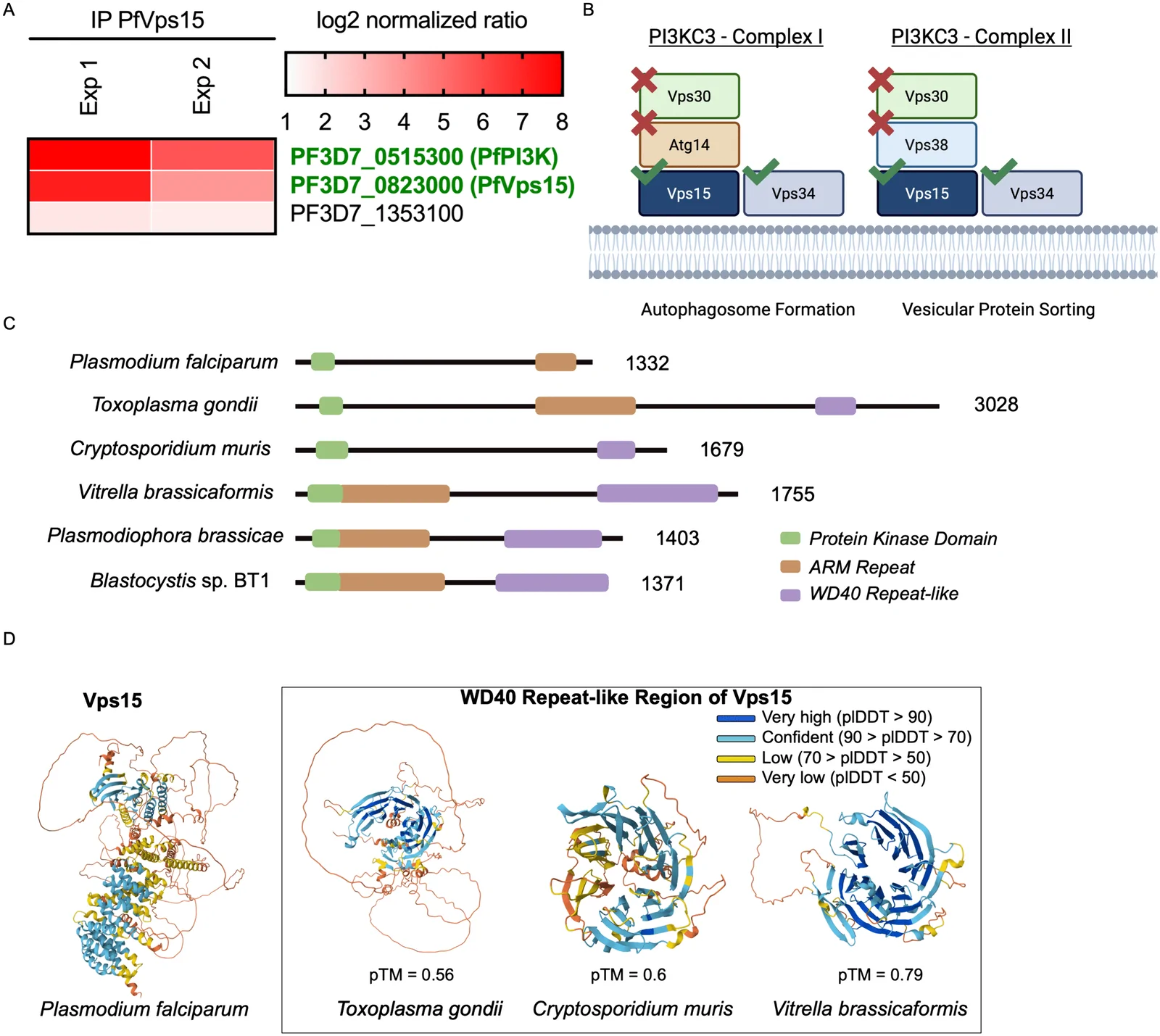
PfVps15 interacts with PfPI3K in *Plasmodium falciparum* asexual blood stages. **A.** Heat map indicates proteins enriched in two biological replicates in the PfVps15 immunoprecipitation along with their PlasmoDB identifiers. **B.** Illustration of the two PI3KC3 complexes present in eukaryotic cells along with the primary function of each complex. Green checkmarks indicate complex proteins with an identified homologue present in *P. falciparum*. Red X indicate complex proteins absent with no identifiable homologues in *P. falciparum*. Created in BioRender. Bourgeois, A. 2025. https://biorender.com/ld2tq8c. **C.** Schematic of Vps15 with putative predicted domains in the selected taxa across SAR lineages showing the absence of WD40 repeat-like domain in *P. falciparum*. Numbers represent length of the proteins in amino acids. D. Comparison of the predicted PfVps15 protein structure (Uniprot id: Q8IB64, pLDDT: 49.53) with the WD40 repeat-like region identified in Vps15 homologs from representative SAR species (in black box) confirms the absence of WD40 repeat-like region in *P. falciparum*. pTM and pLDDT values are provided.

We next wanted to determine whether PfVps15 was part of a protein complex, so we attempted to transfect the PfVps15-2xFKBP-GFP line with a plasmid expressing the biotin ligase BirA fused to FRB to employ dimerization induced BioID from proximity-dependent biotinylation [25]. However, we were not able to recover transfectants. We therefore performed immunoprecipitations on mixed-stage protein extracts with anti-GFP agarose beads followed by mass spectrometry, using an untagged 3D7 parasite line as a negative control. Anti-GFP Western blot showed that PfVps15-2xFKBP-GFP was nicely precipitated with the anti-GFP agarose beads whilst no signal was seen in the 3D7 control (S2 Fig). Several hundred proteins were identified in two biological replicates, but only two were highly enriched in both: PfVps15 and PfPI3K (Fig 1A and S1 Table). Another protein, PF3D7_1353100, a dispensable exported protein [57,58], was only slightly enriched (3 peptides in the PfVps15 vs 1 in the 3D7 control). That only PfVps15 and PfPI3K were identified in the complex was surprising as in several types of eukaryotic cells, Vps15 and PI3K form two different heterotetrameric complexes (complex 1 with Atg6 (named Vps30/Beclin in humans) and Atg14, complex 2 with Atg6 and Vps38). Whilst *Plasmodium* parasites do not possess orthologues of Atg6, Atg14 and Vps38 (Fig 1B, S2 Table and [59]), we expected to identify other potential interactors considering that these proteins are essential for the diverse roles of PI3K in mammalian cells and yeast [41–43,47,53,60–62]. Indeed, the difference in a single subunit between the two complexes dictates their different subcellular localizations. In *P. falciparum*, PI3P is found at several different locations such as the apicoplast membrane, the FV membrane and HCC-containing vesicles which leads to the question as to how the different PI3P pools are differentially generated [17,18,35,36,63]. One possibility could be that putative accessory proteins would only be transiently associated with the core PfPI3K-PfVps15 complex or that their interaction may be too weak to be captured by immunoprecipitation. Interestingly, the C-terminal WD40 domain of human Vps15 is required for its interaction with Rab5 on early endosomes [64,65] and potentially with Beclin 1, the human orthologue of ScAtg6 [42]. However, a previous analysis suggested that this domain was absent in PfVps15 [55]. We thus performed a comparative molecular evolutionary analysis of Vps15 in *P. falciparum*, and across representative organisms from the apicomplexan lineage, as well as the larger alveolate and SAR lineages (S2 and S3 Tables). Homology searching, domain analysis and modelling with AlphaFold3 (Fig 1C and 1D) confirmed that although the WD40 domain is present in Vps15 proteins from other organisms in the SAR clade, including in *T. gondii*, this domain is absent in PfVps15. Vps15 in yeast and mammalian cells is myristoylated [48,50] and recent work has revealed that this was required for the association of the Vps15-Vps34 complex to membranes [51]. PfVps15 possesses an N-terminal glycine and a study on myristoylated proteins in *P. falciparum* suggested that PfVps15 is indeed myristoylated [66]. It is worth mentioning that, contrarily to *P. falciparum, T. gondii* possesses an Atg6 orthologue (S2 Table and [59]), but it is currently unknown whether TgPI3K, TgVps15 and TgAtg6 form a complex. Of note, using yeast two-hybrid, it was shown that the kinetoplastid parasite *Trypanosoma cruzi* Vps15 and Vps34 orthologues were directly interacting [67]. Taken together, our results highlight that the core complex of Vps15 and PI3K is conserved in *P. falciparum* but that other accessory proteins are not easily identified by immunoprecipitation suggestive of potential *Plasmodium*-specific adaptations.

### Determination of the subcellular localization of PfVps15

**Fig 2:**
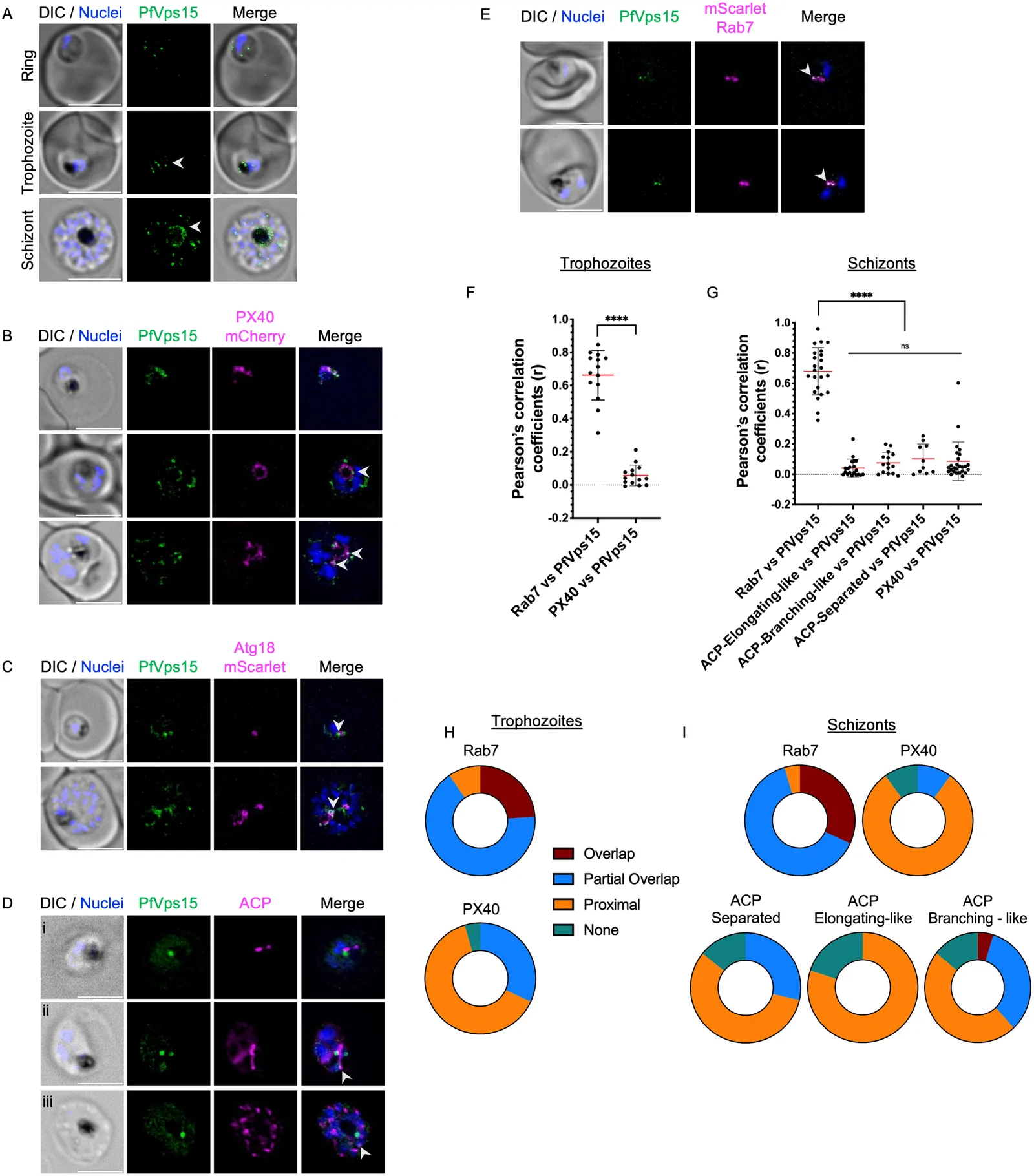
Subcellular localization of PfVps15. **A.** Endogenous expression of PfVps15-2xFKBP-mNeonGreen during the asexual parasite stages. White arrowheads indicate PfVps15 near the food vacuole in trophozoite and schizont stages. DIC: differential interference contrast. Blue: DAPI-stained nuclei: Merge: Merged DAPI, green and DIC channels. Scale bar: 5 μm. **B.** Live-cell imaging of PfVps15-2xFKBP-mNeonGreen with the PI3P sensor PX40-mCherry. **C.** Live-cell imaging of PfVps15-2xFKBP-mNeonGreen with PfAtg18-mScarlet. **D.** Immunofluorescence assay of PfVps15-2xFKBP-mNeonGreen with an antibody against the apicoplast marker PfACP. Apicoplast elongation (i), branching (ii) and separation (iii) are visible as schizont stage parasites progress. **E.** Live-cell imaging of PfVps15-2xFKBP-mNeonGreen with the endosomal marker mScarlet-PfRab7. **F.** Pearson’s correlation coefficients calculated in parasites during trophozoite stages demonstrate that PfVps15-2xFKBP-mNeon overlaps significantly more with the endosomal marker PfRab7 rather than PX40. PfVps15 vs PfRab7: n = 14; and PfVps15 vs PX40: n = 14. Values in the graph represent mean ± standard deviation. P values were calculated using an unpaired t-test. * P = 0.0322. **** P < 0.0001. **G.** Pearson’s correlation coefficients calculated in parasites during schizont stages demonstrate that PfVps15-2xFKBP-mNeonGreen overlaps significantly more with the endosomal marker PfRab7 than PX40 or ACP. PfVps15 vs PfRab7: n = 23; PfVps15 vs PfACP – Elongating-like: n = 20; PfVps15 vs PfACP – Branching-like: n = 15, PfVps15 vs PfACP – Separated: n = 10; and PfVps15 vs PX40: n = 26. Values in the graph represent mean ± standard deviation. P values were calculated using an unpaired t-test. **** P < 0.0001. ns > 0.9. **H.** Quantification of PfVps15-2xFKBP-mNeonGreen foci in relation to subcellular markers PfRab7 (n = 21) and PX40 (n = 22) during trophozoite stages. Foci were characterized as the following: Overlap (Red), Partial Overlap (Blue), Proximal (Orange) and None (Teal). **I.** Quantification of PfVps15-2xFKBP-mNeonGreen foci in relation to subcellular markers PfRab7 (n = 22), PfACP (elongating-like n = 25; branching-like n = 21; separated n = 10) and PX40 (n = 30) during schizont stages. Foci were characterized as the following: Overlap (Red), Partial Overlap (Blue), Proximal (Orange) and None (Teal). For B to E: White arrowheads highlight some areas of overlap between PfVps15-2xFKBP-mNeonGreen and the marker. DIC: Differential interference contrast. Blue: DAPI-stained nuclei; Merge: merged green, magenta and DAPI channels. Scale bar: 5 μm.

We next wanted to determine the localization of PfVps15-2xFKBP-GFP using live cell fluorescence microscopy. In rings and trophozoites, no/low fluorescence was observed whilst in schizonts, cytoplasmic foci could be seen (S1D Fig). To try to get a stronger fluorescence signal, we generated a parasite line where PfVps15 was tagged with 2xFKBP-mNeonGreen, as mNeonGreen is a brighter fluorescent protein (S1E Fig) [68]. Live-cell fluorescence microscopy showed the presence of PfVps15-2xFKBP-mNeonGreen cytoplasmic foci during all asexual parasite stages with a concentration around the FV in trophozoites and schizonts (Fig 2A). This is reminiscent of what was observed in previous studies using either an antibody against PfPI3K [32], PI3P sensors [18,35], the known PI3P-binding proteins PfAtg18, involved in FV dynamics and apicoplast biogenesis [17,69] and PfPX1, implicated in the HCC trafficking pathway [31]. To determine whether these foci overlapped with PI3P-containing membranes, we co-expressed the PI3P sensor PX40 fused to mCherry in the PfVps15-2xFKBP-mNeonGreen line (Fig 2B). This revealed that although some foci did colocalize, some did not, suggesting that potentially not all structures containing PfVps15 were labelled with PI3P or that if they were, the levels might be too low for detection with the sensor. The localization of PfAtg18 to the FV was shown to require PI3P [69] and interestingly, like for PX40-mCherry, only some foci of PfAtg18-mScarlet overlapped with PfVps15-2xFKBP-mNeonGreen, mostly around the FV (Fig 2C). PI3P being critical for apicoplast biogenesis [11,17,18,34,69], we next looked at the colocalization between PfVps15-2xFKBP-mNeonGreen and the apicoplast resident acyl-carrier protein (PfACP) [70]. We did not see any overlap in trophozoites, although some foci were in close juxtaposition (Fig 2Di). However, some PfVps15-2xFKBP-mNeonGreen-labelled structures seemed associated with the elongating apicoplast in developing schizonts, which could fit with a role for PI3P, potentially through the action of PfAtg18, in the elongation and segregation of the organelle [69] (Fig 2Dii). In schizonts, only some of the segregated apicoplasts seemed to overlap with PfVps15-2xFKBP-mNeonGreen (Fig 2Diii). The trafficking of host-cell cytosol to the FV follows an endosomal route with both conserved and parasite-specific adaptations [20,36], so we next wanted to see if PfVps15-2xFKBP-mNeonGreen localized to endosomal structures. Overlap was observed with some PfRab7-labelled structures (Fig 2E), a marker of late endosomes in typical eukaryotic cells [71,72]. PfRab7 was shown to localize near the FV [73], but whether it is involved in HCC trafficking is currently unknown. However, its nucleotide exchange factor PfSand1 was revealed to not be essential for the process [36]. We do note though that PfRab7 was found to be associated with PfPX1-containing foci, a protein with a demonstrated role in the HCC trafficking pathway [31]. No overlap was observed with the Golgi marker PfErd2, the rhoptry marker PfRAP1 nor the microneme marker PfEBA175 (S3 Fig). In order to quantify the levels of colocalization, we performed a Pearson’s correlation coefficient (PCC) analysis. During trophozoite stages, PfVps15-2xFKBP-mNeonGreen overlapped significantly more with the endosomal marker PfRab7 (PfRab7 vs PfVps15-2xFKBP-mNeonGreen: 0.66 ± 0.15) than with the FV marker PX40 (PX40 vs PfVps15-2xFKBP-mNeonGreen: 0.06 ± 0.06) (Fig 2F). PCCs obtained in trophozoite stages resembled those during schizont stages where PfVps15-2xFKBP-mNeonGreen once again overlapped most predominantly with the endosomal marker (PfRab7 vs PfVps15-2xFKBP-mNeonGreen: 0.68 ± 0.16) (Fig 2G). Little change is seen in PCCs for PX40 during schizont stages (PX40 vs PfVps15-2xFKBP-mNeonGreen: 0.09 ± 0.13). We also calculated PCCs for PfVps15-2xFKBP-mNeonGreen and PfACP during the different phases of apicoplast biogenesis (Elongating-like vs PfVps15-2xFKBP-mNeonGreen: 0.04 ± 0.06, Branching-like vs PfVps15-2xFKBP-mNeonGreen: 0.08 ± 0.07 and Separated vs PfVps15-2xFKBP-mNeonGreen: 0.1 ± 0.1) and found no difference with PX40 (Fig 2G). We noticed however, that the levels of foci overlap seemed to vary greatly between individual parasites, which was not reflected when looking at the averaged PCC values. We therefore classified the foci overlap into four categories: none representing no colocalization when there was no overlap between the magenta and green signals, proximal representing close foci when magenta and green signals were very close to each other (± less than one focus diameter apart) but did not overlap, partial overlap representing partial colocalization when some pixels from one green focus overlapped with pixels from a magenta focus, and finally, overlap representing when the foci extensively overlapped each other. As with the PCCs, during trophozoite stages, PfVps15-2xFKBP-mNeonGreen appeared to be in closer proximity to the endosomal marker PfRab7, where 23.8% of parasites displayed overlap of PfRab7 and PfVps15-2xFKBP-mNeonGreen signals and 66.67% of parasites had partial overlap (Fig 2H). The same analysis with PX40 revealed that while no parasites showed complete overlap of the PX40 and PfVps15-2xFKBP-mNeonGreen foci, the majority (63.6% of parasites) were in close proximity and 31.8% were partially overlapping. These tendencies continued during schizogony, with a slight increase in overlapping signals for PfRab7 (overlap: 31.8% and partial overlap: 63.6%) (Fig 2I). A shift was also seen with PfVps15-2xFKBP-mNeonGreen foci with regards to PX40, where only 10% of parasites had partially overlapping signals but 80% showed proximal foci. As for the apicoplast, when this organelle was elongating-like, 80% of parasites displayed PfVps15-2xFKBP-mNeonGreen foci in proximity to PfACP. When it was branching-like, 33.3% of parasites had overlapping PfVps15-2xFKBP-mNeonGreen and PfACP foci and in 47.6% of parasites these two signals are in proximity. Once the apicoplast is separated, 28.6% of the parasites had overlapping PfVps15-2xFKBP-mNeonGreen and PfACP foci and 57.1% of parasites displayed proximal signals. As with the PCC values, during both trophozoite and schizont stages, extensive overlap was only seen with PfRab7.

Collectively, these results show that while PfVps15-2xFKBP-mNeonGreen is present near the FV, where it is in close proximity and overlaps with the PI3P sensor PX40, PfVps15-2xFKBP-mNeonGreen can also be found at/near elongating and branching apicoplasts, a structure which is also enriched in PI3P. Finally, PfVps15-2xFKBP-mNeonGreen appears to localize more prominently with structures labelled with the endosomal marker PfRab7. It is tempting to speculate that these putative endocytic compartments represent a platform for PI3P generation.

### PfVps15 is essential for parasite proliferation during the erythrocytic cycle

**Fig 3:**
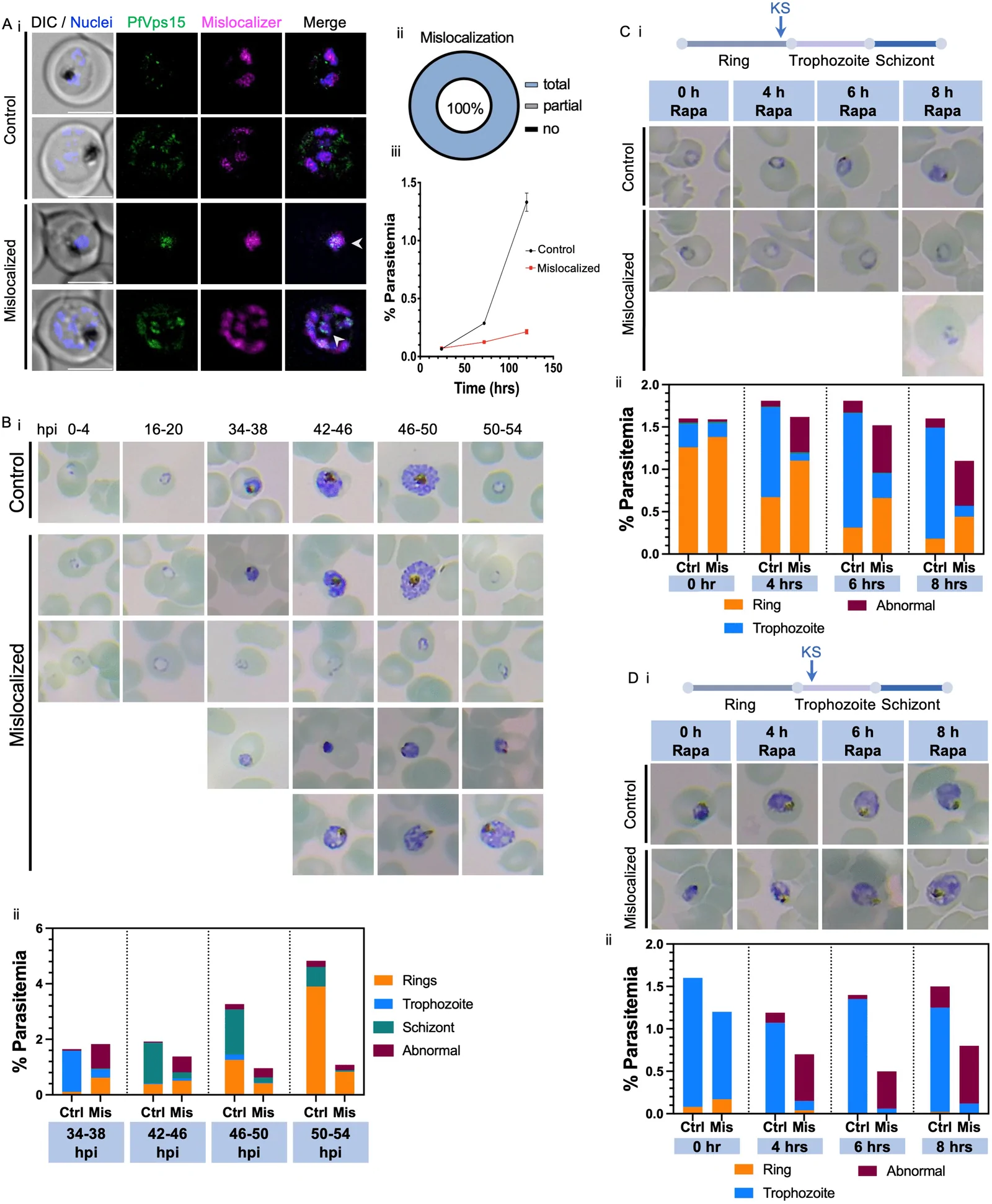
Conditional inactivation of PfVps15 by knock sideways leads to parasite death. **A.** (i) Fluorescence microscopy images of the PfVps15mis (PfVps15-2xFKBP-GFP + 1xNLS-FRB-mCherry mislocalizer) showing the translocation of the GFP signal to the nucleus after incubation with 150 nM of rapalog for 1 hour in comparison with a control without rapalog. Blue: DAPI stained nuclei. Merge: merged green, magenta and DAPI channels. Scale bar: 5 μm. (ii) Classification of the translocation levels as complete (all fluorescence overlapping with DAPI/mislocalizer), partial (some fluorescence overlapping with DAPI/mislocalizer) and no mislocalization. Results from 26 cells from two independent experiments. (iii) Growth curve of the PfVps15ctrl and PfVps15mis grown in the presence of rapalog. Control: PfVps15-2xFKBP-GFP. Mislocalized: PfVps15-2xFKBP-GFP+mislocalizer. Error bars: SD of three technical replicates. Data from the two additional biological replicates included in S4 Fig. **B.** (i) Light microscopy images of GIEMSA-stained smears of the PfVps15mis (mislocalized) and PfVps15ctrl (control) parasite lines after addition of rapalog at the early ring stage (0-4 hpi) taken at different times over one complete cycle. hpi: Hours post invasion. Images are representative of parasites of three experiments. (ii) Quantification of growth and parasite staging following the addition of rapalog. 300 parasites were scored in order to determine the proportion of rings, trophozoites, schizonts and abnormal parasites at each time point for both PfVps15mis and PfVps15ctrl lines. Parasitemia at each time point was determined by counting 1000 RBCs. Parasitemia and percentage of each category is represented. **C.** (i) Light microscopy images of GIEMSA-stained smears of the PfVps15mis (mislocalized) and PfVps15ctrl (control) parasite lines after addition of rapalog at the ring stage taken at different times over 8 hours. Addition of rapalog at the ring stage prevents the transition from ring to trophozoite in the mislocalizer line. (ii) Quantification of growth and parasite staging following addition of rapalog. 100 parasites were scored to determine proportion of rings, trophozoites, schizonts and abnormal parasites at each time point. Parasitemia was determined by counting 1000 RBCs. Parasitemia and percentage of each category is represented. **D.** (i) Light microscopy images of GIEMSA-stained smears of the PfVps15mis (mislocalized) and PfVps15ctrl (control) parasite lines after addition of rapalog at the trophozoite stage taken at different times over 8 hours. Addition of rapalog at the trophozoite stage results in the apparition of translucent regions within the parasite cytosol of the mislocalizer line. (ii) Quantification of growth and parasite staging following addition of rapalog. Between 72 and 99 parasites were scored to determine proportion of rings, trophozoites, schizonts and abnormal parasites at each time point. Parasitemia was determined by counting 1000 RBCs. Parasitemia and percentage of each category is represented.

Genome-wide inactivation screens and standard genetic inactivation attempts have suggested that both Vps15 and PI3K are essential for the proliferation of *P. falciparum* and the rodent model *P. berghei* [18,31,58,74]. Indeed, a recent study has shown that conditionally knocking out *PfPI3K* leads to parasite death [34]. To investigate the role of PfVps15, we employed the KS strategy that allows the conditional removal of a protein of interest from its site of action upon addition of rapalog to the culture medium [56]. To achieve this, the PfVps15-2xFKBP-GFP parasite line was transfected with an episome expressing a nuclear mislocalizer (NLS) fused to a FKBP12-binding domain, as well as mCherry as a fluorescent tag. To verify the ability of our system to cause the mislocalization of PfVps15 to the nucleus, thus removing it from its site of action, we added rapalog to one clonal line for one hour and then observed the parasites by fluorescence microscopy. In the absence of rapalog, PfVps15 is observed as cytosolic foci and around the FV and the mislocalizer colocalizes with the DAPI-stained nuclei whilst the addition of rapalog leads to the translocation of PfVps15 to the nuclei in all parasites observed (Fig 3Ai and 3Aii). This indicates that the KS of PfVps15 occurs rapidly and efficiently within the hour after the addition of rapalog. Following this confirmation, we next wanted to determine if the KS of PfVps15 affected parasite proliferation. To this end, we followed the growth by fluorescence-activated cell sorting (FACS) of tightly synchronous PfVps15-2xFBKP-GFP+mislocalizer parasites (named PfVps15mis hereafter) after the addition of rapalog at the ring stage. We used as control PfVps15-2xFBKP-GFP (referred to as PfVps15ctrl) with rapalog to take into account any potential deleterious effect of the molecule. The data revealed that the KS of PfVps15 leads to a severe growth defect demonstrating that the protein is critical for asexual proliferation (Figs 3Aiii and S4).

In order to further evaluate the function of PfVps15 and to determine which part of the asexual cycle was most affected by its inactivation, we analyzed parasite development using GIEMSA-stained smears following the addition of rapalog. When adding rapalog during the early ring stage (0–4 hpi), the PfVps15ctrl strain grew normally as seen in the first row of Fig 3Bi, progressing from ring to trophozoite to schizont and expanding to the second cycle. However, only a small fraction of the PfVps15mis parasites developed normally (Fig 3Bi, Mislocalized row 1). Indeed, while 89% of the PfVps15ctrl were at the trophozoite stage at 34–38 hpi, only 16% of the PfVps15mis were trophozoites. 33.7% were still at the ring stage (Fig 3Bi, Mislocalized row 2), and 48.7% presented as dark parasites without visible hemozoin crystals which we termed “abnormal” (Fig 3Bi, Mislocalized row 3). The trophozoites in PfVps15mis appeared to progress to schizont stage at 42–46 hpi (also about 16%), while 76.7% of PfVps15ctrl were at this stage. At this time point, 41.7% of PfVps15mis were also classified as abnormal. Four different abnormal presentations appeared and remained for the duration of the experiment. Small trophozoite-like parasites with no or very little hemozoin (Fig 3Bi, Mislocalized row 3) were often visible, as were misshapen ring stage parasites (Fig 3Bi, Mislocalized row 2). Also, it was at this time that the first parasites with many translucent areas in their cytoplasm appeared (Fig 3Bi, Mislocalized row 4). However, the bulk of this phenotype was visible at 50–54 hpi, while the control parasite line had begun their second cycle. The percentage of ring stage parasites remained relatively constant in the PfVps15mis parasite line at 42–46 hpi (36.7%) and 46–50 hpi (42.33%) and only increasing significantly at 50–54 hpi (76.33%). These likely are a combination of stalled rings from the first cycle along with newly formed rings from the portion of parasites that succeeded in progressing to schizonts. For the control, an increase in ring stage parasites was observed at 46–50 hpi (38.3%) and 50–54 hpi (80.7%) as expected as more parasites transitioned from schizonts into newly formed rings. The total parasitemia of the PfVps15mis line was 1.08% whilst it was 4.83% for the control line at the 50–54 hpi time point, correlating with the deficiency in parasite proliferation. We next wanted to more closely monitor the transition of ring stage parasites to the trophozoite stage, so we added rapalog to late rings and analyzed growth over 8 hours by GIEMSA-stained smears (Fig 3Ci). In the control line (PfVps15ctrl), 82% of the parasites progressed normally from rings to trophozoites at the end of the assay period. However, only 12% of the PfVps15mis parasites transitioned to the trophozoite stages with a visible FV. 40% were still rings whilst 48% were darker cells with a translucent vesicular structure which we classified as abnormal (Fig 3Cii). This stalled/delayed transition from the ring to the trophozoite stage is reminiscent of what was seen with other proteins involved in the HCC trafficking pathway [25,30,31,36]. When rapalog was added to trophozoite stage parasites (Fig 3Di), the control progressed normally through the trophozoite stage heading towards schizogony (after 8 hours, 82% were larger trophozoites). However, after 4 hours, 79% of PfVps15mis parasites showed an accumulation of seemingly translucent areas within the cells which slightly increased after 8 hours (85%), reminiscent of the HCC-filled vesicles commonly seen when proteins that act in the later stages of the HCC trafficking pathway are inactivated [25,26,30,31,36](Fig 3Dii). As the assay progressed, we also noticed the appearance of PfVps15mis parasites that did not seem to be inside red blood cells anymore. As we did not count them in the abnormal, this potentially explains why the total parasitemia decreased during the assay period. One of the potential explanations could be that this is due to increased osmotic fragility.

### Inactivation of PfVps15 during the trophozoite stage abrogates the delivery of host-cell cytosol filled vesicles to the food vacuole

**Fig 4:**
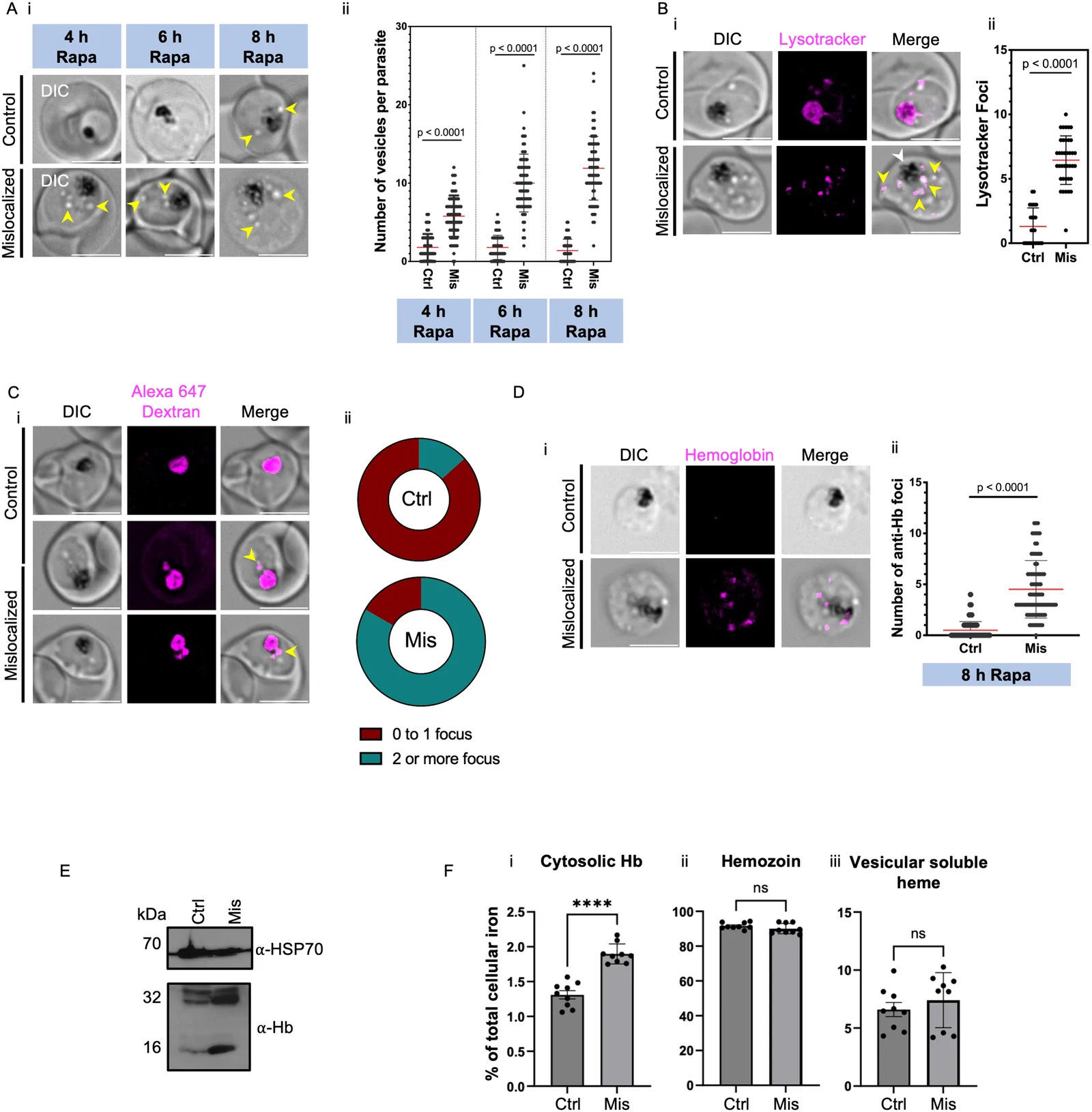
The knock sideways of PfVps15 at the trophozoite stage results in the accumulation of host-cell cytosol-filled vesicles with endocytic characteristics. **A.** (i) Differential interference contrast (DIC) images after addition of rapalog for 4, 6 and 8 hours to the PfVps15mis and PfVps15ctrl parasite lines. Accumulation of vesicle-like structures (yellow arrowheads) are visible in the mislocalizer line but largely absent from the control. DIC images are representative of what was observed during two independent experiments. Scale bar: 5 μm. (ii) Quantification of the number of vesicles per cell determined using DIC images for the PfVps15mis (4 hours: n = 74; 6 hours: n = 83; 8 hours: n = 80) and the PfVps15ctrl without the mislocalizer (Ctrl) (4 hours: n = 78; 6 hours: n = 78; 8 hours: n = 81) combined from two independent experiments. Red line: mean; Error-bars: SD. P-values determined by unpaired two-tailed t-tests. **B.** (i) Fluorescence microscopy of Lysotracker-stained PfVps15mis and PfVps15ctrl parasite lines following incubation with rapalog for 6 hours. Yellow arrowheads indicate the presence of Lysotracker staining within accumulated vesicles visible in DIC in the Vps15mis parasite cytosol. White arrowhead shows the lack of marker staining in the FV. Merge: merged magenta channel and DIC. Scale bar: 5 μm. (ii) Quantification of the number of Lysotracker positive foci per cell determined by fluorescence microscopy images following incubation with rapalog for 6 hours. PfVps15mis: n = 38 and PfVps15ctrl: n = 26. Red line: mean; Error-bars: SD. P-values determined by unpaired t-tests. **C.** (i) Fluorescence microscopy and differential interference contrast images (DIC) of PfVps15ctrl and PfVps15mis parasite lines grown in red blood cells preloaded with Alexa Fluor-647-labelled dextran. Yellow arrowheads indicate the presence of dextran within accumulated vesicle-like structures in the Vps15mis parasite cytosol. Merge: merged magenta channel and DIC. Scale bar: 5 μm. Representative images from two independent experiments. (ii) Quantification of parasites containing Alexa Fluor-647-labelled dextran HCC-uptake intermediates for both the PfVps15mis (n = 60) and PfVps15ctrl (n = 60) parasite lines. Parasites were classified as containing either 1 or less (red), or 2 or more foci (teal), excluding larger FV associated focus. Red line: mean; Error-bars: SD. P-values determined by unpaired t-tests. **D.** (i) Anti-hemoglobin immunofluorescence assays on the PfVps15mis and PfVps15ctrl parasite lines following incubation with rapalog for 6 hours. Merge: merged magenta channel and DIC. Images are representative of two independent experiments. Scale bar: 5 μm. (ii) Quantification of the number of hemoglobin foci per cell determined by IFA for the PfVps15mis: n = 85 and PfVps15ctrl: n = 69 parasite lines upon incubation with rapalog. Data pooled from two independent experiments. P-values determined by unpaired two-tailed t-tests. **E.** Western Blot analysis of the intracellular hemoglobin content of saponin-freed PfVps15mis and PfVps15ctrl parasite lines following addition of rapalog during the trophozoite stage for 6 hours. PfHSP70 is used as a loading control for both PfVps15mis and PfVps15ctrl. An increase in Hb levels within the PfVps15mis parasite line is observable. WB is representative of two independent experiments. **F.** Heme fractionation assay of PfVps15mis and PfVps15ctrl parasite lines following incubation of trophozoites with rapalog for 6 hours. Graphs represent three independent experiments completed in triplicate (PfVps15ctrl: n = 9; PfVps15mis: n = 9). P-values were determined by two-tailed unpaired t tests. ****: p-value<0.0001. ns: non-significant. (i) Proportion of total cellular iron composed of cytosolic hemoglobin (Hb). (ii) Proportion of total cellular iron composed of hemozoin. (iii) Proportion of total cellular iron composed of FV-associated hemoglobin and free heme.

The localization of PfVps15 near the FV, its interaction with PfPI3K, as well as the accumulation of vesicular structures upon its KS, suggested that PfVps15 could potentially play a role in the trafficking pathway of HCC from the cytostome to the FV. To explore this possibility, we quantified the number of vesicles resulting from the inactivation of PfVps15 in trophozoites by differential interference contrast (DIC) (Fig 4Ai). After 4 hours in the presence of rapalog, the inactivation of PfVps15 led to an average of 5.8 ± 2.6 vesicles per parasites compared to 1.8 ± 1.7 vesicles in the control parasite line. After 6 hours, the PfVps15mis parasites had an average of 10 ± 3.7 vesicles per parasites compared to 1.8 ± 1.6 vesicles in the PfVps15ctrl parasite line. Finally, after 8 hours, the PfVps15mis parasites had an average of 11.9 ± 4.1 vesicles per parasites compared to 1.4 ± 1.5 vesicles in the control parasite line (Fig 4Aii). These results show that the KS of PfVps15 results in an increased accumulation of vesicles as time progresses, which are rarely observed in the control parasite line. We next wondered if these vesicles had endosomal characteristics, so we incubated the parasites with Lysotracker, a marker of acidified compartments that is known to label endocytic vesicles containing HCC [23]. In control cells, the FV and a few cytosolic foci were labelled whilst in the PfVps15mis, a much higher number of foci were observed, some of which overlapped with vesicular structures seen in DIC (Ctrl:1.31 ± 1.44 vs Mis: 6.45 ± 1.88) (Fig 4Bi and 4Bii). Interestingly, the FV was no longer labelled by Lysotracker in the PfVps15mis parasites, suggesting FV deacidification, like what was observed in a parasite line where the subunit B of the V-ATPase was conditionally knocked out [75]. Whilst the major proportion of the V-ATPase is found in the FV membrane, it also localizes to putative vesicular structures inside the parasite cytosol, potentially representing trafficking intermediates on their way to fuse with the FV membrane [75,76] as shown for a number of FV resident proteins like Plasmepsin 2 and Falcipain 2 [77–79]. It is tempting to speculate that the deacidification observed in PfVps15mis line is a result of the failed delivery of V-ATPase subunits to the FV membrane which would in turn reduce proton influx into the organelle. Potential mistrafficking of ion channels and transporters might also lead to increased proton leakage and FV destabilization [80]. Of note, previous work has shown FV destabilization in PI3K-inhibitor treated parasites subjected to heat stress but not in parasites grown at normal temperature and further revealed that PfHsp70–1, a PI3P-binding protein, was important in the maintenance of FV integrity [81]. Taken together, these data demonstrate that the accumulated vesicles have endocytic characteristics. To determine whether the vesicles contained HCC, we imaged parasites grown in RBCs preloaded with fluorescent Dextran. In the control parasites, the FV was intensely labelled however, in the PfVps15mis line, additional foci could be seen, some of which overlapped with vesicular structures seen in DIC (Fig 4Ci and Fig 4Cii). Upon further analysis, we were able to determine that in the PfVps15mis parasite line, 83.33% of parasites contained at least 2 additional foci, whilst the PfVps15ctrl parasite line, this was the case for only 13.33% of parasites.

**Fig 5:**
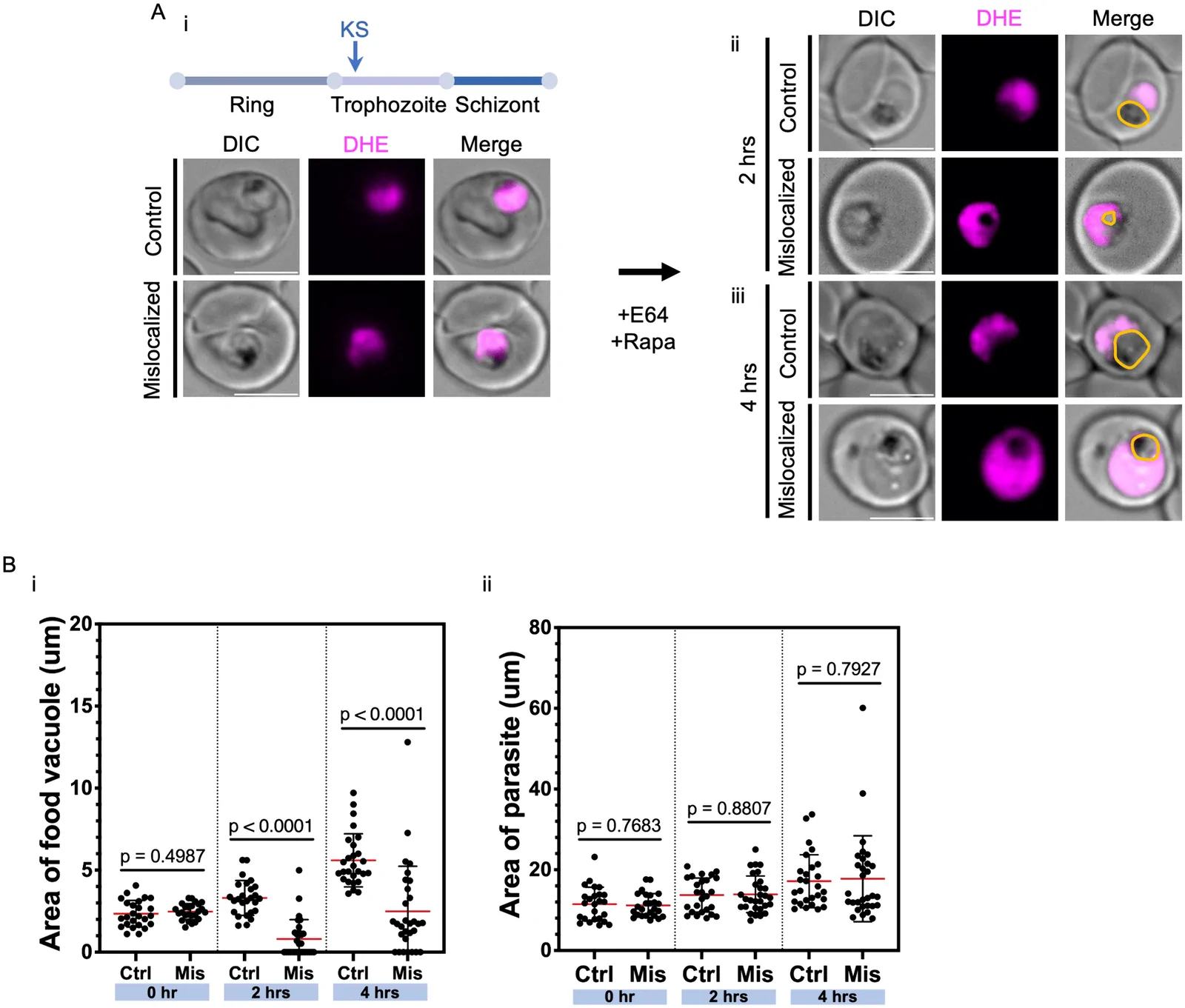
The knock sideways of PfVps15 at the trophozoite stage abrogates the delivery of host cell cytosol filled vesicles to the food vacuole. **A.** Fluorescence microscopy and differential interference contrast images of the PfVps15mis and PfVps15ctrl parasite lines incubated with rapalog before (i) and following a 2 and 4-hour treatment with the protease inhibitor E64 initiated during the early trophozoite stage (ii). Dihydroethidium (DHE) is used to stain the parasite cytoplasm to facilitate visualization of the food vacuole. Images are representative of two independent experiments. Yellow circles highlight food vacuoles. Merge: merged magenta channel and DIC. Scale bar: 5 μm. **B.** (i) Surface area of the food vacuoles of the PfVps15mis and PfVps15ctrl parasite lines incubated with rapalog following a 2 and 4-hour E64 treatment initiated during the early trophozoite stage. Each dot represents the value from one cell. (0 hour, PfVps15mis: n = 25 and PfVps15ctrl: n = 25; 2 hours, PfVps15mis: n = 29 and PfVps15ctrl: n = 26; 4 hours, PfVps15mis: n = 30 and PfVps15ctrl: n = 28). Data from two independent experiments are pooled. Red line: mean; Error-bars: SD. P-values determined by unpaired two-tailed t-tests. (ii) Total surface area of the parasites of the PfVps15mis and PfVps15ctrl parasite lines incubated with rapalog following a 2 and 4-hour E64 treatment initiated during the early trophozoite stage. Each dot represents the value from one cell. (0 hour, PfVps15mis: n = 25 and PfVps15ctrl: n = 25; 2 hours, PfVps15mis: n = 29 and PfVps15ctrl: n = 26, 4 hours, PfVps15mis: n = 30 and PfVps15ctrl: n = 28). Data from two independent experiments are pooled. Red line: mean; Error-bars: SD. P-values determined by unpaired two-tailed t-tests.

We also conducted IFAs using an anti-hemoglobin antibody, after the release of the HCC by saponin lysis (Fig 4Di). This showed an increase in the number of Hb foci in the PfVps15mis line with an average of 4.5 ± 2.8 foci per parasites whereas the control parasite line only contained on average 0.5 ± 4.1 foci per parasite (Fig 4Dii). When looking at the DIC images, we noticed that the vesicles were much harder to differentiate than when looking at live cells which might potentially be explained by the fact that the parasites are first treated with saponin and then fixed. Moreover, as previously seen in studies of proteins involved in HCC trafficking or trafficked through this pathway [30,36,69,82], few of the vesicles overlapped with Hb foci, suggesting that there are perhaps distinct populations of vesicles. This increase in Hb-containing vesicles in the PfVps15mis line translated to a higher amount of parasite associated-Hb as shown by Western blot (Fig 4E). Furthermore, heme fractionation assays revealed an increase in the proportion of cytosolic heme (in the form of Hb) compared to total cellular iron (PfVps15mis: 1.90% ± 0.05 vs Control: 1.31% ± 0.06) (Fig 4Fi), in line with the increase in the number of Hb foci observed by IFA. Whilst there was a small reduction in hemozoin, it was not statistically significant (PfVps15mis: 90.05% ± 0.99 vs Control: 91.65% ± 0.59). There was also no statistically significant difference in the FV-associated soluble heme (PfVps15mis: 7.40% ± 0.79 vs Control: 6.60% ± 0.60) (Fig 4Fii). Finally, to directly explore whether the delivery of HCC-containing vesicles to the FV was compromised, we performed a bloated FV assay by incubating parasites with the protease inhibitor E64 (Fig 5A). This treatment prevents the digestion of Hb in the FV, leading to an increase in its size as more and more Hb is delivered to the organelle. When Hb vesicle delivery is affected, bloating is decreased [83]. Incubation of the PfVps15ctrl parasites for 2 hours with E64 led to bloated FVs whilst it was not seen in the PfVps15mis line and this was further exacerbated after 4 hours (Fig 5B). To confirm that the lack of bloating in the KS line was not due to parasite death, we measured the cell surface area and saw that it increased to the same levels than the control (Fig 5C). Taken together, these results show that the inactivation of PfVps15 leads to the accumulation of vesicle-like structures with endocytic characteristics in the parasite cytoplasm due to a defect in their delivery to the FV and that some of these vesicles contain Hb.

### Inactivation of PfVps15 during the late trophozoite stage leads to defects in apicoplast dynamics

**Fig 6:**
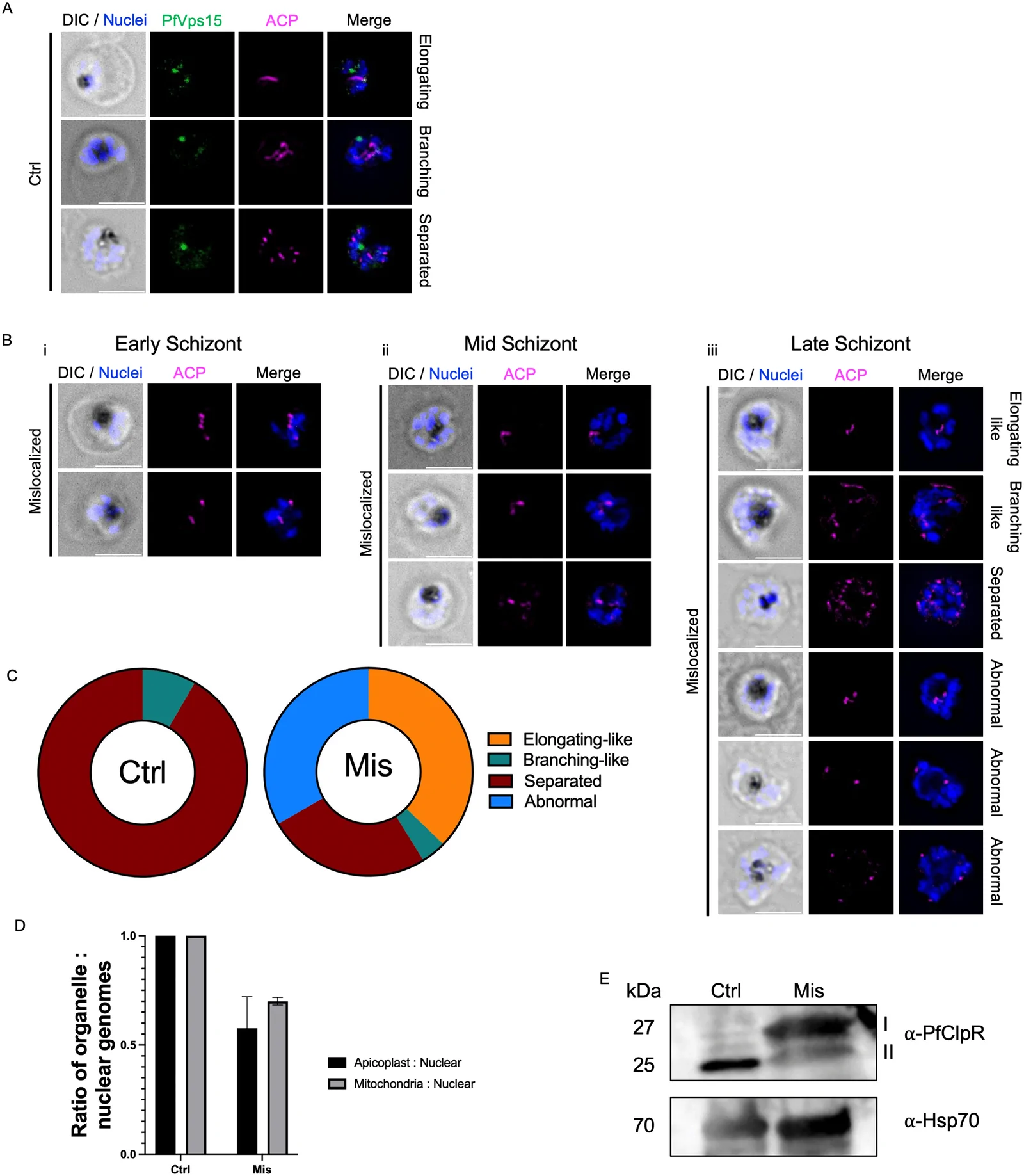
The inactivation of PfVps15 in late trophozoites causes a defect in apicoplast dynamics during schizogony. **A.** Immunofluorescence assays showing the development of the apicoplast during schizogony in the PfVps15ctrl line. Apicoplast elongation, branching and separation are visible as schizont stage parasites progress. Rapalog added in late trophozoite stage. Ctrl: Control. Blue: DAPI stained nuclei. Merge: merged green, magenta and DAPI channels. Scale bar: 5 μm. **B.** Immunofluorescence assays showing that apicoplast biogenesis is disrupted during schizogony in the PfVps15mis parasite line. Examples of different phenotypes observed are shown. Rapalog added in late trophozoite stage. Blue: DAPI stained nuclei. Merge: merged magenta and DAPI channels. Scale bar: 5 μm. Representative images from two independent experiments. **C.** Classification of apicoplast developmental stages for both PfVps15mis (n = 51) and PfVps15ctrl (n = 60) parasite lines. Rapalog added in late trophozoite stage. Apicoplast stages were classified into 4 categories: Elongating-like (orange), Branching-like (teal), Separated (red) and Abnormal (blue). Results from two independent experiments combined. **D.** Analysis of organellar genome replication. Tightly synchronized PfVps15mis and PfVps15ctrl parasites received rapalog treatment for 6 hours following which genomic DNA samples were isolated in order to perform real-time qPCR analysis to determine apicoplast: nuclear genome ratio and mitochondria: nuclear genomes ratios. To obtain genome ratios, results were normalized to the PfVps15ctrl parasite line. Data in graph represent means and SD. Results from three independent experiments combined. **E.** Western Blot analysis of the import of the apicoplast resident protein PfClpR in PfVps15mis and PfVps15ctrl parasite lines following addition of rapalog during the schizont stage for 6 hours using an anti-PfClpR antibody. I: Protein containing transit peptide. II: Cleaved processed protein. PfHSP70 is used as a loading control for both PfVps15mis and PfVps15ctrl. An accumulation of unprocessed PfClpR is visible in the PfVps15mis parasite line.

In addition to the FV membrane and potential vesicular trafficking intermediates, PI3P is also found at the apicoplast in *Plasmodium* parasites [18,35] and also in *T. gondii* [84]. In both species, conditional abrogation of their respective PI3K leads to defects in apicoplast biogenesis [34,40], demonstrating a critical role for PI3P in this process. To explore whether PfVps15 played a role in apicoplast biology, we induced the KS in late trophozoites and followed the development of the apicoplast during schizogony by IFA using an anti-PfACP antibody. When looking at the control line, the apicoplast elongated in early schizonts, branched and subsequently separated in individual merozoites in late schizonts (Fig 6A), as expected [85]. As describe earlier, some of the PfVps15 signal was in close proximity to/overlapped with ACP during the elongation and branching phases (Figs 2D and 6A). In the PfVps15mis line, elongating and branching apicoplasts were rarely observed (Fig 6B). Instead, in early and mid-schizonts, PfACP was seen more as foci, some of which sometimes seemed to be linked by a faint ribbon of fluorescence (Fig 6Bi and Fig 6Bii). In late schizonts, each merozoite should have inherited a single apicoplast. However, this seemed the case for only 25.5% of the observed schizonts (Fig 6Biii and 6C). The remaining 74.5% of cells showed a variety of abnormal apicoplast-types for late schizonts with some parasites having what looked like an apicoplast in the process of elongating (37.3%), others that somewhat seemed like branching (3.9%) and some with only a few merozoites containing individual fluorescent foci (labelled as abnormal, 33.3%). In the elongating-like and branching-like apicoplasts, the signal was always much more granular than in the control, i.e., we never saw a nice, continuous apicoplast which suggests that the process was at least partially perturbed. In order to further characterize this, we looked whether the replication of the apicoplast genome was affected in the PfVps15mis parasite line. We therefore performed qPCR in order to determine organelle to nuclear ratios following rapalog treatment during schizogony. Concurrent with a default in branching and division of the apicoplast in the PfVps15mis parasite line that we observed in microscopy, the apicoplast to nuclear genome ratio was much reduced (organelle to nuclear genome ratio of 0.57 ± 0.14) when compared to the PfVps15ctrl parasite line (Fig 6D). Interestingly, whilst less affected, replication of the mitochondrial genome was also diminished (organelle to nuclear genome ratio of 0.7 ± 0.02). This will be further explored later on in the manuscript. Finally, we investigated whether protein trafficking and import into the apicoplast was also compromised. Apicoplast proteins are for the most part encoded in the nuclear genome and possess a transit peptide that is processed only upon reaching the organelle. Thus, a defect in protein import into the apicoplast affects transit peptide cleavage [86]. When apicoplast proteins are not able to be imported into the organelle, an accumulation of the uncleaved protein is visible by Western Blot. Using an antibody against PfClpR, an apicoplast resident protein [87,88], we indeed observed an increase in the unprocessed 27 kDa form still containing its transit peptide compared to the mature 25 kDa form following rapalog treatment of the PfVps15mis parasite line (Fig 6E). This demonstrates that the PfVps15mis line has a defect in protein import into the apicoplast. Previous studies showed that treating parasites complemented with isopentenyl pyrophosphate with certain antibiotics like chloramphenicol [89], doxycycline [90] or azithromycin [91], or where the Suf Iron-Sulfur synthesis pathway is disrupted by the expression of a dominant-negative mutant of PfSufC [92], led to apicoplast disruption and multiple apicoplast-like vesicles. However, these apicoplast-like vesicles are not visually similar to the foci we observe in our PfVps15mis line since they are much more numerous and often much smaller. This is also different than in *T. gondii* PI3K knocked down parasites where the apicoplast becomes enlarged [40]. The apicoplast of *P. falciparum* is highly enriched in phosphatidylinositol [93], which could potentially be used by the PfVps15-PfPI3K complex to synthesize PI3P *in situ*. The apicoplast issues in our PfVps15mis line could potentially be due a defect in the PI3P-dependent delivery of proteins and lipids by vesicular fusion. It is possible that the heterogeneity in the phenotype observed by microscopy is due to differences in either the efficacy of the KS in individual parasites or perhaps in the levels of PI3P already present upon induction of the KS. Perhaps the more PI3P already present at their apicoplast, the more the organelle can develop until it reaches a stage where there is no longer enough PI3P to continue the process. It could also be that trafficking intermediates containing PI3P generated at endocytic-like compartments are needed to deliver cargo to the developing apicoplast. The stronger colocalization of PfVps15 with structures labelled with endocytic markers we observed gives support to this second hypothesis. Recent work has identified a *Plasmodium*-specific protein, which the authors named PfAnchor, that was shown to be critical for apicoplast segregation [94]. In a parasite line where PfAnchor was knocked down, the apicoplast was able to elongate and branch however, there was no separation. This resulted in some merozoites linked by an undivided apicoplast and others without the organelle. Of interest, PfAnchor possesses a PH domain containing a putative PIP-binding motif. Although it is currently unknown whether PfAnchor is a true PIP-binding protein, it is tempting to speculate that it might utilize this motif to bind PI3P on the surface of the apicoplast. This would imply that the lipid might have a role both in the expansion of the apicoplast and in its subsequent fission.

### Inactivation of PfVps15 during the late trophozoite stage leads to defects in mitochondrial fission

**Fig 7:**
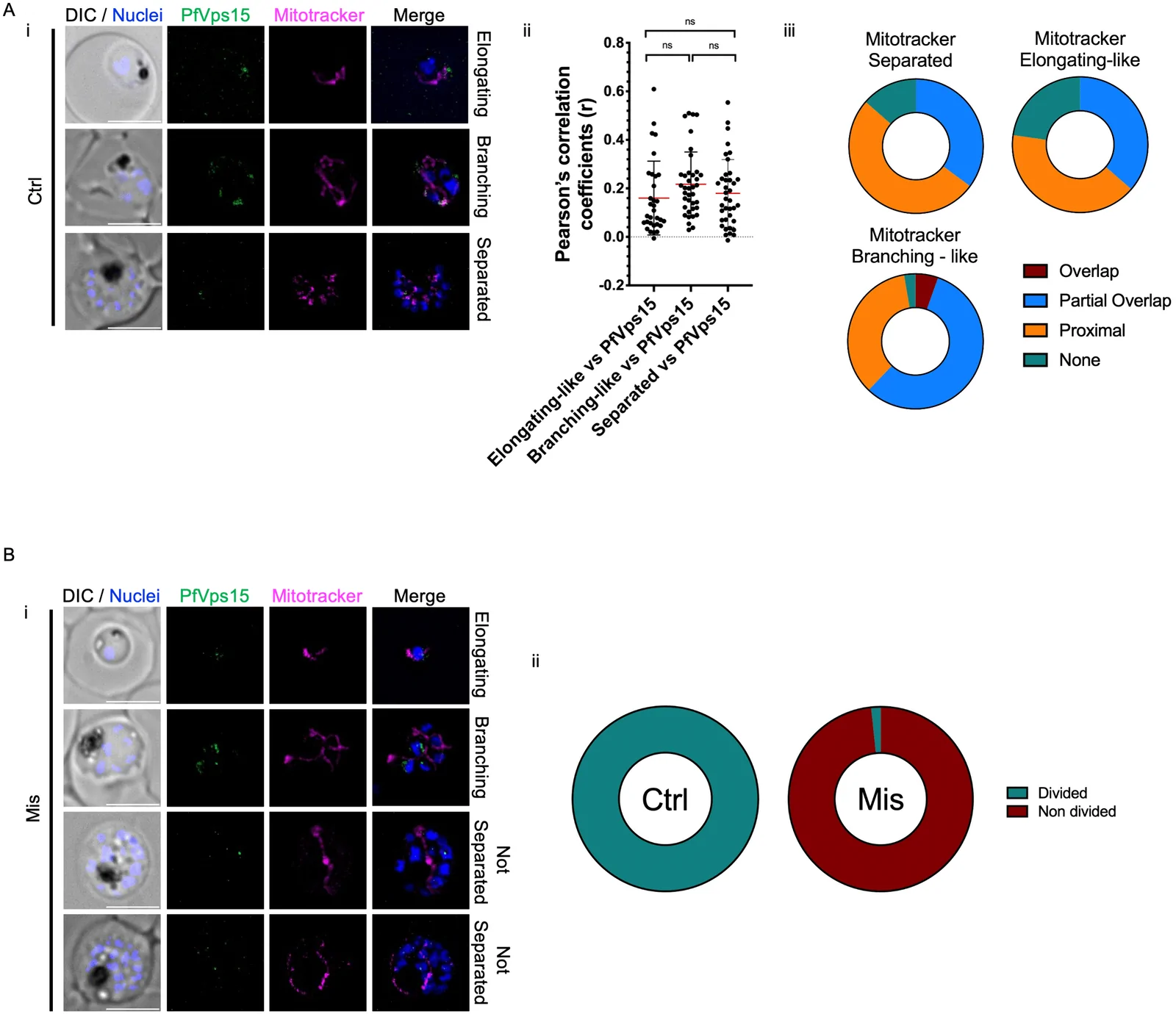
The inactivation of PfVps15 in late trophozoites causes a defect in mitochondrial fission. **A.** (i). Live microscopy with Mitotracker staining showing the development of the mitochondrion during schizogony in the PfVps15ctrl line. Mitochondrion elongation, branching and separation are seen as the parasites progress through the schizont stage. Ctrl: Control. Blue: DAPI stained nuclei. Merge: merged green, magenta and DAPI channels. Scale bar 5 μm. (ii) Pearson’s correlation coefficients quantifying the overlap between PfVps15-2xFKBP-GFP and Mitotracker during elongating-like, branching-like and separated phase of mitochondrial growth. PfVps15 vs Elongated mitochondrion: n = 32; PfVps15 vs Branched mitochondrion: n = 38; and PfVps15 vs Separated mitochondrion: n = 38. Values in the graph represent de mean ± standard deviation. Red line: mean; Error-bars: SD. P values were calculated using an unpaired t-test. ns > 0.9. (iii) Classification of the overlap between PfVps15-2xFKBP-GFP and Mitotracker. Data from Fig 7A (ii) used. Foci were characterized as the following: Overlap (Red), Partial Overlap (Blue), Proximal (Orange) and None (Teal). **B.** (i) Live microscopy with Mitotracker staining showing that the fission of the mitochondria does not occur during schizogony in the PfVps15mis line. Mitochondria elongation and branching are seen, but not separation as the parasites progress through the schizont stage. Rapalog added in late trophozoite stage. Ctrl: Control. Blue: DAPI stained nuclei. Merge: merged green, magenta and DAPI channels. Scale bar 5 μm. (ii) Classification of the state of the mitochondrion at the end of schizogony for both PfVps15mis (n = 60) and PfVps15ctrl (n = 60) parasite lines following incubation with rapalog to initiate KS. Mitochondria were classified as either separated or not. Results from two independent experiments combined.

Our finding that the replication of the mitochondrial genome was impacted following the KS of PfVps15 was initially puzzling since a role for PI3P in mitochondrial dynamics was unknown until a recent study uncovered a role for PI3P in mitochondrial biology in *Caenorhabditis elegans* [95]. Mutants of the PI3P-binding protein EXC-5 or the VPS34 PI3K had mitochondria that were unable to elongate or bud. The authors demonstrated that the binding of EXC-5 to PI3P on early endosomes resulted in their association with the mitochondrion which led them to speculate that EXC-5 might promote endosome-mitochondrion contact sites. To determine whether PfVps15 was important for *P. falciparum* mitochondrial dynamics, we induced the KS of the protein in late trophozoites and looked at the development of the organelle throughout schizogony. Interestingly, whilst looking at the development of the mitochondrion in the control parasite line, we noticed that we often observed PfVps15-2xFKBP-GFP at/near the Mitotracker signal. We quantified the levels of colocalization by PCC analysis throughout schizogony and found that PfVps15-2xFKBP-GFP overlapped in a similar manner during all stages of mitochondrial growth (Mitotracker Branching-like vs PfVps15: 0.22 ± 0.13; Mitotracker Elongating-like vs PfVps15: 0.16 ± 0.15; Mitotracker Separated vs PfVps15: 0.18 ± 0.14) (Fig 7Aii). As with the previous colocalization analysis, we wanted to make sure that these PCCs properly described what was observed since the levels of overlap again seemed to vary greatly between individual parasites. We therefore classified the overlap into the same four categories: none representing no colocalization when there was no overlap between the magenta and green signals, proximal representing close foci when magenta and green signals were very close to each other (± less than one focus diameter apart) but did not overlap, partial overlap representing partial colocalization when some pixels from one green focus overlapped with pixels from a magenta focus, and finally, overlap representing when the foci extensively overlapped each other. PfVps15-2xFKBP-GFP was more proximal to/overlapped more with the Mitotracker when the mitochondria are branched. Indeed, it was the only time where strong overlap was observed. During this stage, 5.4% of parasites displayed overlap, 56.8% partial overlap and 35.1% displayed proximal PfVps15-2xFKBP-GFP and Mitotracker signals. Once separated mitochondria are present, 35.1% of parasites had partially overlapping PfVps15-2xFKBP-GFP and Mitotracker foci and 51.4% had proximal foci. During the elongating-like stage of mitochondrial growth, 36.4% of parasites had partially overlapping signals and 40.9% displayed proximal PfVps15-2xFKBP-GFP and Mitotracker signals (Fig 7Aiii). We next looked at the status of the mitochondrion in late schizonts of the PfVps15 mis line. Since mitochondrial fission occurs late in schizogony, we incubated parasites with the egress inhibitor E64 to make sure that any potential phenotype observed was not due to a delay in schizogony. Whilst there did not seem to be a defect in mitochondrial expansion, almost all the observed schizonts had an undivided mitochondrion in the PfVps15 mis line (Fig 7Bi). Indeed, upon closer observation, we noted that 100% of the control parasites had divided mitochondria upon late schizogony, and only 1.67% of the PfVps15mis parasites had divided mitochondria at the same stage (Fig 7Bii). This suggests a previously unrecognized role for PI3P in mitochondrial fission in *P. falciparum*. Whilst ER-mitochondria [85,96,97] and ER-IMC [97,98] contact sites have been demonstrated in *P.* *falciparum*, the existence of endosome-mitochondria contact sites has not yet been reported. It would be interesting to look at parasites tagged with mitochondrion and endosomal markers to explore whether contact sites can be visualized. In mammalian cells, it has been proposed that Rab7-labelled late endosomes could be implicated in this mechanism, localizing transiently at the mitochondrion, marking fission sites [99]. This makes the high level of colocalization that we observe between PfRab7 and PfVps15 interesting in this context. EXC-5 contains a RhoGEF, two PH and a FYVE domain. Although *P. falciparum* possesses several proteins with a PH domain [100] and two proteins with a FYVE domain [33,36], none combine all three domains suggesting that there is no direct orthologue of EXC-5 [101]. The specific recruitment of EXC-5 via these domains through interaction with PI3P was proven to be necessary for the subsequent recruitment of Dynamin-related protein 1 (DRP1) [102]. DRP1 and Dynamin 2 (Dyn2) act together to complete mitochondrial fission in mammalian cells. While DRP1 constricts the mitochondrial membrane, transiently associated Dyn2 completes mitochondrial fission [103]. However, a DRP1 orthologue is not present in *P. falciparum*. Interestingly, recent work demonstrated that PfDyn2 was essential for the proper division of both the mitochondrion and the apicoplast [104,105]. The authors showed that in PfDyn2-deficient parasites, the mitochondrion was able to grow in size and branched but remained this way throughout schizogony. This closely resembles what is seen following the KS of PfVps15. Interestingly, PfDyn2 (PF3D7_1037500) was found in one of the replicates of the PfVps15-2xFKGP-GFP immunoprecipitation, suggesting a potential interaction (S1 Table). It is tempting to speculate that PI3P might be implicated in proper mitochondrial fission by possibly recruiting PfDyn2 to the mitochondrion, via an accessory protein, as PfDyn2 lacks the PH domain usually present in Dynamin-like proteins [106]. Dyn2 recruitment factors in general are still largely unknown. Perhaps there is an as of yet unidentified PI3P-binding protein that could potentially be implicated in the proper localization of PfDyn2 to the mitochondrion, much as what was discovered with PfAnchor being critical for the association of PfDyn2 to the apicoplast [94]. The development of the mitochondrion and the apicoplast being tightly coupled, with the latter segregating before [85,107], it could also be that the mitochondrial fission defect arises secondarily to abrogated apicoplast development although this would not necessarily be mutually exclusive with a direct role for PI3P.

### Inactivation of PfVps15 during the trophozoite stage leads to a decrease in PI3P levels

**Fig 8:**
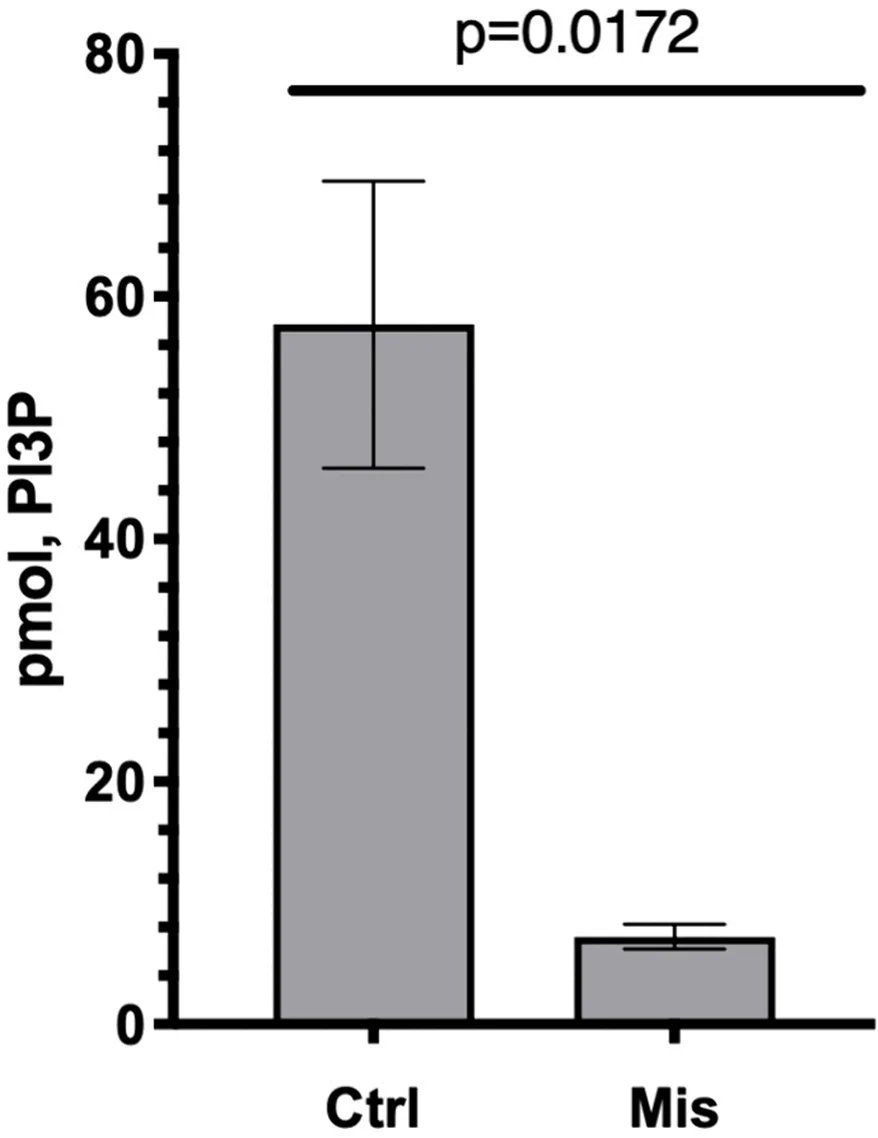
The inactivation of PfVps15 during the trophozoite stage causes a decrease in the production of PI3P. Quantification of cellular PI3P levels by PI3P Mass ELISA in trophozoite stages of PfVps15mis and PfVps15ctrl parasites after a 6-hour rapalog treatment. Graph represents average PI3P concentrations obtained from three independent biological experiments. Error bars: SD. P-values determined by unpaired two-tailed t-tests. Individual replicates are in S5 Fig.

In yeast and the protozoan parasite *Trypanosoma brucei*, inactivation of Vps15 results in a reduction of cellular PI3P levels [48,108,109]. To determine if this was the case with PfVps15, rapalog was added for 6 hours to trophozoites of the PfVps15mis and PfVps15ctrl lines after which lipids were extracted and PI3P quantified using a commercial ELISA kit. This revealed an 8-fold decrease in PI3P in the KS line compared to the control (PfVps15ctrl: 57.70 ± 11.83 pmol vs PfVps15mis: 7.20 ± 1.02 pmol. (Fig 8) directly demonstrating that the inactivation of PfVps15 impacts the biogenesis of PI3P. This therefore suggests that the lipid is likely directly implicated in the delivery of HCC to the FV and in apicoplast and mitochondrion dynamics. Although a role for PI3P in the first two processes correlates with the evidence previously obtained when incubating parasites with PI3K inhibitors [32], when inactivating PI3P binding proteins [31,33] or conditionally inactivating *PfPI3K* [34], its involvement in *P. falciparum* mitochondrion biology was unknown.

## Conclusion

**Fig 9:**
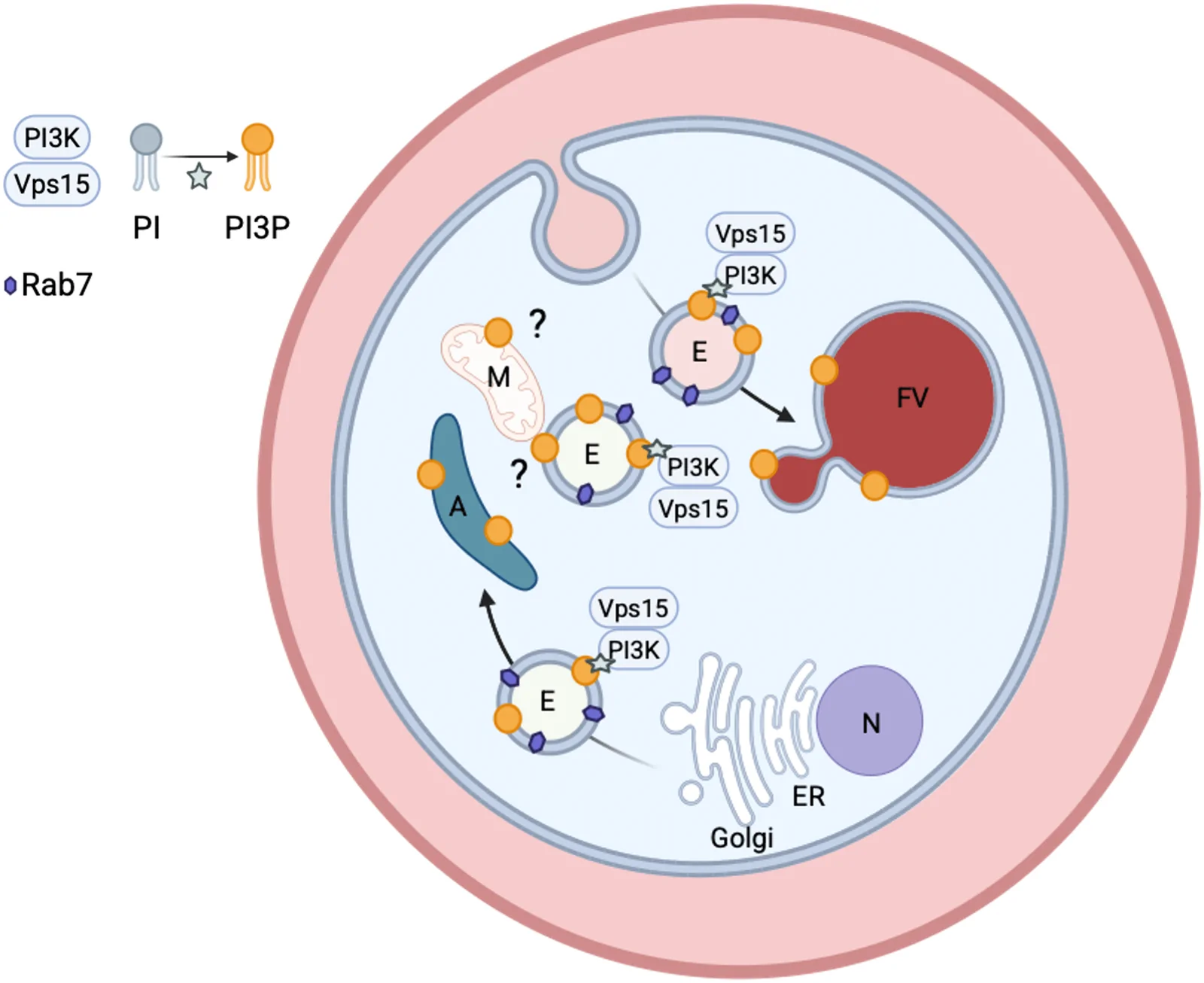
Working model for PfVps15-dependent PI3P generation and cargo trafficking in *Plasmodium falciparum.* Our colocalization analysis is consistent with the idea that a major pool of PI3P is generated at endosome-like structures that may serve as trafficking intermediates for cargo derived from endocytosis or from Golgi-directed delivery of apicoplast proteins. We propose that, during transit through these compartments, cargo could acquire PI3P and be sorted toward its destination organelle. For mitochondria, one possible route is via endosome–mitochondrion contact sites, analogous to mechanisms described in *C. elegans*. In addition, PI3P may also be generated locally at the apicoplast or directly at the cytostome, but these possibilities remain to be tested. FV: Food vacuole, E: Endosome-like structures with HCC (coloured red) or without (white); N: Nucleus; M: Mitochondrion; A: Apicoplast. Created in BioRender. Bourgeois, A. (2026) https://BioRender.com/m4egq75. Created in BioRender. Bourgeois, A. (2026) https://BioRender.com/p7vn7tv.

In conclusion, our work has shown that the *P. falciparum* orthologue of Vps15 is constitutively expressed during the asexual blood stages and that it interacts with PfPI3K. However, the potential lack of additional stable interactors suggests that, unlike model eukaryotic cells, PfVps15 and PfPI3K are not part of a heterotetrameric complex, raising the possibility of adaptations specific to *Plasmodium* parasites. Importantly, conditional inactivation of PfVps15 leads to a reduction in cellular PI3P levels, suggesting that this decrease might be responsible for the observed defects in the delivery of HCC-containing vesicles to the FV, and apicoplast and mitochondrial dynamics. Based on our finding that PfVps15 localizes more extensively with structures labelled with the endosomal marker PfRab7, we propose that these act as a platform for the synthesis of PI3P to which vesicular trafficking intermediates would transit, acquire the lipid and then be transported to their respective destination, whether the FV, the apicoplast or the mitochondrion (Fig 9). The presence of some PfVps15 at or proximal to these organelles could also mean that some PI3P is synthesized *in situ*. Further experiments will be required to determine how the trafficking intermediates would be differentially targeted from these endosome-like structures, but timing might be implicated since HCC trafficking, apicoplast development and mitochondrial fission mostly occur at different times during the asexual erythrocytic cycle.

## Materials and methods

### Ethics statement

Study approved by the Canadian Blood Services (CBS) research ethics board, project number 2023.030 and by the CHU de Québec IRB, project number 2015–2230, B14-12–2230, SIRUL 104595. Written consent was obtained by the CBS for all study participants. Participants were informed about the study before providing consent. All experiments were performed in accordance with relevant guidelines and regulations.

### Parasite culture

*Plasmodium falciparum* wild type 3D7 parasites (from David Walliker, Edinburgh University, Scotland) were cultured as previously described in [110]. Asexual stage parasites were cultured under standard conditions in RPMI-HEPES medium containing 4% hematocrit (human erythrocytes of O+ group) and 0.5% (w/v) Albumax (Invitrogen). All parasite cultures were kept at 37°C in a gas mixture composed of 5% oxygen, 5% carbon dioxide and 90% nitrogen.

### Vector construction

In order to endogenously tag PfVps15, we used the SLI system that was employed in [56]. To generate pSLI-PfVps15-2xFKBP-GFP vector, around 500 bp of the C-terminus of *PfVps15* was amplified with primers 5’NotI-3939-Vps15 (ATA**gcggccgc**TACAATTTATTCTTAACACTCAAC) and 3’AvrII-Stopless-Vps15 (ATACCACAAACAAAAATAATAAAAtgATATTTAtg**cctagg)** and cloned in frame with 2xFKBP-GFP in NotI - AvrII digested vector.

To generate a mNeonGreen tagged version of PfVps15, the GFP tag in the previously described SLI vector was replaced with the coding sequence for mNeonGreen. pSLI-PfVps15-2xFKBP-GFP was digested with MluI-SalI in order to remove GFP and mNeonGreen was amplified using the primers mNG FW (ATA**acgcgt**GTGAGCAAGGGCGAGGAGGATAAC) and mNG RV (ATA**gtcgac**CTTGTACAGCTCGTCCAtgC) from a vector containing this tag. mNeonGreen was then inserted in place of the GFP tag to achieve pSLI-PfVps15-2xFKBP-mNeonGreen.

In order to detect proper integration of the SLI vectors, the 5’ integration event was detected using the primer 5’intest3683-Vps15 (TATAAACAATTCAAtgAATAATAGACC) along with the primer FKBP (CAGAGCAGCTCTAGCAGC). The 3’ integration event was detected using the primer M13-Rev (CAGGAAACAGCTAtgAC) along with the primer 3’UTRintest (-31) Vps15 (TATATAAAtgTAtgTAACCATATA). Detection of the WT allele was done using the primer 5’intest3683-Vps15 (TATAAACAATTCAAtgAATAATAGACC) along with the primer 3’UTRintest (-31) Vps15 (TATATAAAtgTAtgTAACCATATA).

### Parasite transfection

Ring stage parasites were transfected and afterwards integrants were selected as previously described in [56] with some modifications. Briefly, to generate the two SLI lines, 100 μg of purified plasmid DNA (Promega) was prepared and transfected into *P. falciparum* 3D7 parasites. Transfected parasites were then positively selected initially using 2.5 nM WR99210 (WR, Jacobus Pharmaceuticals). Following initial selection, 1% parasitemia cultures were established in three wells and received 400 mg/ml neomycin (NEO, Wisent) as selection pressure for approximately 10 days to positively select for parasites having gained integration of the SLI plasmid. Integrated parasites were then cloned by limiting dilution to eliminate WT parasites.

Co-transfection was performed in a similar manner to generate the Knock Sideways parasite line. The PfVps15-2xFKBP-GFP line was transfected with 100 μg of purified 1xNLS-FRB-mCherry-DSM1 plasmid DNA (Promega) and selected with 1.5 μM DSMI (BEI Resources). Co-transfections were also performed in a similar manner to generated parasites lines needed for colocalizations. The PfVps15-2xFKBP-mNeonGreen line was transfected with 100 ug of purified pNMD3-PX40-mCherry-BSD, pHSP86-mScarlet-Linker-Rab7 and pHSP86-Atg18-Linker-mScarlet, all generated previously [31] and selected with either 1.5 μM DSMI or 2 μg/ml blasticidin (Sigma-Aldrich).

### Homology searching of PI3CK3 complexes components

To trace the conservation of components of PI3CK3 complexes and loss of WD40 repeat-like region in PfVps15, genome and protein datasets of fourteen species belonging to the SAR lineage were collected from publicly available sources (Source of data in S2 Data). Homology searches to identify Vps15, Vps38, Vps34, Atg6 and Atg14 orthologues were conducted using Analysis of Molecular Evolution with Batch Entry (AMOEBAE) [111]. *Homo sapiens* protein sequences were used as queries for initial homology searches with BLASTp and tBLASTn. Orthology is infirmed if the e-value of forward hit is below 0.05 and same query sequence is retrieved as top hit in reciprocal BLAST with e-value below 0.05 or e-value two orders of magnitude lower than non-redundant hit (S2 Table). Positive hits from initial searches were used to build Hidden Markov Models (HMMs) and conduct HMMER searches using AMOEBAE.

### Domain and structure analysis

Domain analysis of identified Vps15 orthologues was performed using InterProScan [112,113]. The presence of WD40 repeat-like region in *Toxoplasma gondii* was unclear from domain analysis alone. Therefore, the predicted tertiary structures of the WD40 repeat-like region in *Cryptosporidium muris* Vps15 and *Vitrella brassicaformis* Vps15 were compared to the predicted tertiary structures of the putative WD40 repeat-like region in *T. gondii* Vps15. Predicted tertiary structure of WD40 repeat-like regions of Vps15 were obtained in Protein data bank format (PDB) using Alphafold3 [114]. The putative WD40 repeat-like regions of Vps15 orthologues were defined based on domain analysis.

### Fluorescence imaging

Fluorescence imaging of live parasites was taken using a GE Healthcare Applied Precision Deltavision Elite Microscope with a 100X 1.4Na objective and a sCMOS camera. Following capture, images were deconvolved with the SoftWorx software. Prior to imaging, infected red blood cells were stained at 37°C for 10 minutes with 0.1 ug/ml of 4’, 6–diamidino-2-phenylindole (DAPI, Invitrogen). Images represent a single optical slice unless otherwise indicated.

### Western blotting

To determine the time course expression of PfVps15, the PfVps15-2xFKBP-GFP parasite line was synchronised by Percoll purification followed by 5% D-sorbitol treatment 4 hours later to obtain parasites aged 0–4 hours post invasion and cultures for each time point were established with an equal number of cells. Parasite cultures were purified by saponin lysis to release host cell cytosol at each time point and resuspended in cOmplete EDTA-free protease inhibitor (Roche) before being stored at -80°C until western blot. Parasite pellets were thawed and resuspended in SDS protein sample buffer before being separated on a 7% SDS-PAGE gel under reducing conditions. Following migration, proteins were transferred overnight at 4°C to PVDF membranes (Millipore). The membranes were blocked for 2 hours at RT in 10% Skim Milk in 0.1% (v/v) Tween 20-phosphate-buffered saline (TBS-T), followed by incubation overnight at 4°C with primary antibodies (anti-GFP (Roche) 1:500 or anti-aldolase (ICL) 1:2000) in 1% Skim Milk in TBS-T. Membranes were then incubated with the appropriate secondary horseradish peroxidase-coupled antibodies, washed and revealed by chemiluminescence (ECL, BioRad).

To determine proper expression of Vps15-2xFKBP-mNeonGreen by western blot, parasite cultures were purified by saponin lysis to release host cell cytosol and resuspended in cOmplete EDTA-free protease inhibitor (Roche) before being stored at -80°C until western blot. Western Blots were conducted as previously described with the use of anti-mNeonGreen (Cedarlane) (1:2500) primary antibodies and the appropriate secondary horseradish peroxidase-coupled antibody, with the exception of antibody incubation taking place at 37°C.

### Immunoprecipitation using anti-GFP agarose beads

Immunoprecipitation using anti-GFP beads was performed using three 30 ml cultures of both PfVps15-2xFKBP-GFP and wild type 3D7 parasites. Parasite cultures were synchronized twice by using 5% D-sorbitol treatments, as previously described in [110] at a 16-hour interval, afterwards parasitemia was adjusted to about 7% and parasites left to grow for 20 hours. Following this, parasite cultures were treated with saponin to release host cell cytosol and pellets were kept at -80 °C until immunoprecipitation. Thawed parasite pellets were resuspended in 1% T-Net containing cOmplete EDTA-free protease inhibitor (Roche) and incubated for 1 hour with agitation. Samples were then centrifuged for 10 minutes at 16 000 rpm in order to separate the insoluble and soluble protein fractions. Soluble protein fractions were then transferred to new microcentrifuge tubes containing the pre-washed anti-GFP mAb-agarose beads (MBL) and incubated 1 hour and 30 minutes with agitation. Beads were then spun down at 500 x g for 5 minutes and washed twice in 1X PBS before being resuspended in 100 ul of 1X PBS and kept at -80 °C until mass spectrometry analysis. Protein samples were incubated at 4 °C unless otherwise specified. Western Blot analysis was done using protein samples collected after each step during the immunoprecipitation. Briefly, samples were resuspended in SDS protein sample buffer before being separated on a 7% SDS-PAGE gel. Proteins were then transferred to a PVDF membrane (Millipore) overnight at 4°C. Membranes were blocked for two hours at room temperature in blocking solution (10% skim milk in 1X TBS-T), followed by incubation overnight with the primary antibody diluted in antibody solution (1% skim milk in 1X-TBS-T containing either rabbit anti-GFP (Roche) 1:500 or anti-Aldolase (ICL) 1:2000). Membranes were washed and incubated 1 hour with the corresponding secondary antibodies diluted in antibody solution. Membranes were washed before being revealed by chemiluminescence (ECL, BioRad). Anti-GFP mAb-agarose beads were then sent for mass spectrometry analysis.

### Sample preparation and data acquisition for mass spectrometry analysis

Protein digestion and mass spectrometry experiments were performed by the Proteomics platform of the CHU de Quebec Research Center, Quebec, Canada

### Protein digestion

On beads protein digestion was carried out using 0.1µg of modified porcine trypsin (sequencing grade, Promega, Madison, WI) in 50mM ammonium bicarbonate for 5 hours at 37°C. Digestion was stopped with 5% formic acid (FA) and peptides were eluted from the beads with 60% acetonitrile (ACN) 0.1% FA. Tryptic peptides were desalted on Stage tips (Empore C18, 3M Company), vacuum dried then resuspended in LC loading solvent (2% ACN, 0.05% trifluoroacetic acid (TFA)).

### Mass spectrometry

Half of each sample was analyzed by nanoLC/MSMS using a Dionex UltiMate 3000 nanoRSLC chromatography system (Thermo Fisher Scientific) connected to an Orbitrap Fusion mass spectrometer (Thermo Fisher Scientific, San Jose, CA, USA) equipped with a nanoelectrospray ion source. Peptides were trapped at 20 μl/min in loading solvent (2% ACN, 0.05% TFA) on a 5mm x 300 μm C18 pepmap cartridge (Thermo Fisher Scientific) during 5 minutes. Then, the pre-column was switched online with a 50 cm x 75µm internal diameter separation column (Pepmap Acclaim column, ThermoFisher) and the peptides were eluted with a linear gradient from 5-40% solvent B (A: 0.1% FA, B: 80% ACN, 0.1% FA) in 30 minutes, at 300 nL/min (60 minutes total runtime). Mass spectra were acquired using a data dependent acquisition mode using Thermo XCalibur software version 4.1.50. Full scan mass spectra (350 to 1800m/z) were acquired in the Orbitrap using an AGC target of 4e5, a maximum injection time of 50 ms and a resolution of 120 000. Internal calibration using lock mass on the m/z 445.12003 siloxane ion was used. Each MS scan was followed by acquisition of fragmentation MSMS spectra of the most intense ions for a total cycle time of 3 seconds (top speed mode). The selected ions were isolated using the quadrupole analyzer with 1.6 m/z windows and fragmented by Higher energy Collision-induced Dissociation (HCD) with 35% of collision energy. The resulting fragments were detected by the linear ion trap in rapid scan rate with an AGC target of 1e4 and a maximum injection time of 50ms. Dynamic exclusion of previously fragmented peptides was set for a period of 30 sec and a tolerance of 10 ppm.

### Database searching

MGF peak list files were created using Proteome Discoverer 2.3 software (Thermo). MGF files were then analyzed using Mascot (Matrix Science, London, UK; version 2.8.0). Mascot was set up to search a contaminant database and Uniprot *Plasmodium Falciparum* 3D7 (5538 entries, reference proteome UP000001450) database assuming the digestion enzyme trypsin. Mascot was searched with a fragment ion mass tolerance of 0.60 Da and a parent ion tolerance of 10.0 PPM. Carbamidomethyl of cysteine was specified in Mascot as a fixed modification. Deamidation of asparagine and glutamine and oxidation of methionine were specified in Mascot as variable modifications. 2 missed cleavages were allowed.

### Criteria for protein identification

Scaffold (version Scaffold_5.1, Proteome Software Inc., Portland, OR) was used to validate MS/MS based peptide and protein identifications. A false discovery rate of 1% was used for peptide and protein. Proteins that contained similar peptides and could not be differentiated based on MS/MS analysis alone were grouped to satisfy the principles of parsimony. Scaffold was also used to select proteins using a minimum of 2 peptides, with a peptide and protein threshold of 90%. Relevant proteins were found by calculating the normalized Log2 ratio to determine enrichment of proteins. Proteins were considered potential interaction partners if present in 2-fold or more in the Vps15 sample.

### Immunofluorescence assays

Immunofluorescence assays used for colocalization were performed using the pSLI-Vps15-2xFKBP-mNeonGreen parasite line. Parasite pellets were washed and then fixed using 4% paraformaldehyde and 0.01% glutaraldehyde in PBS for 30 minutes with gentle rocking. Fixed parasites were then washed twice in PBS, resuspended in 0.5 ml of PBS and transferred to a poly-L-lysine coated coverslip before being left to adhere for 30 minutes. Excess red blood cells were then removed and cells were permeabilized with 0.1% Triton X-100 (Sigma-Aldrich) for 10 minutes. Coverslips were washed once with washing buffer (0.05% PBS – Tween 20) and blocked for 1 hour with blocking buffer (3% BSA in 0.05% PBS – Tween 20). Following this, coverslips were incubated overnight at 4°C with rabbit anti-ERD2 (1:2000) [115], rabbit anti-ACP (1:200) [70], mouse anti-Rap1 (1:2000) [116] and rabbit anti-EBA175 (1:1000) [117]. Coverslips were washed with washing buffer three times and were incubated with appropriate secondary antibodies, either AlexaFluor-594 coupled anti-mouse (1:1000) or anti-rabbit (1:1000) for 1 hr at room temperature. Subsequently, coverslips were washed again three times with washing buffer and then fixed a second time with 4% paraformaldehyde and 0.1% glutaraldehyde in PBS for 5 minutes. Coverslips were washed a final time in MQ water and mounted in Vectashield (Vecta Laboratories) containing 0.1 mg/ml 4’, 6–diamidino-2-phenylindole (DAPI, Invitrogen). Images represent a single optical slice unless otherwise indicated. Pearson’s correlation coefficients were obtained using the ImageJ plugin JACoP [118]. Coefficients were determined without thresholding using a deconvolved single slice region of interest on IFA and live microscopy images. Statistical analysis was then conducted using Prism to determine statistical relevance with unpaired t-tests. Visual categorization (Overlap, partial overlap, proximal or none) of foci proximity was conducted on single slice regions of interest on IFA and live microscopy images.

Immunofluorescence assays to evaluate the apicoplast following KS were conducted like so. Briefly, PfVps15ctrl and PfVps15mis parasites were prepared in order to obtain tightly synchronized late schizont stage parasites. Percoll purification of schizonts followed by 5% D-sorbitol synchronization [110] 4 hours later. Parasites were allowed to grow for 24 hours, after which 150 nM A/C heterodimerizer (TaKaRa Bio) was added to culture media. Following another 24-hour incubation period, parasite cultures were sampled and used for IFAs as described above.

### Growth Assays of the knock sideways of PfVps15

Tightly synchronized PfVps15mis and PfVps15ctrl parasite lines were achieved by percoll purification of schizonts followed by reinvasion for 4 hours in fresh RBCs and then 5% D-sorbitol treatment [110] to obtain parasites aged 0–4 hours post invasion. Parasite cultures were then seeded at about 0.1% parasitemia and each received 150 nM A/C heterodimerizer (TaKaRa Bio). After 24, 72 and 120 hours in culture, parasites were sampled and analyzed by fluorescence-activated cell sorting (FACS) on a BD FACSCanto to evaluate parasitemia as described in [119]. Summarily, the parasites were stained with SYBRGold (Invitrogen-Molecular Probe), followed by fixation with 1% paraformaldehyde for 1 hour. A total of 100 000 events were recorded using the FACSDiva software and the results were analyzed using the FlowJo software. Uninfected red blood cells were used to determine the FITC signal threshold and the percentage of survival determined by normalizing to the PfVps15 parasite line which was considered 100% survival. GIEMSA-stained smears of parasite cultures were taken alongside each FACS reading time point to evaluate parasite maturation during the growth assay.

Parasitemia was also determined at each time point using GIEMSA-stained smears of the parasite cultures by counting 1000 RBCs for both PfVps15ctrl and PfVps15mis parasite lines. Different phenotypes observed following 150 nM A/C heterodimerizer (TaKaRa Bio) treatment starting in parasites aged 0–4 hours post invasion was also evaluated using 300 parasites.

PfVps15ctrl and PfVps15mis parasites were also prepared in order to obtain late ring stage parasites aged between 16 and 20 hours post invasion using 5% D-sorbitol synchronization [110]. In addition, PfVps15ctrl and PfVps15mis parasites were prepared in order to obtain trophozoite stage parasites aged between 26 and 30 hours post invasion using 5% D-sorbitol synchronization [110]. Following synchronisation, both of the PfVps15ctrl and PfVps15mis parasite lines were treated with 150 nM A/C heterodimerizer (TaKaRa Bio) and growth was followed over the next 8 hours using GIEMSA-stained smears of the parasite cultures. Parasitemia was also determined at each time point by counting 1000 RBCs and different phenotypes were also characterized and evaluated using 72–100 parasites.

### Vesicle accumulation assay

Vesicle accumulation assays were carried out as described in [120]. In short, PfVps15ctrl and PfVps15mis parasites were prepared to obtain trophozoite stage parasites aged between 28 and 34 hours post invasion by 5% D-sorbitol synchronization [110]. The two parasite cultures were each seeded into three 2 ml cultures and 150 nM A/C heterodimerizer (TaKaRa Bio) was added to all parasite cultures. Parasite pairs were sampled and visualized following a 4-, 6- and 8-hour incubation using a GE Healthcare Applied Precision Deltavision Elite Microscope. The accumulation of vesicles within the parasites at each time point were determined using DIC images analyzed using the FIJI software. The data represents a pool of three biological replicates. Statistics were determined by a two-tailed t test.

### Lysotracker experiments

Lysotracker imaging was performed as per manufacturer’s instruction following parasite synchronization. Briefly, PfVps15ctrl and PfVps15mis parasites were prepared in order to obtain trophozoite stage parasites aged between 28 and 34 hours post invasion using 5% D-sorbitol synchronization [110]. Following synchronisation both of the PfVps15ctrl and PfVps15mis parasite lines were treated with 150 nM A/C heterodimerizer (TaKaRa Bio) and allowed to grow for 6 hours. Labelling was then achieved by incubating parasite lines for 2 hours with 75 nM of Lysotracker Deep Red (Invitrogen) at 37°C. Following staining, parasites were imaged immediately. Images represent a single optical slice unless otherwise specified. Quantification of lysotracker stained vesicle-like compartments within the parasites was determined using the red channel of the fluorescence microscopy images using the FIJI software. Statistics were determined by unpaired t-test.

### Anti-hemoglobin IFA

As in [120], anti-hemoglobin IFAs were carried out using 10 ml cultures of 5–10% parasitemia of PfVps15ctrl and PfVps15mis parasites. 150 nM of A/C heterodimerizer (TaKaRa Bio) was added to both cultures and parasites were incubated for 6 hours. Parasite cultures were then centrifuged for 5 minutes at 1200 rpm, after which they were resuspended in 5 ml of a 0.03% saponin solution in 1X PBS and incubated on ice for 15 minutes. Parasites liberated from the host cell were then washed 3 times in 1X PBS. Parasites were then fixed in fixing solution (1X PBS with 4% paraformaldehyde and 0.005% glutaraldehyde) for 30 minutes. Following this, the parasites were then washed 3 times in 1X PBS and blocked and permeabilized for 1 hour at room temperature in blocking solution (1X PBS with 3% bovine serum albumin (BSA, Wisent), 0.1% saponin and 100 ug/ml ampicilin). After blocking, parasites were incubated at 4°C overnight with rotation in blocking solution supplemented with the primary antibody (anti-hemoglobin 1: 1000 (Cedarlane)). Subsequently, parasites were washed 3 times in 1X PBS before incubation for 1 hour at room temperature with rotation in blocking solution supplemented with the secondary antibody (AlexaFluor 594-conjugated anti-goat 1:500 (Cedarlane)). Parasites were washed 3 times with 1XPBS before being seeded onto a glass slide with VECTASHIELD antifade mounting medium with DAPI (VectorLabs), covered with a coverslip and imaged. Between 65 and 89 images were analysed using the FIJI software and hemoglobin-filled vesicles were enumerated.

### Anti-hemoglobin western blot

In order to evaluate intracellular quantities of accumulated Hb within the parasites, a Western blot was performed. PfVps15ctrl and PfVps15mis parasite were prepared in order to obtain trophozoite stage parasites aged between 28 and 34 hours post invasion using 5% D-sorbitol synchronization [110]. Following synchronisation, the parasitemia of both of the PfVps15ctrl and PfVps15mis parasite lines were determined and parasite cultures were established with an equal number of cells. Parasites were then treated with 150 nM A/C heterodimerizer (TaKaRa Bio) and allowed to grow for 8 hours. Parasite cultures were purified twice by saponin lysis in order to release all host cell cytosol at each and resuspended in cOmplete EDTA-free protease inhibitor (Roche) before being stored at -80°C until western blot. Parasite pellets were thawed and resuspended in SDS protein sample buffer before being separated on a 7% SDS-PAGE gel under reducing conditions. Following migration, proteins were transferred overnight at 4°C to PVDF membranes (Millipore). The membranes were blocked for 2 hours at RT in 10% Skim Milk in 0.1% (v/v) Tween 20-phosphate-buffered saline (TBS-T), followed by incubation overnight at 4°C with primary antibodies (anti-hemoglobin (Cerdarlane) 1:2000 or anti-HSP70 (StressMarq) 1:5000) in 1% Skim Milk in TBS-T. Membranes were then incubated with the appropriate secondary horseradish peroxidase-coupled antibodies, washed and revealed by chemiluminescence (ECL, BioRad).

### Heme fractionation assay

Heme fractionation was performed from adapted protocols [121–123]. PfVps15ctrl and PfVps15mis parasite lines were tightly synchronized by Percoll purification of late schizonts and 5% D-sorbitol treatment [110] 4 hours later. Following synchronization, parasites were allowed to grow for 4 hours and then passed through a magnetized LS Column (Miltenyi) to effectively remove dead parasites and hemozoin coming from ruptured schizonts following 5% D-sorbitol treatment. Parasitemia was then determined for PfVps15ctrl and PfVps15mis parasite lines and cultures established with equal number of cells and let to grow until the trophozoite stage. Parasite lines then received 150 nM A/C heterodimerizer (TaKaRa Bio) treatment for 8 hours. Following this, triplicate samples of PfVps15ctrl and PfVps15mis 100 x 10^6^ iRBCs were subject to saponin lysis with 0.1% saponin in PBS with added cOmplete EDTA-free protease inhibitor (Roche). Saponized samples were washed three times with cold PBS and kept at -80°C until heme fractionation. In order to obtain the Hb fraction, parasite saponin pellets were resuspended in 50 mL of MQ water and underwent sonication for 5 minutes in a water bath sonicator. 50 mL of 0.2M HEPES (pH 7.5) was added to the sonicated cell suspensions before centrifugation at 1500 x g for 20 minutes at 4°C. Following centrifugation, the supernatant containing the cytosolic Hb fraction was transferred to new tubes. 50 mL of 4% of SDS was added and the samples were incubated for 5 minutes at 95°C. Subsequently, 50 mL of 0.3M NaCl and 50 mL of 25% (v/v) pyridine (Sigma) in 0.2M HEPES was added and the samples were vortexed before being transferred to a 96-well plate. Absorbance at 405 nm was read immediately to determine concentration of cytosolic heme fraction (corresponding to Hb). The remaining pellets were once again resuspended in 50 mL of MQ and 50 mL of 4% of SDS. Resuspended samples were then sonicated for 5 minutes in a water bath sonicator. Following sonication, to solubilize FV-associated heme (in the form of Hb, free heme and Hz), samples were incubated at 95°C for 5 minutes. After the heat treatment, 50 mL of 0.2M HEPES, 50 mL of 0.3M NaCl and 50 mL of 25% (v/v) pyridine were supplemented to the samples, which were then centrifuged at 1500 x g for 20 min. The supernatants containing the free heme/FV-associated Hb were then transferred to a 96-well plate. The absorbance at 405 nm was then read immediately. Finally, the remaining pellet containing the Hz fraction was resuspended in 50 mL of MQ water and 50 mL of 0.3M NaOH and then vortexed for 10 seconds. Vortexed samples were then subject to a third round of sonication for 15 minutes, after which 50 mL of 0.2M HEPES, 50 mL of 0.3M HCl and 50 mL of 25% (v/v) pyridine was added. The full samples containing the final Hz fraction were then transferred to a 96-well plate. The quantification of heme Fe was achieved by comparing to a standard curve determined using hematin (porcine, Sigma) solution that was dissolved in 0.3M NaOH. The series dilutions of hematin were made in a 96-well plate in which necessary components to form the heme-pyridine complex were added (including 0.2M HEPES pH 7.5, 4% (w/v) SDS, 0.3M NaCl, O.3M HCL, 25% pyridine in 0.2M HEPES pH 7.5 and MQ water). Absorbance was read at 405 nm. The concentration of heme for every fraction was then calculated by comparing the OD_450nm_ to the standard curve established. This was the divided by the total number of cells used in the assay. Statistics were determined by a two-tailed t test.

### Host-cell cytosol uptake assay

Host cell cytosol uptake assays were adapted from [120,124]. For this, red blood cells were preloaded with Alexa Fluor–647 conjugated dextran (Thermo Fisher). 32 ul of packed red blood cells were washed three times in cold PBS and then carefully added to freshly prepared red blood cell preloading lysis buffer (64 μl of 5 mM K_2_HPO_4_/20 mM D-Glucose pH 7.4, 1 μl of 30 mM DTT, 2 ml of 50 mM MgATP and 1 μl (50 mg/mL) of Alexa Fluor-647 - conjugate 10 kDa dextran (Thermo Fisher)). The reb blood cells were then subject to a 10-minute incubation at 4°C with rotation. Following this, red blood cells were resealed by slowly adding 25 ml of the 5X preloading resealing buffer (750 mM NaCl/ 25 mM Na2HPO4 pH 7.4) into the lysis mixture with the red blood cell. This red blood cell suspension was then incubated with gentle rocking at 37°C for one hour. The resulting pre-loaded cells were then washed three times with RPMI and kept at 4°C until host cell cytosol uptake assay. The PfVps15ctrl and PfVps15mis parasite lines were prepared by Percoll purification of late stage schizonts. The resulting schizonts were then seeded into labelled dextran pre-loaded red blood cells to allow for reinvasion. Parasites were then allowed to grow until the trophozoite stage, after which samples were taken and imaged by fluorescence microscopy. Images represent single optical slice unless otherwise specified. Quantification of Alexa Fluor-647-labelled dextran-stained HCC-uptake intermediates within the parasites was determined using the red channel of the fluorescence microscopy images using the FIJI software.

### Bloated food vacuole assay

Bloated food vacuole assays were performed as described in [120] with modifications. PfVps15ctrl control and PfVps15mis parasite strains were synchronized once with 5% D-sorbitol treatment [110] and incubated for 16 hours to obtain trophozoite stage parasites aged between 16 and 34 hours post invasion. Parasite cultures were seeded in 2 ml cultures and each received 150 nM of A/C heterodimerizer (TaKaRa Bio) and 33 μM of E64. Parasite cultures were incubated for 0, 2 or 4 hours before staining cells with 4.5 ug/ml of DHE (Cayman Chemical Company) for 20 minutes at room temperature. Following staining, the parasites were washed once with RPMI and imaged immediately. For each condition, between 30 and 40 parasites were imaged and the DIC with superimposed DHE images were used once again to measure the area of the parasites and the area of the food vacuoles using the FIJI software.

### Organellar to nuclear genome ratios by quantitative real-time PCR

Apicoplast to nuclear genome ratios were determined by quantitative real time PCR as in [125]. Briefly, parasites were synchronized PfVps15mis parasite were prepared in order to obtain trophozoite stage parasites aged between 28 and 34 hours post invasion using 5% D-sorbitol synchronization [110]. Following synchronization, the parasitemia of both of the PfVps15ctrl and PfVps15mis parasite lines were determined, and parasite cultures were established with an equal number of cells and left to grow until late trophozoite stage. Parasites were then treated with 150 nM A/C heterodimerizer (TaKaRa Bio) and allowed to grow for 6 hours. Parasite samples were then collected by saponin lysis and genomic DNA was purified. Primers used were: cht1 (nuclear) TCCATTGGTGATTTTGTAAAGACTG (forward) and cht1 (nuclear) CTAATTGTTCATTATGTGCAGCATTATC (reverse), tufa (apicoplast) AATTAACACAAGCACAATCCGG (forward) and tufa (apicoplast) GGTTTATGACGACCACCTTCT (reverse), and cytb3 (mitochondrion) CTGCTTTCGTTGGTTATGTCTTAC (forward) and cytb3 (mitochondrion) CTCACAGTATATCCTCCACATATCC (reverse). Reactions were set up as follows: Template DNA, 0.5 μM of each primer pair, and SssoAdvanced Universal SYBR Green Supermix (BIORAD). Triplicates were performed for all quantitative real-time PCR rections using a 2-step reaction with 95oC denaturation and 56oC annealing and extension for 35 cycles on a Rotor-Gene Q (QUIAGEN). The relative quantification of each target gene was then determined using the Rotor-Gene Q software 2.3.5.1. Primer efficiency was verified by series dilution of PfVps15ctrl gDNA ranging from 20 to 0.2 ng. The organelle (apicoplast or mitochondrion) to nuclear genome ratio was determined for the PfVps15mis parasite line using the PfVps15ctrl parasite line as a control.

### Apicoplast resident protein processing Western Blot

PfVps15ctrl and PfVps15mis parasites were prepared in order to obtain trophozoite stage parasites aged between 28 and 34 hours post invasion using 5% D-sorbitol synchronization [110]. Following synchronization, the parasitemia of both of the PfVps15ctrl and PfVps15mis parasite lines were determined and parasite cultures were established with an equal number of cells and left to grow until late trophozoite stage. Parasites were then treated with 150 nM A/C heterodimerizer (TaKaRa Bio) and allowed to grow for 6 hours. Parasite cultures were purified by saponin lysis to release all host cell cytosol at each and resuspended in cOmplete EDTA-free protease inhibitor (Roche) before being stored at -80°C until western blot. Parasite pellets were thawed and resuspended in SDS protein sample buffer before being separated on a 7% SDS-PAGE gel under reducing conditions. Following migration, proteins were transferred for 1 hour at 4°C to PVDF membranes (Millipore). The membranes were blocked for 2 hours at RT in 10% Skim Milk in 0.1% (v/v) Tween 20-phosphate-buffered saline (TBS-T), followed by incubation for 1 hour at room temperature with primary antibodies (anti-PfClpR 1:2000 [88] or anti-HSP70 (StressMarq) 1:5000) in 1% Skim Milk in TBS-T. Membranes were then incubated with the appropriate secondary horseradish peroxidase-coupled antibodies, washed and revealed by chemiluminescence (ECL, BioRad).

### Mitotracker experiments

Mitotracker imaging was performed as per manufacturer’s instruction following parasite synchronization. Briefly, PfVps15ctrl and PfVps15mis parasites were prepared in order to obtain trophozoite stage parasites aged between 28 and 34 hours post invasion using 5% D-sorbitol synchronization [110]. Following synchronization both of the PfVps15ctrl and PfVps15mis parasite lines were left to grow until reaching schizont stage and treated with 150 nM A/C heterodimerizer (TaKaRa Bio) and allowed to grow for 6 hours. Labelling was then achieved by incubating parasite lines for 10 minutes with 100 nM of Mitotracker Red CMXROS (Invitrogen) at 37°C. Following staining, parasites were imaged immediately. Images represent a single optical slice unless otherwise specified. Characterization of mitochondrial stage was determined using the red channel of the fluorescence microscopy images using the FIJI software. Pearson’s correlation coefficients were obtained using the ImageJ plugin JACoP [118]. Coefficients were determined without thresholding using a deconvolved single slice region of interest on live microscopy images of parasites stained with mitotracker. Statistical analysis was then conducted using Prism to determine statistical relevance with unpaired t-tests. Visual categorization (Overlap, partial overlap, proximal or none) of foci proximity was conducted on single slice regions of interest on live microscopy images.

### PI3P ELISA

Tightly synchronized PfVps15ctrl control and PfVps15mis parasite lines were achieved by percoll purification of schizonts followed by reinvasion for 4 hours in fresh RBCs and then 5% D-sorbitol treatment to obtain parasites aged 0–4 hours post invasion. Both PfVps15ctrl and PfVps15mis parasite lines received 150 nM of A/C heterodimerizer (TaKaRa Bio) at 26–30 hpi to induce KS in trophozoites. Parasitemia of all parasite lines was then determined and parasites were harvested at 32–38 hpi by saponin lysis to release host cell cytosol. The detection of PI3P was done with 6x10^7^ parasites per condition using a PI3P mass ELISA kit (Echelon Biosciences). Parasite samples were processed as per the manufacturer’s instructions in order to extract lipids and quantify PI3P concentrations.

## Supporting information

S1 Table Data from Immunoprecipitation of PfVps15-2xFKBP-GFP and 3D7 control. (XLSX)

S2 Table List of Vps15, Vps38, Vps34, ATG6 and ATG14 homologs identified across representatives from SAR lineages. (XLSX)

S3 Table Vps15 domain conservation in SAR lineages. (XLSX)

S1 Fig Construction and validation of the PfVps15-2xFKBP-GFP and PfVps15-2xFKBP-mNeonGreen parasite strains. A. Illustration of the selection-linked integration (SLI) strategy to generate PfVps15-2xFKBP-GFP. Cter: C-terminus of *PfVps15*; 2A: T2A skip peptide; NeoR: neomycin resistance gene; arrows: PCR primers to detect integration. Image not to scale. B. PCR on genomic DNA from one clone of the PfVps15-2xFKBP-GFP parasite line demonstrating proper genomic integration of the vector and the absence of the WT allele following SLI. (5’: P1, P3; 3’: P4, P2; WT: P1, P2). Expected size of PCR fragments are as follows: 5’ 1151 bp, 3’ 652 bp and WT 795 bp. C. Time course of PfVps15-2xFKBP-GFP expression by anti-GFP Western Blot showing expression of the fusion protein during ring, trophozoite and schizont stages. Anti-Aldolase is used as a constitutive loading control at each time point. D. Endogenous expression of PfVps15-2xFKBP-GFP during the asexual parasite stages. DIC: Differential interference contrast. Blue: DAPI-stained nuclei; Merge: merged green and DAPI channels with DIC. Scale bar: 5 μm. E. Illustration of the selection-linked integration (SLI) strategy used to create PfVps15-2xKBP-mNeonGreen. Cter: C-terminus of *PfVps15*; NG: mNeonGreen tag; 2A: T2A skip peptide; NeoR: neomycin resistance gene; arrows: PCR primers to detect integration. Image not to scale. F. PCR on genomic DNA from one clone of the PfVps15-2xFKBP-mNeonGreen parasite line demonstrating proper genomic integration of the vector and the absence of the WT allele following SLI. (5’: P1, P3; 3’: P4, P2; WT: P1, P2). Expected size of PCR fragments are as follows: 5’ 1151 bp, 3’ 652 bp and WT 795 bp. G. Western blot on mixed parasite stages showing the proper expression of PfVps15-2xFKBP-mNeonGreen.
(PDF)

S2 Fig Western Blot of the immunoprecipitation of PfVps15-2xFKBP-GFP (PfVps15) using anti-GFP agarose beads. One representative of two independent experiments is shown.
(PDF)

S3 Fig Immunofluorescence assays. Colocalization analysis of PfVps15-2xFKBP-mNeonGreen with: A. PfErd2, a marker of the Golgi apparatus. B. PfRAP1, a marker of the rhoptries. C. PfEBA175, a marker of micronemes. Blue: DAPI stained nuclei. Merge: merged green, magenta and DAPI channels. Scale bar: 5 μm.
(PDF)

S4 Fig Growth curves of the two biological replicates from Fig 3Aiii. Both control and mislocalizer lines were incubated with rapalog. Control: PfVps15-2xFKBP. Mislocalizer: PfVps15-2xFKBP-GFP+mislocalizer. Error bars: SD of three technical replicates.
(PDF)

S5 Fig Individual bioreplicates of the PI3P ELISA in Fig 7. Quantification of cellular PI3P levels by PI3P Mass ELISA in trophozoite stage of PfVps15mis and PfVps15ctrl parasites after a 6-hour rapalog treatment. Graphs represent PI3P concentrations obtained from three independent biological experiments. Error bars: SD.
(PDF)

S1 Data File containing the raw data for Figs 2–8. (XLSX)

S2 Data File containing the links for the data used in the evolutionary analysis of Fig 1. (XLSX)

S1 Raw Gel Uncropped blots and gels. (PDF)
