# Supplementary material for: The malaria parasite Plasmodium falciparum Vps15 is a Phosphatidylinositol 3-kinase interactor important for host-cell cytosol delivery to the food vacuole and apicoplast and mitochondrion dynamics
Source: PLoS Pathog. 2026 Aug 18;22(8):e1014526. doi: 10.1371/journal.ppat.1014526 (PMC13497265; doi:10.1371/journal.ppat.1014526)
Supplement: Supplementary file 1 [file ppat.1014526.s004.pdf]

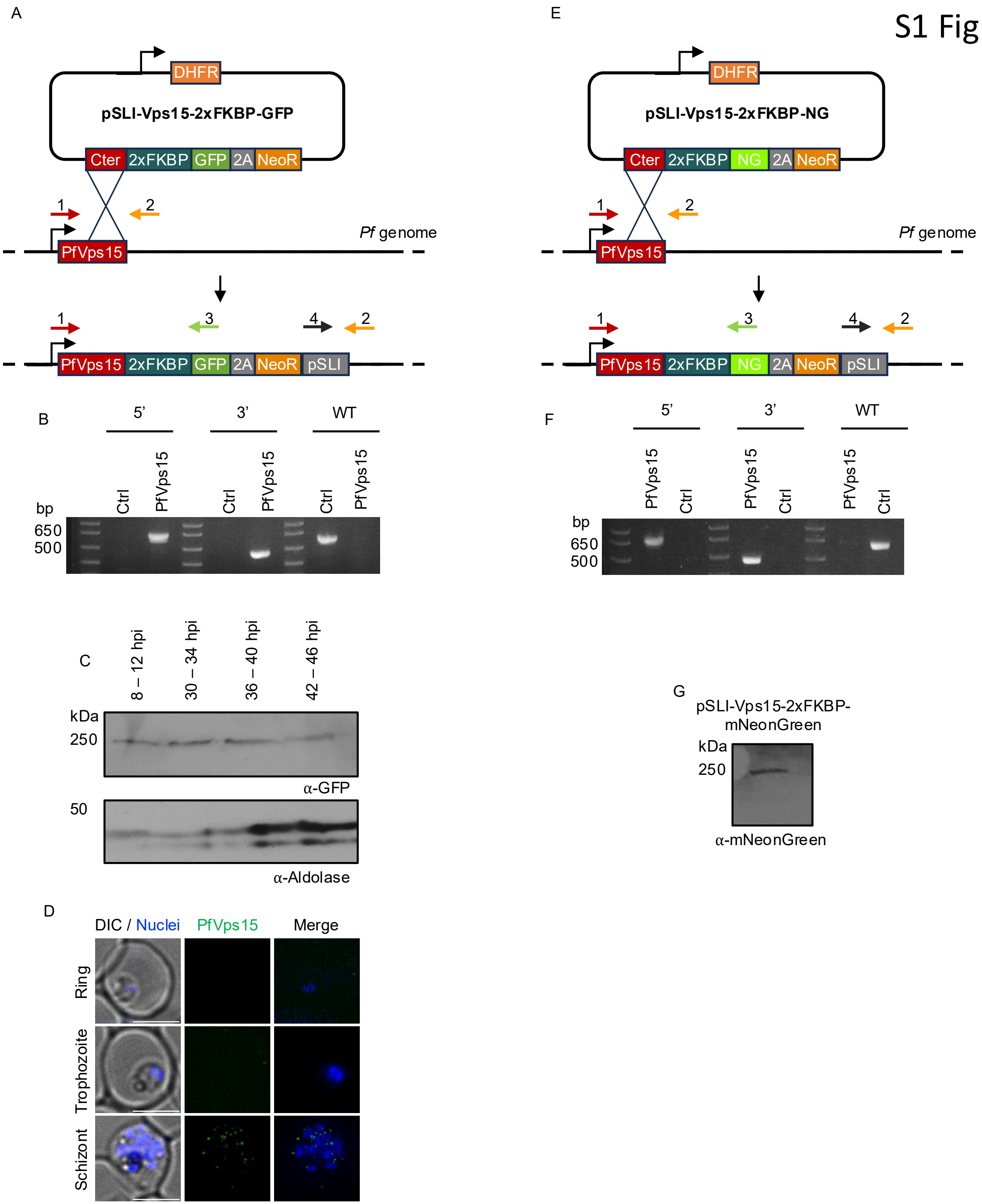

S1 Fig
A
DHFR
pSLI-Vps15-2xFKBP-GFP
Cter
2xFKBP
GFP
2A
NeoR
1
2
Pf genome
PfVps15
1
3
4
2
PfVps15
2xFKBP
GFP
2A
NeoR
pSLI
B
5'
3'
WT
Ctrl
PfVps15
Ctrl
PfVps15
Ctrl
PfVps15
bp
650
500
C
8 – 12 hpi
30 – 34 hpi
36 – 40 hpi
42 – 46 hpi
kDa
250
α-GFP
50
α-Aldolase
D
DIC / Nuclei
PfVps15
Merge
Ring
Trophozoite
Schizont
E
DHFR
pSLI-Vps15-2xFKBP-NG
Cter
2xFKBP
NG
2A
NeoR
1
2
Pf genome
PfVps15
1
3
4
2
PfVps15
2xFKBP
NG
2A
NeoR
pSLI
F
5'
3'
WT
PfVps15
Ctrl
PfVps15
Ctrl
PfVps15
Ctrl
bp
650
500
G
pSLI-Vps15-2xFKBP-mNeonGreen
kDa
250
α-mNeonGreen
