# Supplementary material for: The malaria parasite Plasmodium falciparum Vps15 is a Phosphatidylinositol 3-kinase interactor important for host-cell cytosol delivery to the food vacuole and apicoplast and mitochondrion dynamics
Source: PLoS Pathog. 2026 Aug 18;22(8):e1014526. doi: 10.1371/journal.ppat.1014526 (PMC13497265; doi:10.1371/journal.ppat.1014526)
Supplement: Supplementary file 4 [file ppat.1014526.s007.pdf]

S4 Fig

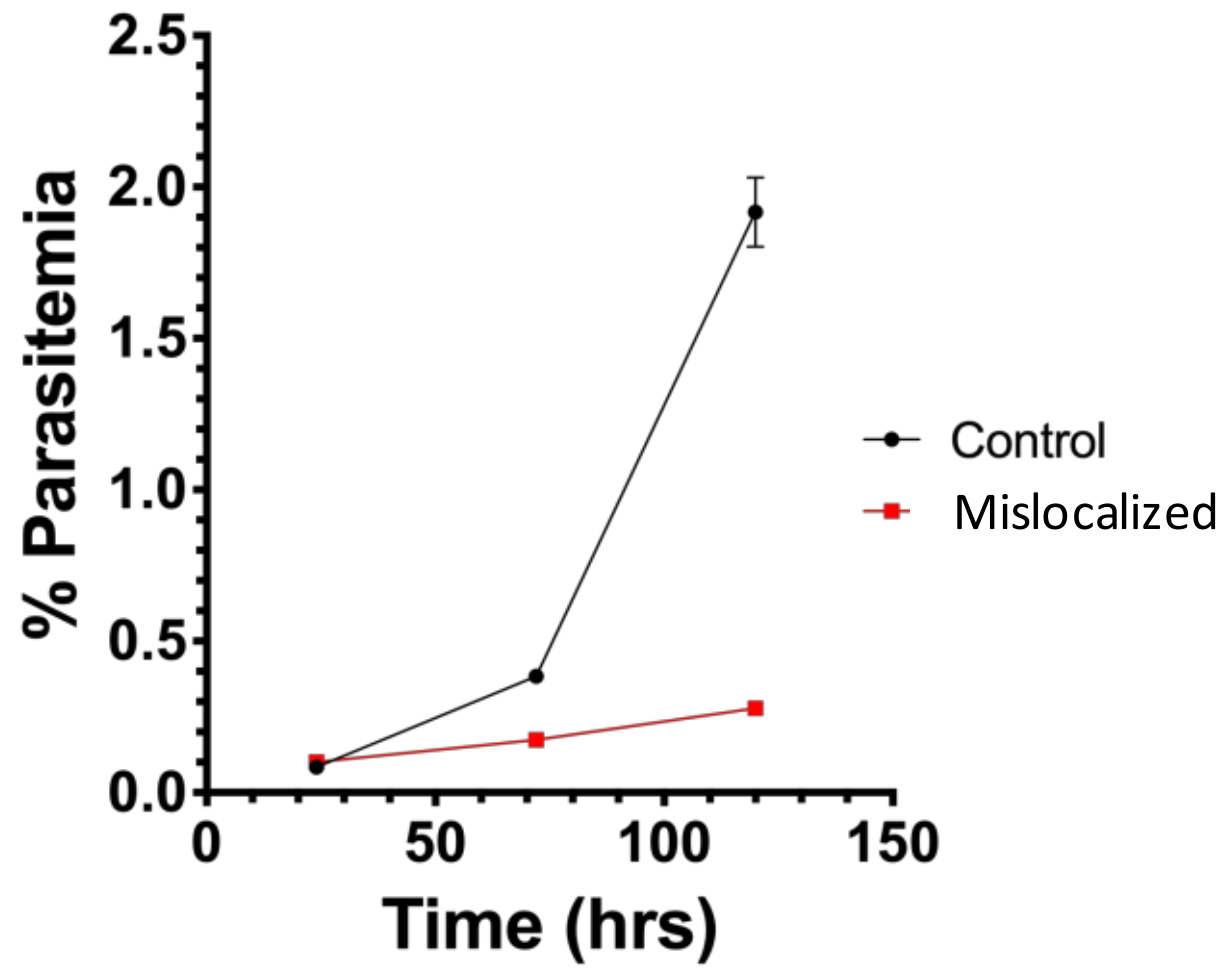
2.5
2.0
1.5
1.0
0.5
0.0
% Parasitemia
0
50
100
150
Time (hrs)
Control
Mislocalized

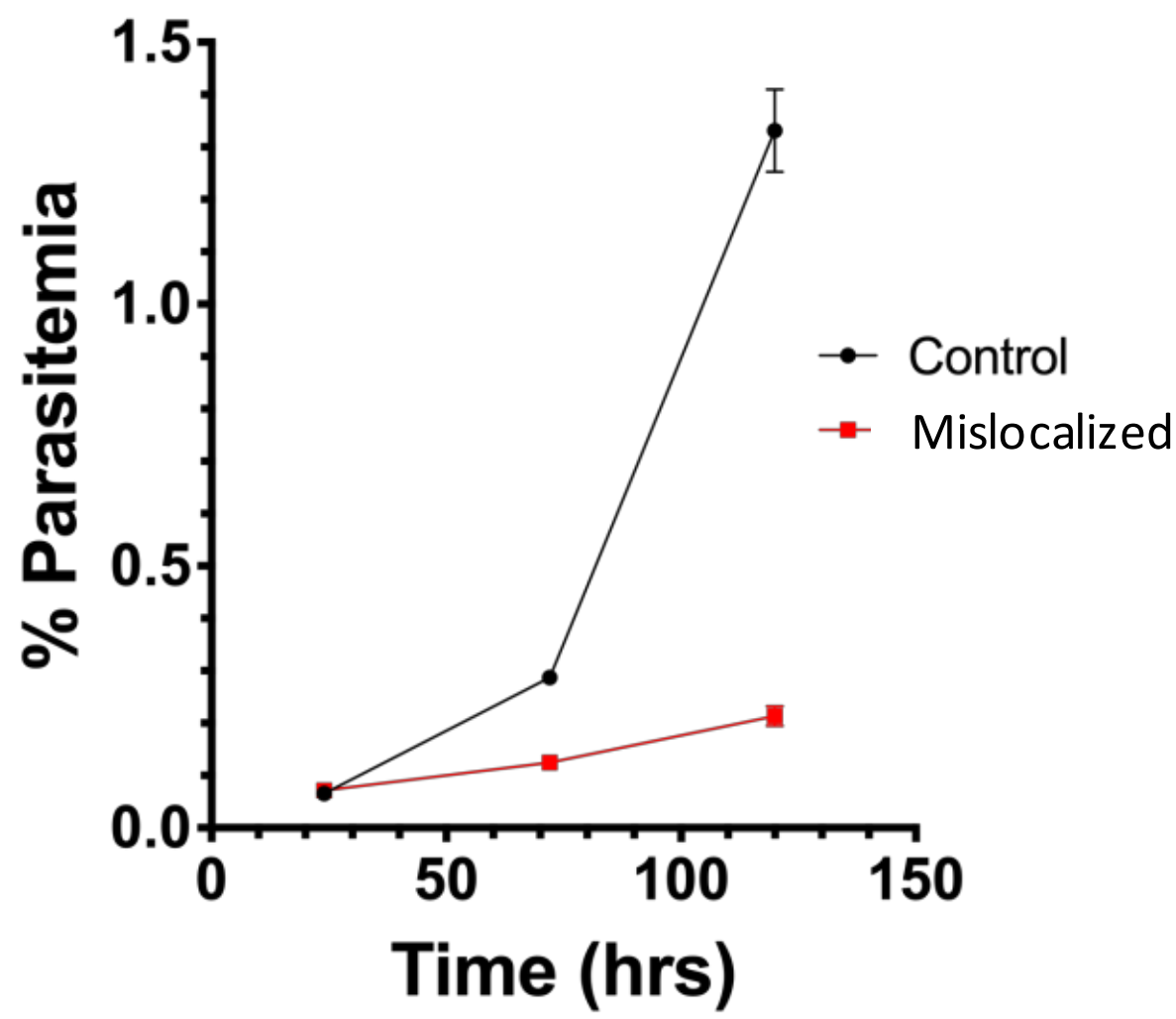
1.5
1.0
0.5
0.0
% Parasitemia
0
50
100
150
Time (hrs)
Control
Mislocalized
