# Supplementary material for: The malaria parasite Plasmodium falciparum Vps15 is a Phosphatidylinositol 3-kinase interactor important for host-cell cytosol delivery to the food vacuole and apicoplast and mitochondrion dynamics
Source: PLoS Pathog. 2026 Aug 18;22(8):e1014526. doi: 10.1371/journal.ppat.1014526 (PMC13497265; doi:10.1371/journal.ppat.1014526)

S5 Fig

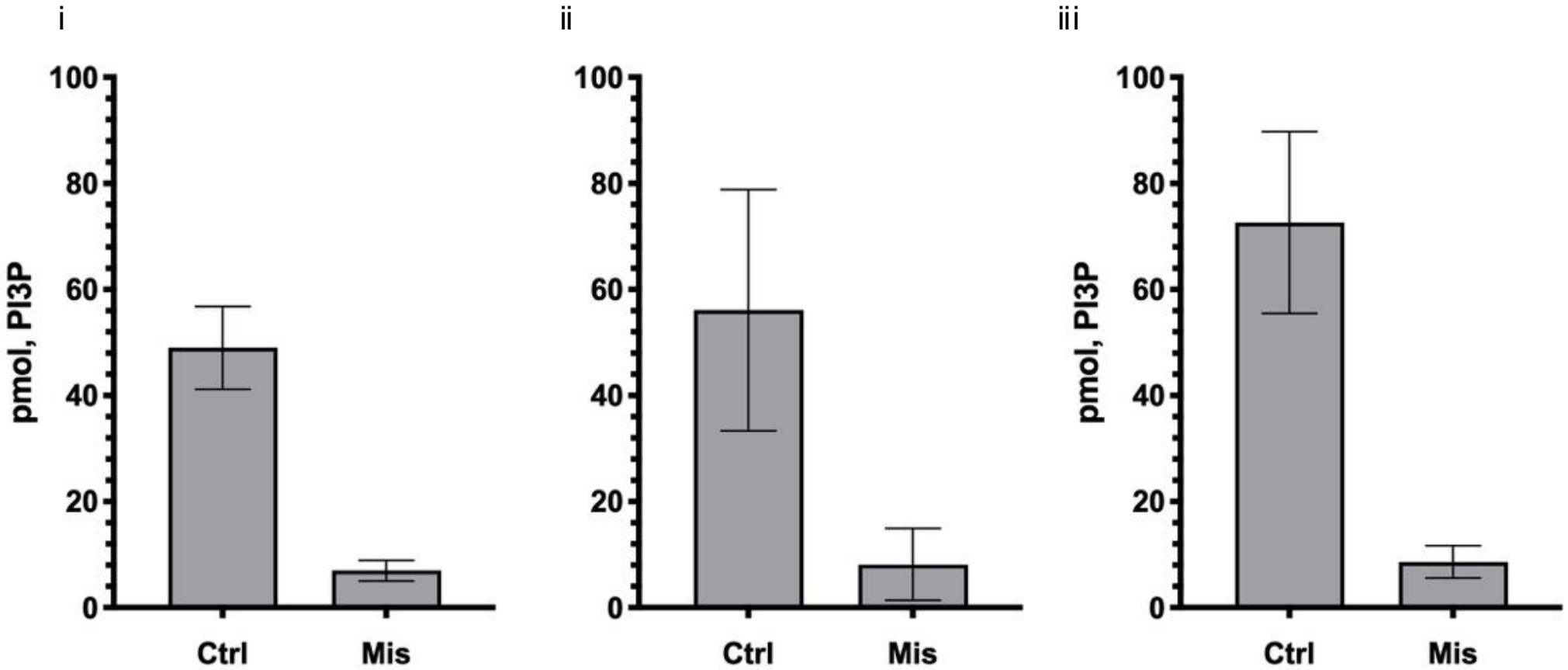

Supplement: Supplementary file 5 [file ppat.1014526.s008.pdf]
