# Supplementary material for: The malaria parasite Plasmodium falciparum Vps15 is a Phosphatidylinositol 3-kinase interactor important for host-cell cytosol delivery to the food vacuole and apicoplast and mitochondrion dynamics
Source: PLoS Pathog. 2026 Aug 18;22(8):e1014526. doi: 10.1371/journal.ppat.1014526 (PMC13497265; doi:10.1371/journal.ppat.1014526)
Supplement: Supplementary file 6 [file ppat.1014526.s011.pdf]

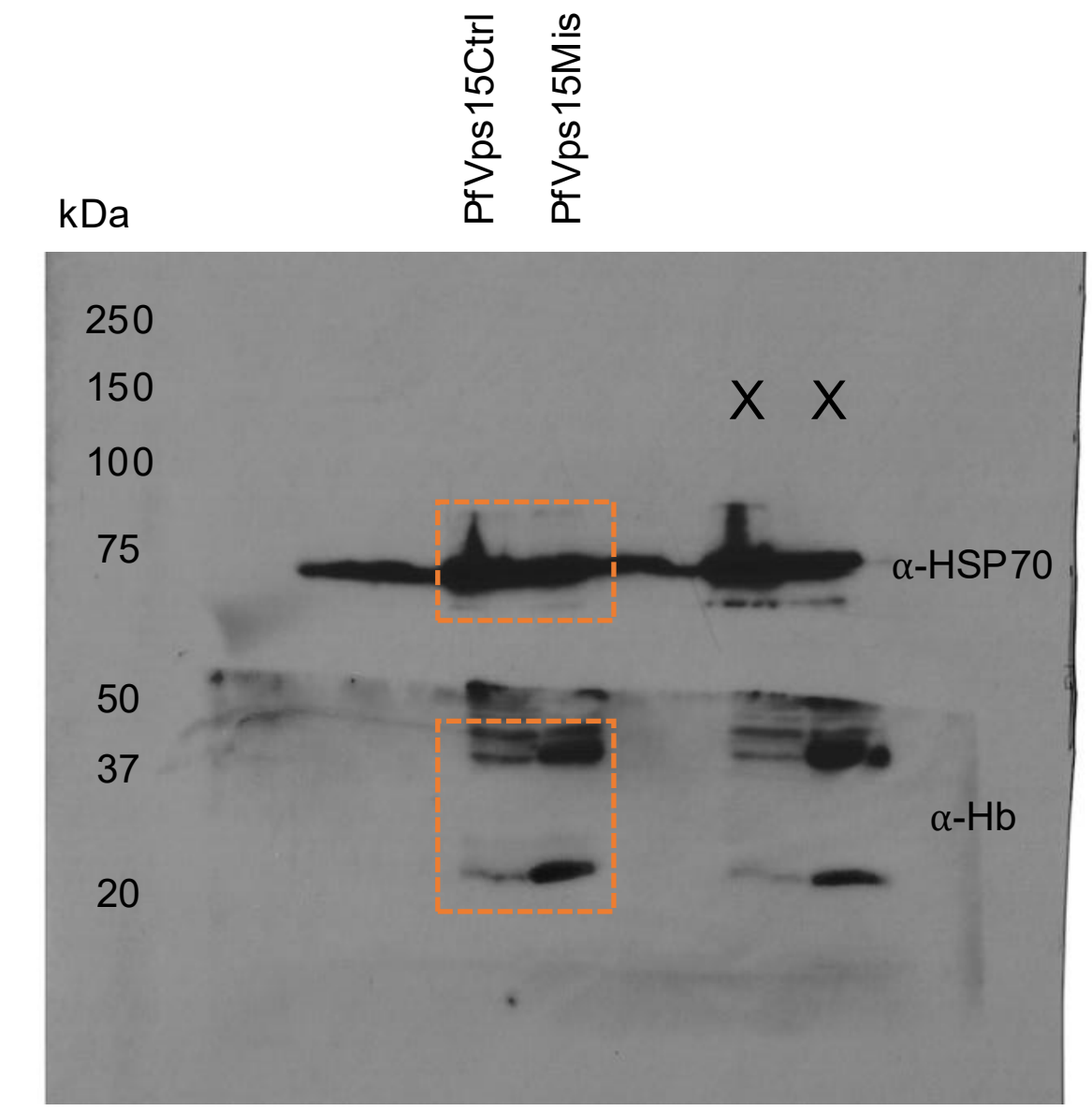

Related to Fig 4 F

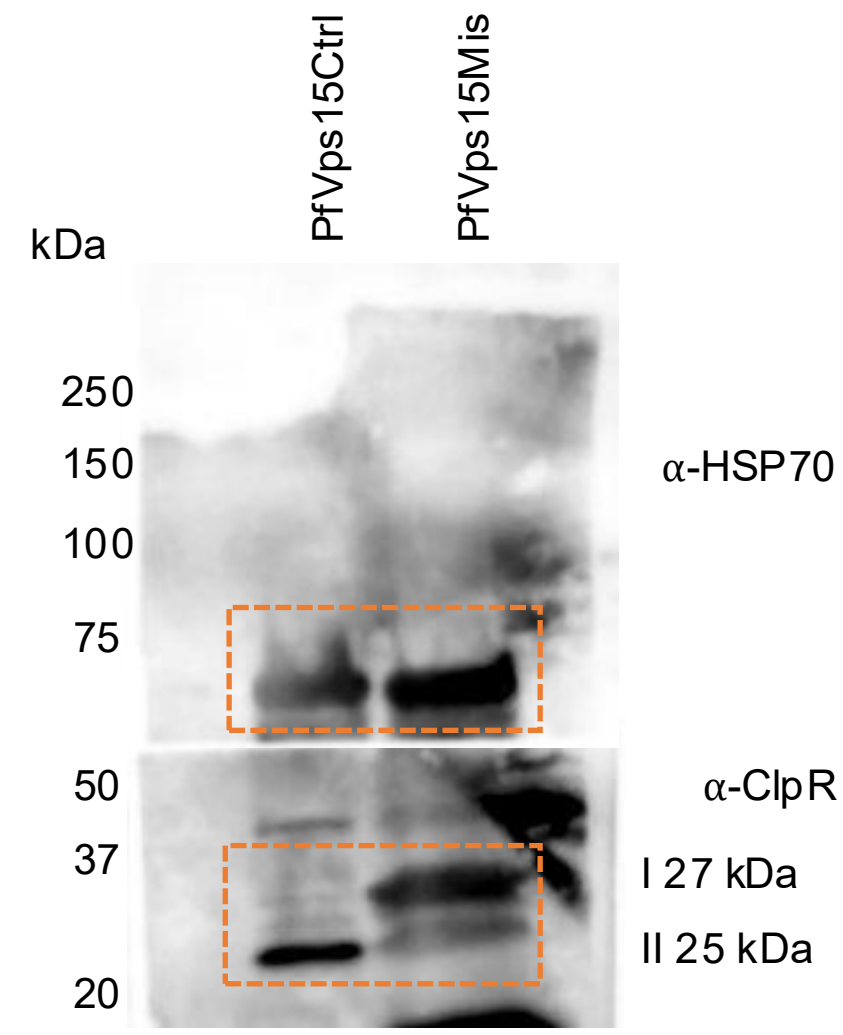

Related to Fig 6 E

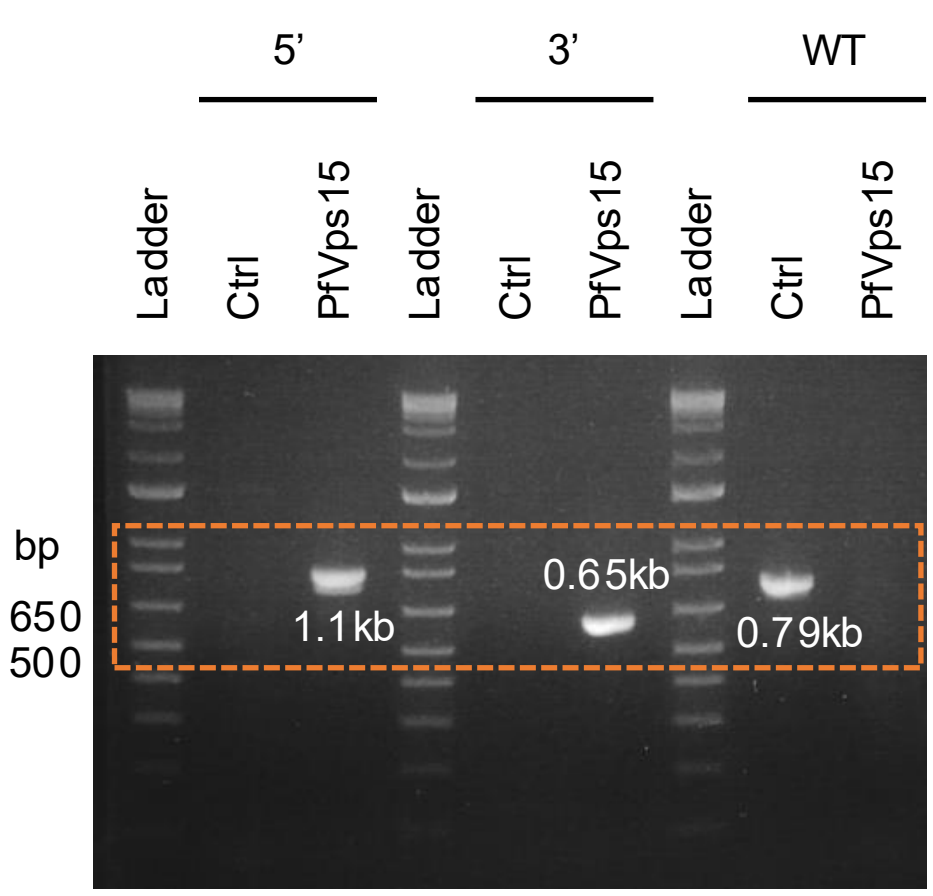

Related to Fig S1B

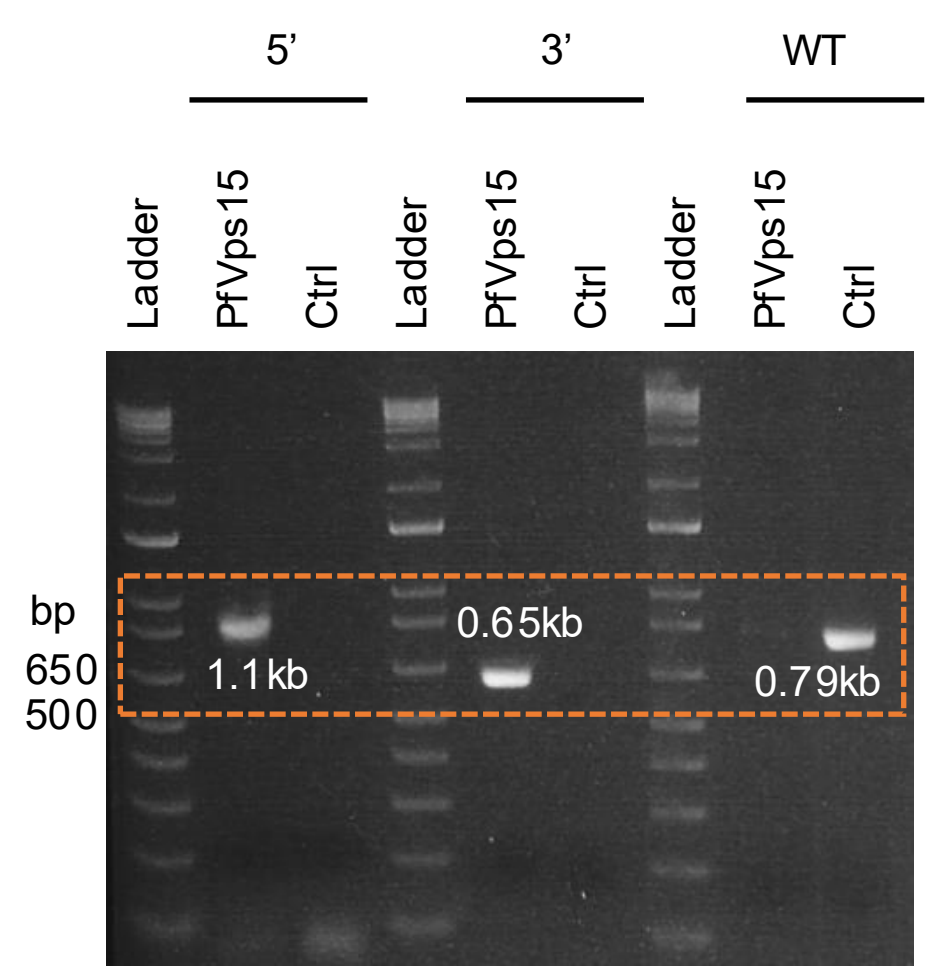

Related to Fig S1F

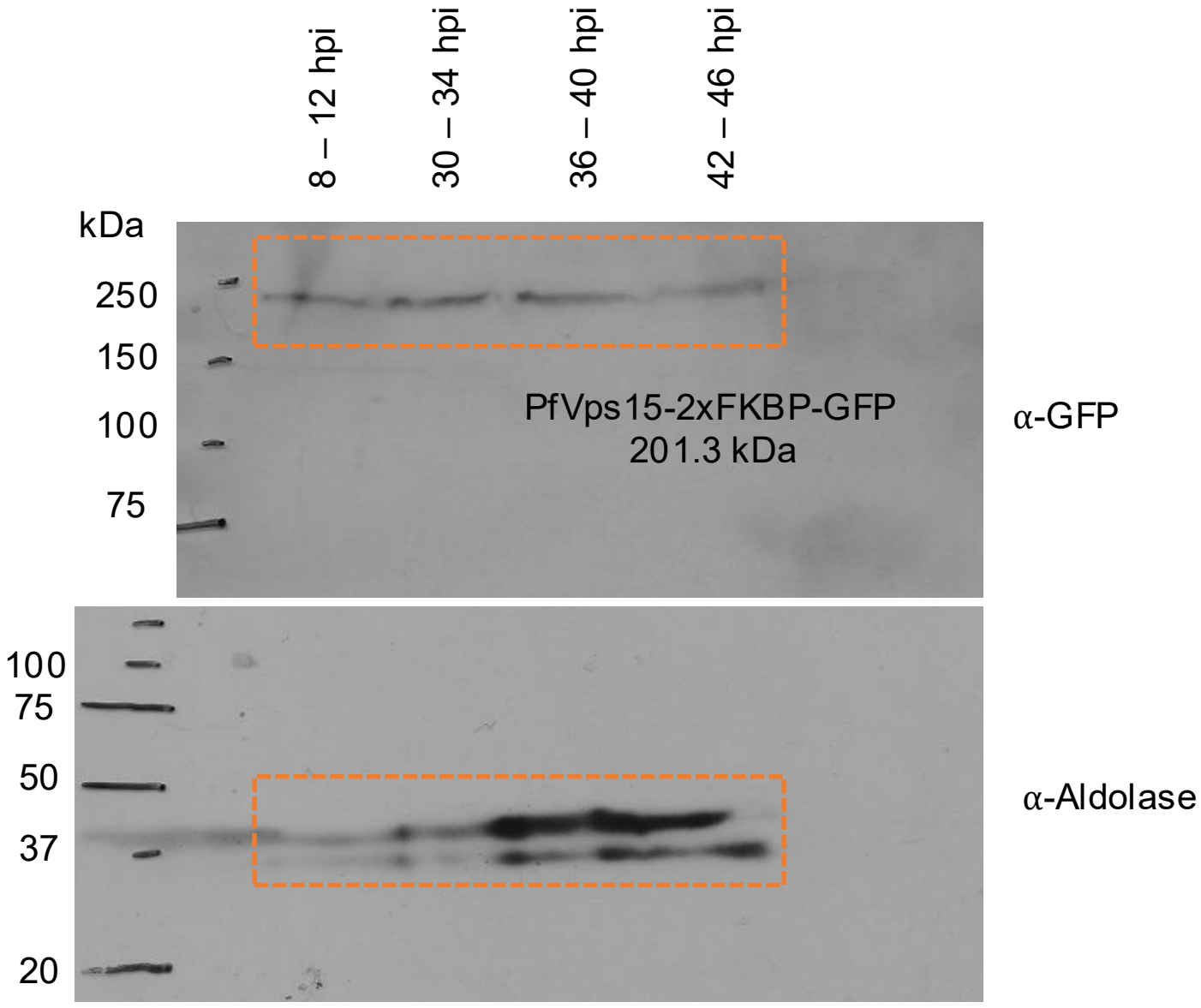

Related to Fig S1C

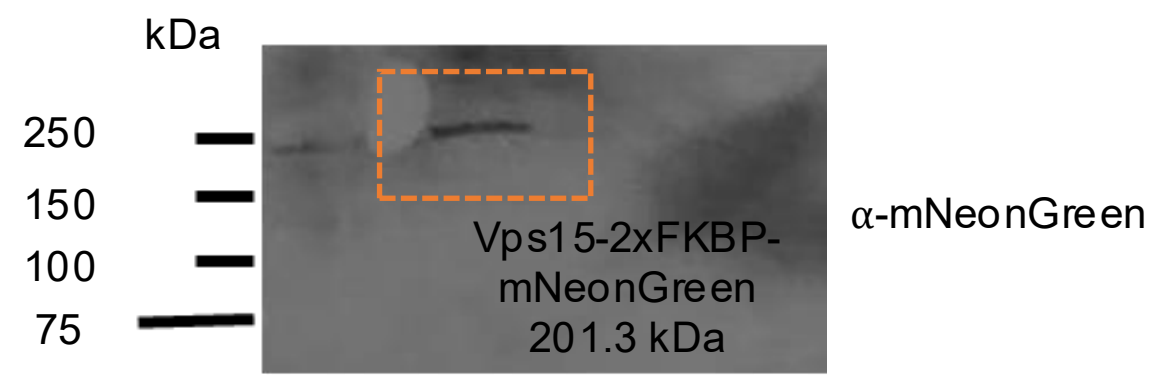

Related to Fig S1G

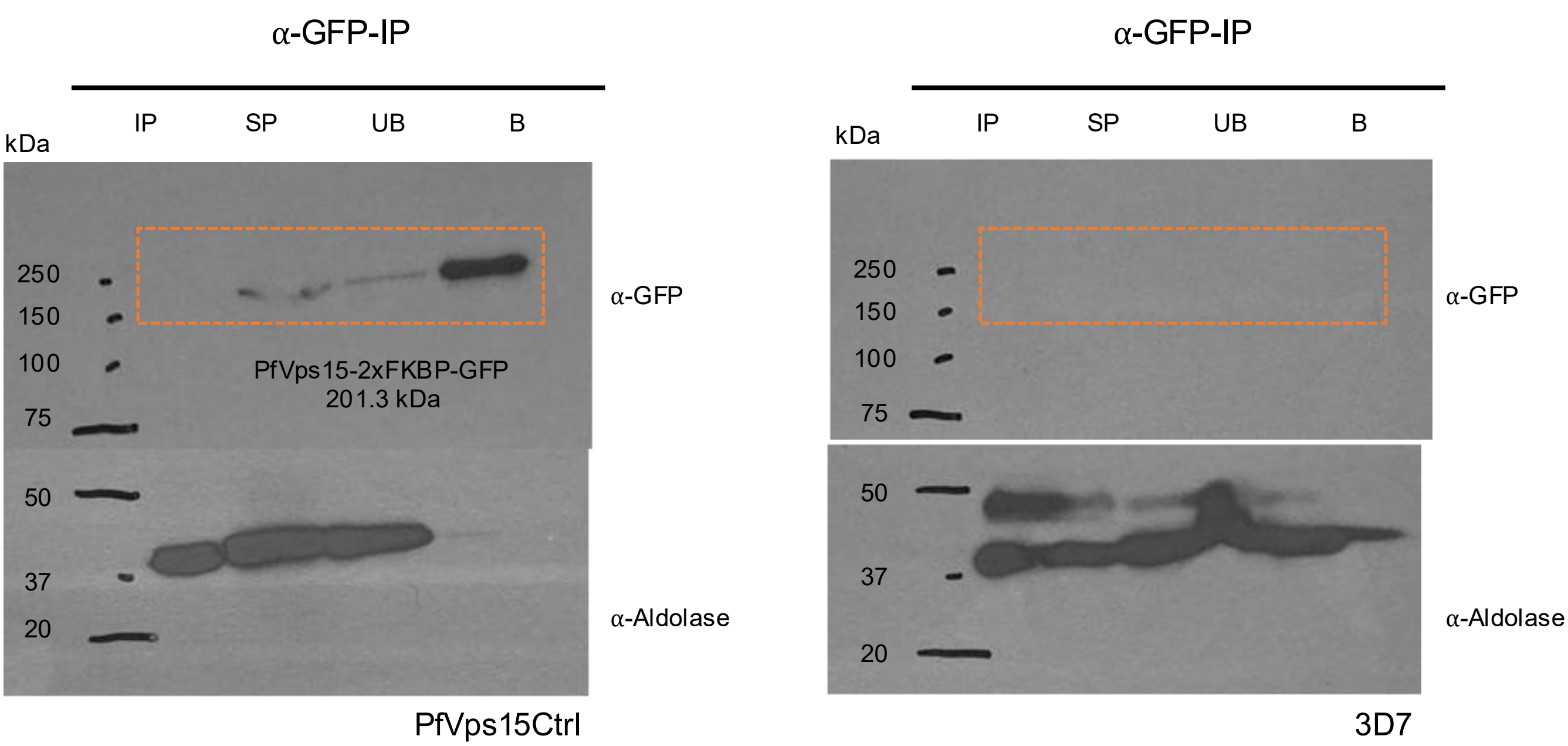

IP: Insoluble proteins
SP: Soluble proteins
UB: Unbound proteins
B: Bead bound proteins

Related to Fig S2
