# Supplementary material for: The malaria parasite Plasmodium falciparum Vps15 is a Phosphatidylinositol 3-kinase interactor important for host-cell cytosol delivery to the food vacuole and apicoplast and mitochondrion dynamics
Source: PLoS Pathog. 2026 Aug 18;22(8):e1014526. doi: 10.1371/journal.ppat.1014526 (PMC13497265; doi:10.1371/journal.ppat.1014526)
Supplement: Supplementary file 7 [file ppat.1014526.s013.pdf]

Response to reviewers

Editor comments:

The reviewers commend the authors for their substantial efforts in revising the manuscript; although the newly added data have strengthened the study, some issues still require amendment, none of which fundamentally undermine the overall story, and which, once addressed, would support publication.

Importantly, even though the phenotypic observations reported here largely mirror those described for the conditional knockout of PI3K (doi.org/10.1038/s41598-025-01397-1), that study appeared while the present revised manuscript was under review and is thus covered by the journal's scooping policy, which should prevail in the evaluation of originality.

We thank the editor and the reviewers for their time spent evaluating our manuscript. As a result of the comments, substantial experimental work has been performed which has broadened the scope of the work and strengthened our conclusions.

We here provide a point-by-point response to each reviewers' comments. Our comments to the reviewers are in green and the ones for the editor are in blue.

Reviewer #1: In this extensively revised version of an earlier manuscript on the function of P. falciparum VPS15 the authors included more co-localization data for VPS15, clarified the growth phenotype and did comparisons of PfVPS15 and VPS15 of other organisms. In addition, in response to other reviewers, work on the impact of VPS15 knock sideways on the apicoplast was included. These experiments show an additional phenotype of the VPS15 KS beside the HCC uptake defect reported in the original submission.
My concern on the growth defect analysis upon VPS15 knock sideways from the original submission was fully addressed and the preliminary glmS parasite line data was removed. Overall the paper has gained in depth and contributes useful information for researchers studying P. falciparum and apicomplexan biology in general.

-We thank the reviewer for their positive assessment.

Reviewer #2: In this manuscript, Bourgeois et al., have characterised Vps15 homologue in P. falciparum. The Vps15 homologue in a closely related organism T. gondii contains the WD40 sequence, and it is shown to interact with PIP3K and Autophagy machinery and plays role in apicoplast maintenance. In the same line, autophagy machinery is shown to associate with the maintenance of the apicoplast, and the PI3K is localised to the food-vacuole and the apicoplast in *P. falciparum*. The Vps15 homologue in P. falciparum does not contain the WD40 sequence; however, the present manuscript shows the interaction of Vps15 and PI3K, whereas other components of the expected protein complex are not identified. The mis-localization strategy (knock sideway) shows that the protein is essential for the parasite; knock sideways causes a reduction in PI3P levels as well as a reduction in uptake of host-cell cytosol (haemoglobin) to the food vacuole.

-We thank the Reviewer for their summary of our work but would like to clarify one important aspect. As mentioned in our initial response to the reviewers' comments, **no direct evidence (i.e.** immunoprecipitation data) **has been published on any TgVps15 interacting protein**. The paper on TgVps15 in Plos Pathogens (PLoS Pathog. 2022;18(11):e1010922.) only shows that TgATG8 redistributes from the apicoplast to the cytosol and TgATG18 from punctae to the cytosol upon TgVps15 KD. The authors state that the localization between TgVps15 and ATG18 was at best observed only occasionally but that under nutrient deprivation in almost 40% of parasites TgVPS15 and TgATG18 co-localized in these puncta. This seemed to have been determined by the presence of yellow (overlap of green and red) in individual cells and not by quantitative analysis. It is standard knowledge that even properly quantified colocalization cannot be used to infer interaction between 2 proteins. These are indirect data, from which functional interconnection can be inferred but interaction is speculative at best. Therefore, it is unknown whether TgVps15 interacts with TgPI3K nor any component of the autophagy machinery.

Reviewer #3: This revised manuscript from Bourgeois et al focuses on the PI3K-interacting protein Vps15 from the malaria parasite Plasmodium falciparum. The authors have substantially improved

the quantity and quality of the data supporting their main claims. First, they show that PfVps15 lacks the WD40 domain found in most orthologs, and that the P. falciparum genome lacks evidence for those interacting partners that are known bind to the WD40 domain in other eukaryotes. Second, they have expanded their localization analysis such that Vps15 has been colocalized with a number of organellar markers and claim "partial" co-localization with endosomal, food vacuole and apicopast markers. Third, they have augmented their study of the effects of PfVps15 mislocalization to provide convincing evidence that one consequence is the disruption of trafficking of endocytic material to the food vacuole and the accumulation of undigested hemoglobin. Fourth, the authors have added an analysis of the effects of PfVps15 mislocalization on the elongation and segregation of the apicoplast during schizogony.

-We thank the reviewer for their assessment.

Part II – Major Issues: Key Experiments Required for Acceptance
Please use this section to detail the key new experiments or modifications of existing experiments that should be absolutely required to validate study conclusions.

Generally, there should be no more than 3 such required experiments or major modifications for a "Major Revision" recommendation. If more than 3 experiments are necessary to validate the study conclusions, then you are encouraged to recommend "Reject".

Reviewer #1:

I have no major criticisms left for the previously included data of the manuscript but some recommendations and questions for the quite substantial newly added data:

Major points
-1. The Rab5a phenotype upon VPS15 KS in Fig. 4C is not convincing. The control in this figure shows fewer Rab5a foci than after VPS15 KS. However, in Fig. 2E (row 1 and 2) which contains parasites of similar age to Fig. 4C without VPS15 KS, the number of dots in the parasites seems

similar to the VPS15 KS in 4C. Based on this the control cell shown in Fig. 4C seems to be an exception, maybe due to more efficient fixing and resulting reduced antibody access in that sample? A proper quantification of the phenotype from 3 independent IFA experiments are needed to reach a conclusion here.

-As requested by the reviewer, we have now quantified the phenotype in two independent experiments. We found that the PfVps15mis line had on average twice as many foci than the control (Ctrl: 3.30 ± 1.59 vs Mis: 6.71 ± 2.54).

-Generally, are the authors sure this anti-Rab5a serum is specific? Can they exclude it crossreacts with Rab5b and Rab5c?

-The antibody was provided by Asif Mohmmed. There is no further validation in their manuscript http://dx.doi.org/10.1016/j.bbamcr.2015.08.007 0167-4889. We therefore cannot ascertain whether it cross reacts. Because of this, we only use the anti-Rab5a serum as a marker for endocytic structures. We do not make any claim as to any function of Rab5a.

2. Fig. 4D: again a quantification would be revealing, extra FV structures containing fluo-dextran were previously also reported in WT cells (Abu Bakar et al DOI: 10.1242/jcs.061499) and only a quantification can show whether this is an effect of VPS15 KS or not.

-As suggested by the reviewer, we have now performed two independent experiments and found that the majority of the control line had 0 or 1 focus whilst the majority of the PfVps15mis line had 2 or more.

Reviewer #2:

-Major concern is that the manuscript does not conclusively show the functional role of Vps15 in the parasite, it shows outcome of the ablation of the target in the parasite.

-This is the same comment as in the previous submission. Again, we apologize but strongly believe that this is an unfair comment. As stated in our initial rebuttal, the most recent studies investigating

proteins involved in HCC-trafficking use the increase in cytosolic vesicles and a reduction in the bloating of the FV in the presence of E64 as standard assays. These studies have been published in high quality journals such as Plos Pathogens and Plos Biology for example. In addition to these 2 assays, we have also shown an increase in Hb foci by IFA, directly demonstrating that some of the accumulated vesicles contain Hb. In addition, we show that PI3P levels are reduced during the same stage of the lifecycle that these assays were performed. To provide more evidence we have now shown that some of the accumulated vesicles are acidified and that some are labelled with PfRab5a, characteristics of endosomal compartments. We have also shown by Western blot that parasite-associated Hb levels are increased in the PfVPS15 KS line and by heme fractionation assays, that the level of cytosolic heme is also increased. In addition, based on reviewer 2's comments, we have now performed the FV bloating assay using shorter incubation times and these clearly show that there is less bloating in the KS line compared to the control. Furthermore, there is no difference in cell size at these time points. We have thus unequivocally demonstrated that PfVps15 is critical for the synthesis of PI3P and for HCC vesicular trafficking. We have also now provided data revealing the PfVps15 is critical for apicoplast biogenesis and mitochondrion fission. In addition, our use of KS allows us to get a phenotype in a short amount of time, reducing the potential for downstream pleiotrophic effects compared to the PI3K inhibitors and the recently published conditional KO of PfPI3K. We therefore apologize but do not understand what the reviewer is asking for.

-Association of Vps15 with apicoplast is not clear, and effect of sideway knock down are to be supported by qPCR-based analysis to get nuclear/organelle genome ratio, while keeping mitochondrial controls.

-We thank the reviewer for this suggestion. We have now performed the requested experiment and show that apicoplast genome replication is much reduced in the PfVps15 mis line. In addition, we now also show that processing of an apicoplast resident protein is reduced, suggesting that protein delivery to the organelle is also compromised.

-In addition, the localisation studies are very poor, the IFA quality is very low, and to better resolve this, the study needs to employ other methods including super-resolution microscopy and expansion microscopy, and, if possible immunoEM, to derive conclusive localization data.

-We apologize but this is an unfair comment. Our images are taken with a Deltavision Elite microscope followed by 3D deconvolution. The resolution is equivalent those obtained with 3D confocal microscopy. Several studies from different labs are still showing images taken with an epifluorescence microscope, with no Z information. For example, this paper published in PLoS Biology https://doi.org/10.1371/journal.pbio.3002639

In addition, we have not found any study in *P. falciparum* looking at endosomal structures using any of the proposed methods (using markers such as Rab5a, b and c or Rab7, Vps45, RbsnL etc…). They certainly have their place when the biological question to answer requires more resolution, but this is not true for the majority of studies.

-Nonetheless, we have now quantified all the localization data and show that the majority of PfVps15 seems to be associated with structures labelled with the endocytic markers PfRab7 and to a lesser extent PfRab5a. This leads us to speculate that perhaps these structures serve as a platform for the generation of PI3P that is then used in processes such HCC trafficking, apicoplast biogenesis and mitochondrion fission. We have now added a cartoon of our model as Fig. 9.

Other points:

-It is not clear why/how the protein shows interaction with PIP3K in the absence of the WD40 sequence region.

-As we wrote in our previous response to reviewers, several studies have shown that **the WD40 domain is not required for interaction with Vps34.** The required domain is the Vps15 pseudokinase domain that interacts directly with the Vps34 catalytic domain. **This pseudokinase domain is present in PfVps15.** In other systems, the WD40 domain is important for binding to Rab5a positive early endosomal vesicles. In addition, the WD40 repeats appear to bridge the Vps38/Atg6 heterodimer to the Vps15-Vps34 Complex II. It is also sufficient to bind to Gpa1 and Atg14.

-Fig 3E and G: the results show accumulation of hemoglobin containing vacuoles, however the hemozoin content remains nearly similar.

-This is explained by the fact that the majority of parasite associated heme is in the form of hemozoin i.e more than 90% whilst cytosolic heme is between 1 and 2%. There is a 1.45-fold increase in cytosolic heme in the KS (1.9% vs 1.31%, so a difference of 0.6%). and a 1.6% decrease in the fraction of heme made up of hemozoin in the KS vs the WT (90.05% vs 91.65%) however it is not statistically significant. Therefore, there is a bigger % of reduction in hemozoin (1.6% reduction vs 0.6% increase in cytosolic Hb) however it is only a small fraction of the total amount of heme present as hemozoin.

-Does knock sideway disrupts protein transport to apicoplast?

-Thank you for the suggestion. We have performed the experiment and show that processing of an apicoplast resident protein is reduced, suggesting that protein delivery to the organelle is also compromised.

-It will be appropriate to show effect on knock down on mitochondria to show specificity of effect on apicoplast.

-Thank you for this suggestion. We have performed the experiment and surprisingly found that although the mitochondrion expands properly, is it not separated into individual merozoites in late schizonts, suggesting a default in fission in the PfVps15 mis line. Whilst this was initially puzzling, a recent paper in *C. elegans* identified a PI3P binding protein essential for mitochondrion fission (https://doi.org/10.1083/jcb.202508040). This provides support that the phenotype we observed is not just a side effect but that PfVps15, through the generation of PI3P, has a role in this process. Interestingly, this protein is required for the creation of membrane contact sites between PI3P-labelled early endosomes and the mitochondrion. This fits with our speculative model that in *P. falciparum* asexual stages, PI3P could be generated through a structure labelled with endocytic markers. Since the biology of endosomes is poorly understood in Pf, we do not feel confident in naming them other than endosome-like structures. In *T. gondii*, a structure named the endosome-

like compartment acts as a sorting station between the Golgi apparatus and different organelles such as those of the apical complex. However, whether the structures labelled with PfRab5a and PfRab7 are equivalent to the *T. gondii* ELCs is currently unknown.

-The authors have determined PI3P levels after ablation of vps15; it will be appropriate to determine the status of PI3K in this situation (levels, localisation, etc.)

-This would be great however as far as we know, no lab has succeeded in tagging PfPI3K. We have tried commercial anti-PI3K mammalian antibodies, and these do not work in our hands. In other systems, Vps15 is required to activate PI3K so the decrease in PI3P we measure could either be from the absence of Vps15 in the PI3K complex or perhaps the translocation of both PI3K and Vps15 to the nucleus. In other systems, PI3K is quite stable in absence of Vps15 though its activity is reduced. Perhaps it is the same in Pf. That being said, whatever happens with PI3K, it does not impact our findings with Vps15. Therefore, we respectfully disagree that our study requires an extensive characterization of PI3K.

Reviewer #3:

Altogether, these findings present a picture of PfVps15 that is consistent with a role in partnering with PI3K, and with the consequence that when PfVps/PI3K is disabled, the loss of PI3P resulting in defects in hemoglobin endocytosis and apicoplast maturation. However, the precise location(s) of PfVps/PI3K, and therefore PI3P synthesis, remains incompletely resolved. The consensus emerging from the PfVps localization studies reported here is that of association with cytosolic puncta that appear, in some cases, to lie proximal to the food vacuole and the apicoplast and, in some cases, to colocalize with endosomal markers. The relationship to pools of PI3P at the food vacuole and the apicoplast membranes remains unclear; does PI3P reach these destinations through lipid/membrane trafficking (this would seem to be the case for the FV, as PI3P would be expected to be generated at endosomal structures)? The lack of a coherent structural model locating PfVps/PI3K in the asexual parasite and relating it to the FV and apicoplast PI3P pools reduces the significance of the manuscript.

-Based on the reviewer's suggestion and additional data we obtained from quantifying all the localization experiments showing that the majority of PfVps15 seems to be associated with structures labelled with the endocytic markers PfRab7 and to a lesser extent PfRab5a, we now provide a speculative model proposing that perhaps these structures serve as a platform for the generation of PI3P that is then used in processes such HCC trafficking, apicoplast biogenesis and mitochondrion fission. As we responded to reviewer 2, we surprisingly found that though the mitochondrion expands properly, is it not separated into individual merozoites in late schizonts, suggesting a default in fission in the PfVps15 mis line. Whilst this was initially puzzling, a recent paper in *C. elegans* identified a PI3P binding protein essential for mitochondrion fission (https://doi.org/10.1083/jcb.202508040). Interestingly, this protein is required for the creation of membrane contact sites between PI3P-labelled early endosomes and the mitochondrion. This fits with our speculative model that in *P. falciparum* asexual stages, PI3P could be generated through a structure labelled with endocytic markers after which it can be used in cellular processes.

Furthermore, the phenotypic observations in this manuscript largely follow those reported for the conditional knockout of PI3K (doi.org/10.1038/s41598-025-01397-1), which is to be expected if they form a functional complex. This mitigates the originality and impact of the findings, and I am not persuaded to alter my original assessment that they do not meet the criterion of "high importance and broad interest to the community".

As written by the editor, that study appeared while our manuscript was under review and is thus covered by the journal's scooping policy, which should prevail in the evaluation of originality. In addition, we now provide new data showing a role of PfVps15, and likely PI3P, in mitochondrial fission. This has only been recently described for the first time in *C. elegans*. In addition, as we wrote above, quantification of the colocalization data has led us to propose a model where PI3P would be generated through a platform labelled with endocytic markers. From this, it would then be made available for the different processes requiring it.

Part III – Minor Issues: Editorial and Data Presentation Modifications
Please use this section for editorial suggestions as well as relatively minor modifications of existing data that would enhance clarity.

Reviewer #1: Minor points:

-We thank reviewer 1 for all their constructive comments.

-The title of the paper does not work that well. Maybe: The malaria parasite Plasmodium falciparum Vps15 is a Phosphatidylinositol 3-kinase interactor important for host-cell cytosol delivery to the food vacuole and apicoplast biogenesis. Or generally reduce detail in the title.

-As suggested by the reviewer we have changed the title:

"The malaria parasite *Plasmodium falciparum* Vps15 is a Phosphatidylinositol 3-kinase interactor important for host-cell cytosol delivery to the food vacuole and apicoplast and mitochondrion dynamics."

- L58: "In its absence, the parasite cannot survive." Specify what is absent, for instance: In absence of VPS15 function, the parasite cannot survive. -Done

- L97: To my knowledge the cited paper does not functionally analyse FCP and its role in HCC transport is not really known. -We agree and have taken it out.

-L139: consider deleting one of the two "constitutively expressed" -Done

- FigS1B: what is the expected size of the PCR fragments? Could this be indicated in the legend?

-Added to the legend as requested.

- FigS1C: which bands of the control correspond to the anti-GFP blot? It seems it contains 5 lanes but the GFP blot contains only 4

-Thank you for noticing this. It was a formatting issue. The first well was actually spill over from the 8-12hpi one. This has now been fixed.

- L171 states that the WD40 domain is present in Tg and this also transpires from Fig. 1, but Table S3 has no plus in that column for Tg. Please check the table for accuracy. -Fixed - The authors could consider making Fig. 1D into Fig. 1B and additionally cite it in line 155. -Done

- L195: not super precise, the foci are not really labelled with PI3P. Maybe "…determine whether the foci overlapped with PI3P containing membranes…"?-Done

-L196, L203, L401: though is colloquial, replace with although. -Done

- Fig2Dii/L204: "However, some PfVps15-labelled structures seemed associated with the elongating apicoplast in developing…" how often was this observed, some can mean many things.

-We have now performed a colocalization analysis with PCCs and also a qualitative characterization of the overlap. This is in Fig. 2G to J.

- L210 and 267: “Important overlap” and “important increase”, replace important with more informative terms. -For 210, we removed important and as we wrote above, we have now performed a colocalization analysis with PCCs and also qualitative characterization of the overlap. This is in Fig. 2 G to J. For 267, we have removed the important and just kept: an increase in ring stage…

- Cite Fig. 3Ci in the results. Also cite Fig. 3Di and in line 284 Fig. 3Cii likely should be Fig. 3Dii. -Fixed

- Fig. 3Ci: are there small hemozoin areas in the +rapa rings? -We do not know, they are certainly not as dark/distinct as the ones in the control. Plz replace the later stage (row 2 of mislocalized) with a better-preserved cell. -Done

- L287: “Perhaps this could be due to increased osmotic fragility? A definitive answer will require further experimental work.” This might be rephrased by removing the question mark and proposing this as one potential of multiple reasons.

-As suggested, we have modified the sentence to:

“One of the potential explanations could be that this is due to increased osmotic fragility.“

- L300 etc: what is lysotracker labelling in these experiments? -Acidic compartments. Can the difference shown in Fig. 4B be quantified in 3 independent experiments with cells of similar stage?

-This has now been done and the data is in Fig. 4Bii. The cell in the mislocalised condition is difficult to gage: if this is the host cell with the parasite, it is too small (compared to the control cell which has the same scale); otherwise it is a free parasite, which should not be scored. I recommend to replace this image with one showing a more representative cell. -Done

- L368 (and other instances, e.g. 409): “In the PfVps15mis line…”: it is not a different parasite line, VPS15 is mislocalised and hence this is only a different condition in the same parasite cell line. While I find it acceptable to not always fully adhere to the strict definition of a cell line, it might still be good to be mindful of the essence of such terms. I would recommend to talking about PfVPS15mis parasites (and PfVPS15ctrl parasites) rather than mis and ctrl lines.

-To take into account potential deleterious effects of Rapa, our control is actually the PfVps15-2xFKBP-GFP without mislocalizer to which we add rapa.This was indicated at line 241 which is now line 285.

-Fig. 6Biii remove one i from elongating. -Done

-L377: replace then with than. -Done

-L378 concludes there is a defect in the biogenesis of the apicoplast in the VPS15KS. I am not sure the experiments indicate a true biogenesis phenotype, maybe more an apicoplast enlargement, division or distribution/inheritance phenotype? -This sentence has been deleted in the new version of the manuscript. In response to other reviewers, we have now added new data showing that the

replication of apicoplast DNA (Fig. 6D) and the processing of an apicoplast-resident protein (Fig. 6E) are reduced which further suggest a default in apicoplast dynamics.

- L393: consider inserting "PI3P-dependent" delivery of proteins… A lack of membrane identity due to PI3P loss would be a good reason for the failure to acquire sufficient material for the apicoplast to grow enough to provide all merozoites with a progeny apicoplast. -Added as suggested.

-L422: why is a Plasmodium-specific adaptation tantalizing? Please explain or rephrase. -We have taken out tantalizing.

-L424: while it is likely that the HCC delivery and apicoplast phenotypes are due to the reduced PI3P levels, this is not directly shown.

-We agree with the reviewer and have changed the sentence to:

"Importantly, conditional inactivation of PfVps15 leads to a reduction in cellular PI3P levels, suggesting that this decrease might be responsible for the defects in the delivery of HCC-containing vesicles to the FV, and apicoplast and mitochondrial dynamics"

- L632: add s to parasite and d to prepare. -Done

Reviewer #2: (No Response)

Reviewer #3:

-1. In the Materials and Methods, there is no description of the method used for quantitation and calculation of enrichment in the IP/mass spec experiment shown in Fig. 1A.

-We thank the reviewer for noticing this We have now added it as requested.

-2. The immunoblot in Fig. 4F lacks markers. The panel labeled “a-Hb” shows three bands, where one is expected. An explanation for this is needed.

-Adult RBCs contain different globin chains (e.g., α and β, plus any minor variants), and many commercial anti-Hb antibodies recognize more than one chain, so you can see several bands corresponding to different globin subunits. This is known and shown/discussed in several papers. Native hemoglobin is a 64kDa tetramer, on a denaturing gel, different forms can be seen. Some examples are below. Several studies only show cropped versions of the blots with only the 12-16 kDa monomer.

-https://doi.org/10.1016/S0166-6851(97)02855-7

-https://doi.org/10.1128/AAC.01478-07
