# Supplementary material for: The malaria parasite Plasmodium falciparum Vps15 is a Phosphatidylinositol 3-kinase interactor important for host-cell cytosol delivery to the food vacuole and apicoplast and mitochondrion dynamics
Source: PLoS Pathog. 2026 Aug 18;22(8):e1014526. doi: 10.1371/journal.ppat.1014526 (PMC13497265; doi:10.1371/journal.ppat.1014526)
Supplement: Supplementary file 8 [file ppat.1014526.s014.pdf]

We thank the reviewers for their comments. We here provide a point-by-point response in green. Text added to the manuscript is in red.

Reviewers' Comments:

Reviewer's Responses to Questions

Part I – Summary
Please use this section to discuss strengths/weaknesses of study, novelty/significance, general execution and scholarship.

Reviewer #1: The authors further improved their manuscript with additional data, including an unexpected mitochondria division phenotype of the VPS15 knock sideways for which a recent paper in C. elegans raised the plausibility. All my concerns had previously been addressed and the revision satisfactorily addresses all reviewer comments.

-Thank you for your support of our work.

Reviewer #2: (No Response)

Part II – Major Issues: Key Experiments Required for Acceptance
Please use this section to detail the key new experiments or modifications of existing experiments that should be absolutely required to validate study conclusions.

Generally, there should be no more than 3 such required experiments or major modifications for a "Major Revision" recommendation. If more than 3 experiments are necessary to validate the study conclusions, then you are encouraged to recommend "Reject".

Reviewer #1: (No Response)

Reviewer #2: (No Response)

Part III – Minor Issues: Editorial and Data Presentation Modifications
Please use this section for editorial suggestions as well as relatively minor modifications of existing data that would enhance clarity.

Reviewer #1: One minor issue is that parts of the manuscript considers PfRab5a as a bona fide endosomal marker. While there is a very differentiated classification of the data on PfRab5a in the results that shows an endosomal function of it is not so clear, the general conclusions assumes it

is a solid endocytosis marker. However, in light of the unclear quality of the Rab5a antibody and the limited evidence that this Rab really has endosomal functions in the parasite, I would not base any conclusion about endosomal characteristics on the Rab5a antibody results. The quantification of co-localisation with VPS15 also shows very low overlap in trophozoites (according to the Pearsons's CE in Fig. 5G). Maybe this is even fitting if PfRab5a is not an endosomal marker while Rab7 could be and shows much better overlap? I recommend caution in any statements regarding Rab5a. Also, in the model in Fig. 9, why is Rab5a in the compartment containing Rab7, these two markers do not co-localize.

-We agree with the reviewer that in absence of a validated function for PfRab5a in endocytosis and the lack of antibody validation, it cannot be considered a validated marker of endosomes/endosome-like compartments. We have therefore removed all the data associated with PfRab5a (original figures 2E, 2G, H and I, 4C and 9). This does not impact our conclusions that a good portion of PfVps15 localizes to endosome-like structures as the colocalization of PfVps15 with episomally expressed PfRab7 was much stronger than with PfRab5a. In addition, the labelling with lysotracker also supports that a portion of the accumulated vesicles have endocytic characteristics.

I would also replace the lysed cell shown for the bloated food vacuole assay (Fig. 5, 2h mislocalized). Generally, the bloating effect was more clearly visible in the cells shown in the previous version of the manuscript. Here the only clearly bloated cell is the 4 h mislocalized (while the controls should be bloated).

-Done, thank you for noticing this.

Finally, I agree with the authors that it is unclear what is to be gained by adding UExM (or other high-end microscopy techniques) for this manuscript. While the cellular context provided by UExM might indeed be valuable, the relevant membranes might not be trivial to detect and likely require optimisation of Bodipy labelling and excellent marker antibodies or smTag marker cell lines for double labelling. Furthermore, UExM is prone to background. Generating meaningful data with additional value for this manuscript would therefore likely require an entire separate study.

-Thank you for your support.

Reviewer #2: The authors have carried out additional experiments and revised the manuscript. Using additional data, they also suggest that the Vps15 knock-sideway also hamper the mitochondrial development, in addition to food-vacuole and apiocplast function. Collectively, these results suggest that the protein a broader role in intracellular vesicular trafficking pathway, Perturbation of this his pathway could consequently impair the organelle development and division. Therefore, the current data do not provide direct evidence supporting a specific role of Vps15 in apicoplast function or mitochondrial fission. The language and terminology used should be toned down in results and discussion to reflect the correlative nature of the observations rather than implying a direct mechanistic role.

-We do not believe that PfVps15 has a broad role in the general secretory pathway since both trophozoite and schizont stage parasites expand in size during KS which demonstrates that expansion of the plasma membrane occurs. The fact that segmented merozoites can be observed in DIC in late schizonts where the KS was induced in early schizonts also supports this. If the secretory pathway would be abrogated, an effect would likely be observed because vesicular transport to the plasma membrane would be disrupted, therefore there would be no expansion of the PM and no segmentation of merozoites. In addition, the advantage of using KS is that it is fast acting and therefore much less prone to pleiotrophic downstream effects.

-We apologize but upon going over the manuscript, we have not found any instances where we have stated that PfVps15 is directly implicated in apicoplast function or mitochondrial fission. At the end of the final paragraph of the Results and Discussion section, starting at line 554, we wrote with regards to PI3P and not PfVps15:

"This therefore suggests that the lipid is likely directly implicated in the delivery of HCC to the FV and in apicoplast and mitochondrion dynamics. Although a role for PI3P in the first two processes correlates with the evidence previously obtained when incubating parasites with PI3K inhibitors[32], when inactivating PI3P binding proteins[31, 33] or conditionally inactivating *PfPI3K*[34], its involvement in *P. falciparum* mitochondrion biology was unknown."

To tone down the statement, we have changed "likely" to potentially:

"the lipid is potentially directly implicated in the delivery of HCC…"

and added potential here:

"its potential involvement in *P. falciparum* mitochondrion biology was unknown."
