# Supplementary material for: The malaria parasite Plasmodium falciparum Vps15 is a Phosphatidylinositol 3-kinase interactor important for host-cell cytosol delivery to the food vacuole and apicoplast and mitochondrion dynamics
Source: PLoS Pathog. 2026 Aug 18;22(8):e1014526. doi: 10.1371/journal.ppat.1014526 (PMC13497265; doi:10.1371/journal.ppat.1014526)
Supplement: Peer review history

# Peer review: Decision Letter 0

**DOI:** 10.1371/journal.ppat.1014526.r001

**Authors:** Dominique Soldati-Favre

26 Jan 2026

PPATHOGENS-D-25-03252

The malaria parasite Plasmodium falciparum Vps15 interacts with Phosphatidylinositol 3-kinase, is critical for host-cell cytosol delivery to the food vacuole and apicoplast biogenesis.

PLOS Pathogens

Dear Dr. Richard,

Thank you for submitting your manuscript to PLOS Pathogens. After careful consideration, we feel that it has merit but does not fully meet PLOS Pathogens's publication criteria as it currently stands. Therefore, we invite you to submit a revised version of the manuscript that addresses the points raised during the review process.

Please submit your revised manuscript by Mar 27 2026 11:59PM. If you will need more time than this to complete your revisions, please reply to this message or contact the journal office at plospathogens@plos.org. When you're ready to submit your revision, log on to https://www.editorialmanager.com/ppathogens/ and select the 'Submissions Needing Revision' folder to locate your manuscript file.

Please include the following items when submitting your revised manuscript:

* A letter that responds to each point raised by the editor and reviewer(s). You should upload this letter as a separate file labeled 'Response to Reviewers'. This file does not need to include responses to any formatting updates and technical items listed in the 'Journal Requirements' section below.

* A marked-up copy of your manuscript that highlights changes made to the original version. You should upload this as a separate file labeled 'Revised Manuscript with Track Changes'.

* An unmarked version of your revised paper without tracked changes. You should upload this as a separate file labeled 'Manuscript'.

If you would like to make changes to your financial disclosure, competing interests statement, or data availability statement, please make these updates within the submission form at the time of resubmission. Guidelines for resubmitting your figure files are available below the reviewer comments at the end of this letter.

We look forward to receiving your revised manuscript.

Kind regards,

Dominique Soldati-Favre

Section Editor

PLOS Pathogens

Sumita Bhaduri-McIntosh

Editor-in-Chief

PLOS Pathogens

orcid.org/0000-0003-2946-9497

Michael Malim

Editor-in-Chief

PLOS Pathogens

orcid.org/0000-0002-7699-2064

**Additional Editor Comments:**

The reviewers commend the authors for their substantial efforts in revising the manuscript; although the newly added data have strengthened the study, some issues still require amendment, none of which fundamentally undermine the overall story, and which, once addressed, would support publication.

Importantly, even though the phenotypic observations reported here largely mirror those described for the conditional knockout of PI3K (doi.org/10.1038/s41598-025-01397-1), that study appeared while the present revised manuscript was under review and is thus covered by the journal’s scooping policy, which should prevail in the evaluation of originality.

Since *PLOS Pathogens*  typically does not provide a second major revision, the authors are encouraged to resolve all remaining points in this round.

**Journal Requirements:**

If the reviewer comments include a recommendation to cite specific previously published works, please review and evaluate these publications to determine whether they are relevant and should be cited. There is no requirement to cite these works unless the editor has indicated otherwise.

1) We ask that a manuscript source file is provided at Revision. Please upload your manuscript file as a .doc, .docx, .rtf or .tex. If you are providing a .tex file, please upload it under the item type u2018LaTeX Source Fileu2019 and leave your .pdf version as the item type u2018Manuscriptu2019.

2) Please upload all main figures as separate Figure files in .tif or .eps format. For more information about how to convert and format your figure files please see our guidelines:

https://journals.plos.org/plospathogens/s/figures

3) We notice that your supplementary Figures are included in the manuscript file. Please remove them and upload them with the file type 'Supporting Information'. Please ensure that each Supporting Information file has a legend listed in the manuscript after the references list.

4) Please amend your detailed Financial Disclosure statement. This is published with the article. It must therefore be completed in full sentences and contain the exact wording you wish to be published.

1) State what role the funders took in the study. If the funders had no role in your study, please state: "The funders had no role in study design, data collection and analysis, decision to publish, or preparation of the manuscript."

2) If any authors received a salary from any of your funders, please state which authors and which funders..

If you did not receive any funding for this study, please simply state: u201cThe authors received no specific funding for this work.u201d

**Reviewers' Comments:**

Reviewer's Responses to Questions

**Part I - Summary**

Please use this section to discuss strengths/weaknesses of study, novelty/significance, general execution and scholarship.

Reviewer #1: In this extensively revised version of an earlier manuscript on the function of P. falciparum VPS15 the authors included more co-localization data for VPS15, clarified the growth phenotype and did comparisons of PfVPS15 and VPS15 of other organisms. In addition, in response to other reviewers, work on the impact of VPS15 knock sideways on the apicoplast was included. These experiments show an additional phenotype of the VPS15 KS beside the HCC uptake defect reported in the original submission.

My concern on the growth defect analysis upon VPS15 knock sideways from the original submission was fully addressed and the preliminary glmS parasite line data was removed. Overall the paper has gained in depth and contributes useful information for researchers studying P. falciparum and apicomplexan biology in general.

Reviewer #2: In this manuscript, Beourgeois et al., have characterised Vps15 homologue in P. falciparum. The Vps15 homologue in a closely related organism T. gondii contains the WD40 sequence, and it is shown to interact with PIP3K and Autophagy machinery, and plays role in apicoplast maintenance. In the same line, autophagy machinery is shown to associate with the maintenance of the apicoplast, and the PI3K is localised to the food-vacuole and the apicoplast in P. falciparum. The Vps15 homologue in P. falciparum does not contain the WD40 sequence; however, the present manuscript shows the interaction of Vps15 and PI3K, whereas other components of the expected protein complex are not identified.

The mis-localization strategy (knock sideway) shows that the protein is essential for the parasite; knock sideways causes a reduction in PI3P levels as well as a reduction in uptake of host-cell cytosol (haemoglobin) to the food vacuole.

Reviewer #3: This revised manuscript from Bourgeouis et al focuses on the PI3K-interacting protein Vps15 from the malaria parasite Plasmodium falciparum. The authors have substantially improved the quantity and quality of the data supporting their main claims. First, they show that PfVps15 lacks the WD40 domain found in most orthologs, and that the P. falciparum genome lacks evidence for those interacting partners that are known bind to the WD40 domain in other eukaryotes. Second, they have expanded their localization analysis such that Vps15 has been colocalized with a number of organellar markers and claim “partial” co-localization with endosomal, food vacuole and apicopast markers. Third, they have augmented their study of the effects of PfVps15 mislocalization to provide convincing evidence that one consequence is the disruption of trafficking of endocytic material to the food vacuole and the accumulation of undigested hemoglobin. Fourth, the authors have added an analysis of the effects of PfVps15 mislocalization on the elongation and segregation of the apicoplast during schizogony.

**********

**Part II – Major Issues: Key Experiments Required for Acceptance**

Please use this section to detail the key new experiments or modifications of existing experiments that should be absolutely required to validate study conclusions.

Generally, there should be no more than 3 such required experiments or major modifications for a "Major Revision" recommendation. If more than 3 experiments are necessary to validate the study conclusions, then you are encouraged to recommend "Reject".

Reviewer #1: I have no major criticisms left for the previously included data of the manuscript but some recommendations and questions for the quite substantial newly added data:

Major points

1. the Rab5a phenotype upon VPS15 KS in Fig. 4C is not convincing. The control in this figure shows fewer Rab5a foci than after VPS15 KS. However, in Fig. 2E (row 1 and 2) which contains parasites of similar age to Fig. 4C without VPS15 KS, the number of dots in the parasites seems similar to the VPS15 KS in 4C. Based on this the control cell shown in Fig. 4C seems to be an exception, maybe due to more efficient fixing and resulting reduced antibody access in that sample? A proper quantification of the phenotype from 3 independent IFA experiments are needed to reach a conclusion here.

Generally, are the authors sure this anti-Rab5a serum is specific? Can they exclude it crossreacts with Rab5b and Rab5c?

2. Fig. 4D: again a quantification would be revealing, extra FV structures containing fluo-dextran were previously also reported in WT cells (Abu Bakar et al DOI: 10.1242/jcs.061499) and only a quantification can show whether this is an effect of VPS15 KS or not.

Reviewer #2: Major concern is that the manuscript does not conclusively show the functional role of Vps15 in the parasite, it shows outcome of the ablation of the target in the parasite. Association of Vps15 with apicoplast is not clear, and effect of sideway knock down are to be supported by qPCR-based analysis to get nuclear/organelle genome ratio, while keeping mitochondrial controls. In addition, the localisation studies are very poor, the IFA quality is very low, and to better resolve this, the study needs to employ other methods including super-resolution microscopy and expansion microscopy, and, if possible immunoEM, to derive conclusive localization data.

Other points:

It is not clear why/how the protein shows interaction with PIP3K in the absence of the WD40 sequence region.

Fig 3E and G: the results show accumulation of hemoglobin containing vacuoles, however the hemozoin content remains nearly similar.

Does knock sideway disrupts protein transport to apicoplst? It will be appropriate to show effect on knock down on mitochondria to show specificity of effect on apicoplast.

The authors have determined PI3P levels after ablation of vps15; it will be appropriate to determine the status of PI3K in this situation (levels, localisation, etc.)

Reviewer #3: Altogether, these findings present a picture of PfVps15 that is consistent with a role in partnering with PI3K, and with the consequence that when PfVps/PI3K is disabled, the loss of PI3P resulting in defects in hemoglobin endocytosis and apicoplast maturation. However, the precise location(s) of PfVps/PI3K, and therefore PI3P synthesis, remains incompletely resolved. The consensus emerging from the PfVps localization studies reported here is that of association with cytosolic puncta that appear, in some cases, to lie proximal to the food vacuole and the apicoplast and, in some cases, to colocalize with endosomal markers. The relationship to pools of PI3P at the food vacuole and the apicoplast membranes remains unclear; does PI3P reach these destinations through lipid/membrane trafficking (this would seem to be the case for the FV, as PI3P would be expected to be generated at endosomal structures)? The lack of a coherent structural model locating PfVps/PI3K in the asexual parasite and relating it to the FV and apicoplast PI3P pools reduces the significance of the manuscript. Furthermore, the phenotypic observations in this manuscript largely follow those reported for the conditional knockout of PI3K (doi.org/10.1038/s41598-025-01397-1), which is to be expected if they form a functional complex. This mitigates the originality and impact of the findings, and I am not persuaded to alter my original assessment that they do not meet the criterion of “high importance and broad interest to the community”.

**********

**Part III – Minor Issues: Editorial and Data Presentation Modifications**

Please use this section for editorial suggestions as well as relatively minor modifications of existing data that would enhance clarity.

Reviewer #1: Minor points:

- The title of the paper does not work that well. Maybe: The malaria parasite Plasmodium falciparum Vps15 is a Phosphatidylinositol 3-kinase interactor important for host-cell cytosol delivery to the food vacuole and apicoplast biogenesis. Or generally reduce detail in the title.

- L58: “In its absence, the parasite cannot survive.” Specify what is absent, for instance: In absence of VPS15 function, the parasite cannot survive.

- L97: To my knowledge the cited paper does not functionally analyse FCP and its role in HCC transport is not really known.

- L139: consider deleting one of the two “constitutively expressed”

- FigS1B: what is the expected size of the PCR fragments? Could this be indicated in the legend?

- FigS1C: which bands of the control correspond to the anti-GFP blot? It seems it contains 5 lanes but the GFP blot contains only 4.

- L171 states that the WD40 domain is present in Tg and this also transpires from Fig. 1, but Table S3 has no plus in that column for Tg. Please check the table for accuracy.

- The authors could consider making Fig. 1D into Fig. 1B and additionally cite it in line 155.

- L195: not super precise, the foci are not really labelled with PI3P. Maybe “…determine whether the foci overlapped with PI3P containing membranes…”?

- L196, L203, L401: though is colloquial, replace with although

- Fig2Dii/L204: “However, some PfVps15-labelled structures seemed associated with the elongating apicoplast in developing…” how often was this observed, some can mean many things.

- L210 and 267: “Important overlap” and “important increase”, replace important with more informative terms.

- Cite Fig. 3Ci in the results. Also cite Fig. 3Di and in line 284 Fig. 3Cii likely should be Fig. 3Dii.

- Fig. 3Ci: are there small hemozoin areas in the +rapa rings? Plz replace the later stage (row 2 of mislocalized) with a better-preserved cell.

- L287: “Perhaps this could be due to increased osmotic fragility? A definitive answer will require further experimental work.” This might be rephrased by removing the question mark and proposing this as one potential of multiple reasons.

- L300 etc: what is lysotracker labelling in these experiments? Can the difference shown in Fig. 4B be quantified in 3 independent experiments with cells of similar stage? The cell in the mislocalised condition is difficult to gage: if this is the host cell with the parasite, it is too small (compared to the control cell which has the same scale); otherwise it is a free parasite, which should not be scored. I recommend to replace this image with one showing a more representative cell.

- L368 (and other instances, e.g. 409): “In the PfVps15mis line…”: it is not a different parasite line, VPS15 is mislocalised and hence this is only a different condition in the same parasite cell line. While I find it acceptable to not always fully adhere to the strict definition of a cell line, it might still be good to be mindful of the essence of such terms. I would recommend to talking about PfVPS15mis parasites (and PfVPS15ctrl parasites) rather than mis and ctrl lines.

- Fig. 6Biii remove one i from elongating.

- L377: replace then with than

- L378 concludes there is a defect in the biogenesis of the apicoplast in the VPS15KS. I am not sure the experiments indicate a true biogenesis phenotype, maybe more an apicoplast enlargement, division or distribution/inheritance phenotype?

- L393: consider inserting “PI3P-dependent” delivery of proteins… A lack of membrane identity due to PI3P loss would be a good reason for the failure to acquire sufficient material for the apicoplast to grow enough to provide all merozoites with a progeny apicoplast.

- L422: why is a Plasmodium-specific adaptation tantalizing? Please explain or rephrase.

- L424: while it is likely that the HCC delivery and apicoplast phenotypes are due to the reduced PI3P levels, this is not directly shown

- L632: add s to parasite and d to prepare

Reviewer #2: (No Response)

Reviewer #3: 1.In the Materials and Methods, there is no description of the method used for quantitation and calculation of enrichment in the IP/mass spec experiment shown in Fig. 1A.

2. The immunoblot in Fig. 4F lacks markers. The panel labeled “a-Hb” shows three bands, where one is expected. An explanation for this is needed.

**********

PLOS authors have the option to publish the peer review history of their article (what does this mean?). If published, this will include your full peer review and any attached files.

If you choose “no”, your identity will remain anonymous but your review may still be made public.

**Do you want your identity to be public for this peer review?**  For information about this choice, including consent withdrawal, please see our Privacy Policy.

Reviewer #1: No

Reviewer #2: No

Reviewer #3: No

**Figure resubmission:** While revising your submission, we strongly recommend that you use PLOS’s NAAS tool (https://ngplosjournals.pagemajik.ai/artanalysis) to test your figure files. NAAS can convert your figure files to the TIFF file type and meet basic requirements (such as print size, resolution), or provide you with a report on issues that do not meet our requirements and that NAAS cannot fix.

After uploading your figures to PLOS’s NAAS tool - https://ngplosjournals.pagemajik.ai/artanalysis, NAAS will process the files provided and display the results in the "Uploaded Files" section of the page as the processing is complete. If the uploaded figures meet our requirements (or NAAS is able to fix the files to meet our requirements), the figure will be marked as "fixed" above. If NAAS is unable to fix the files, a red "failed" label will appear above. When NAAS has confirmed that the figure files meet our requirements, please download the file via the download option, and include these NAAS processed figure files when submitting your revised manuscript.

**Reproducibility:**

To enhance the reproducibility of your results, we recommend that authors of applicable studies deposit laboratory protocols in protocols.io, where a protocol can be assigned its own identifier (DOI) such that it can be cited independently in the future. Additionally, PLOS ONE offers an option to publish peer-reviewed clinical study protocols. Read more information on sharing protocols at https://plos.org/protocols?utm_medium=editorial-email&utm_source=authorletters&utm_campaign=protocols

# Author comment: Author response to Decision Letter 1

**DOI:** 10.1371/journal.ppat.1014526.r002

7 Jul 2026

Attachment Submitted filename: Response to reviewers.pdf

# Peer review: Decision Letter 1

**DOI:** 10.1371/journal.ppat.1014526.r003

**Authors:** Dominique Soldati-Favre

2 Aug 2026

PPATHOGENS-D-25-03252R1

The malaria parasite Plasmodium falciparum Vps15 is a Phosphatidylinositol 3-kinase interactor important for host-

cell cytosol delivery to the food vacuole and apicoplast and mitochondrion dynamics.

PLOS Pathogens

Dear Dr. Richard,

Thank you for submitting your manuscript to PLOS Pathogens. After careful consideration, we feel that it has merit but does not fully meet PLOS Pathogens's publication criteria as it currently stands. Therefore, we invite you to submit a revised version of the manuscript that addresses the remaining points raised during the review process.

Please submit your revised manuscript by Oct 01 2026 11:59PM. If you will need more time than this to complete your revisions, please reply to this message or contact the journal office at plospathogens@plos.org. When you're ready to submit your revision, log on to https://www.editorialmanager.com/ppathogens/ and select the 'Submissions Needing Revision' folder to locate your manuscript file.

Please include the following items when submitting your revised manuscript:

* A letter that responds to each point raised by the editor and reviewer(s). You should upload this letter as a separate file labeled 'Response to Reviewers'. This file does not need to include responses to any formatting updates and technical items listed in the 'Journal Requirements' section below.

* A marked-up copy of your manuscript that highlights changes made to the original version. You should upload this as a separate file labeled 'Revised Manuscript with Track Changes'.

* An unmarked version of your revised paper without tracked changes. You should upload this as a separate file labeled 'Manuscript'.

If you would like to make changes to your financial disclosure, competing interests statement, or data availability statement, please make these updates within the submission form at the time of resubmission. Guidelines for resubmitting your figure files are available below the reviewer comments at the end of this letter.

As the corresponding author, your ORCID iD is verified in the submission system and will appear in the published article. PLOS supports the use of ORCID, and we encourage all coauthors to register for an ORCID iD and use it as well. Please encourage your coauthors to verify their ORCID iD within the submission system before final acceptance, as unverified ORCID iDs will not appear in the published article. Only the individual author can complete the verification step; PLOS staff cannot verify ORCID iDs on behalf of authors.

We look forward to receiving your revised manuscript.

Kind regards,

Dominique Soldati-Favre

Section Editor

PLOS Pathogens

Sumita Bhaduri-McIntosh

Editor-in-Chief

PLOS Pathogens

orcid.org/0000-0003-2946-9497

Michael Malim

Editor-in-Chief

PLOS Pathogens

orcid.org/0000-0002-7699-2064

**Additional Editor Comments (if provided):**

**Journal Requirements:**

If the reviewer comments include a recommendation to cite specific previously published works, please review and evaluate these publications to determine whether they are relevant and should be cited. There is no requirement to cite these works unless the editor has indicated otherwise.

[Note: HTML markup is below. Please do not edit.]

**Reviewers' Comments:**

Reviewer's Responses to Questions

**Part I - Summary**

Please use this section to discuss strengths/weaknesses of study, novelty/significance, general execution and scholarship.

Reviewer #1: The authors further improved their manuscript with additional data, including an unexpected mitochondria division phenotype of the VPS15 knock sideways for which a recent paper in C. elegans raised the plausibility. All my concerns had previously been addressed and the revision satisfactorily addresses all reviewer comments.

Reviewer #2: (No Response)

**********

**Part II – Major Issues: Key Experiments Required for Acceptance**

Please use this section to detail the key new experiments or modifications of existing experiments that should be absolutely required to validate study conclusions.

Generally, there should be no more than 3 such required experiments or major modifications for a "Major Revision" recommendation. If more than 3 experiments are necessary to validate the study conclusions, then you are encouraged to recommend "Reject".

Reviewer #1: (No Response)

Reviewer #2: (No Response)

**********

**Part III – Minor Issues: Editorial and Data Presentation Modifications**

Please use this section for editorial suggestions as well as relatively minor modifications of existing data that would enhance clarity.

Reviewer #1: One minor issue is that parts of the manuscript considers PfRab5a as a bona fide endosomal marker. While there is a very differentiated classification of the data on PfRab5a in the results that shows an endosomal function of it is not so clear, the general conclusions assumes it is a solid endocytosis marker. However, in light of the unclear quality of the Rab5a antibody and the limited evidence that this Rab really has endosomal functions in the parasite, I would not base any conclusion about endosomal characteristics on the Rab5a antibody results. The quantification of co-localisation with VPS15 also shows very low overlap in trophozoites (according to the Pearsons’s CE in Fig. 5G). Maybe this is even fitting if PfRab5a is not an endosomal marker while Rab7 could be and shows much better overlap? I recommend caution in any statements regarding Rab5a. Also, in the model in Fig. 9, why is Rab5a in the compartment containing Rab7, these two markers do not co-localize.

I would also replace the lysed cell shown for the bloated food vacuole assay (Fig. 5, 2h mislocalized). Generally, the bloating effect was more clearly visible in the cells shown in the previous version of the manuscript. Here the only clearly bloated cell is the 4 h mislocalized (while the controls should be bloated).

Finally, I agree with the authors that it is unclear what is to be gained by adding UExM (or other high-end microscopy techniques) for this manuscript. While the cellular context provided by UExM might indeed be valuable, the relevant membranes might not be trivial to detect and likely require optimisation of Bodipy labelling and excellent marker antibodies or smTag marker cell lines for double labelling. Furthermore, UExM is prone to background. Generating meaningful data with additional value for this manuscript would therefore likely require an entire separate study.

Reviewer #2: The authors have carried out additional experiments and revised the manuscript.

Using additional data, they also suggest that the Vps15 knock-sideway also hamper the mitochondrial development, in addition to food-vacuole and apiocplast function. Collectively, these results suggest that the protein a broader role in intracellular vesicular trafficking pathway, Perturbation of this his pathway could consequently impair the organelle development and division. Therefore, the current data do not provide direct evidence supporting a specific role of Vps15 in apicoplast function or mitochondrial fission. The language and terminology used should be toned down in results and discussion to reflect the correlative nature of the observations rather than implying a direct mechanistic role.

**********

PLOS authors have the option to publish the peer review history of their article (what does this mean?). If published, this will include your full peer review and any attached files.

If you choose “no”, your identity will remain anonymous but your review may still be made public.

**Do you want your identity to be public for this peer review?**  For information about this choice, including consent withdrawal, please see our Privacy Policy.

Reviewer #1: No

Reviewer #2: No

[NOTE: If reviewer comments were submitted as an attachment file, they will be attached to this email and accessible via the submission site. Please log into your account, locate the manuscript record, and check for the action link "View Attachments". If this link does not appear, there are no attachment files.]

**Figure resubmission:**

While revising your submission, we strongly recommend that you use PLOS’s NAAS tool (https://ngplosjournals.pagemajik.ai/artanalysis) to test your figure files. NAAS can convert your figure files to the TIFF file type and meet basic requirements (such as print size, resolution), or provide you with a report on issues that do not meet our requirements and that NAAS cannot fix.

After uploading your figures to PLOS’s NAAS tool - https://ngplosjournals.pagemajik.ai/artanalysis, NAAS will process the files provided and display the results in the "Uploaded Files" section of the page as the processing is complete. If the uploaded figures meet our requirements (or NAAS is able to fix the files to meet our requirements), the figure will be marked as "fixed" above. If NAAS is unable to fix the files, a red "failed" label will appear above. When NAAS has confirmed that the figure files meet our requirements, please download the file via the download option, and include these NAAS processed figure files when submitting your revised manuscript.

**Reproducibility:**

To enhance the reproducibility of your results, we recommend that authors of applicable studies deposit laboratory protocols in protocols.io, where a protocol can be assigned its own identifier (DOI) such that it can be cited independently in the future. Additionally, PLOS ONE offers an option to publish peer-reviewed clinical study protocols. Read more information on sharing protocols at https://plos.org/protocols?utm_medium=editorial-email&utm_source=authorletters&utm_campaign=protocols

# Author comment: Author response to Decision Letter 2

**DOI:** 10.1371/journal.ppat.1014526.r004

4 Aug 2026

Attachment Submitted filename: Response_to_Reviewers_auresp_2.pdf

# Editor report: Decision Letter 2

**DOI:** 10.1371/journal.ppat.1014526.r005

**Authors:** Dominique Soldati-Favre

5 Aug 2026

Dear Dr Richard,

We are pleased to inform you that your manuscript 'The malaria parasite Plasmodium falciparum Vps15 is a Phosphatidylinositol 3-kinase interactor important for host-

cell cytosol delivery to the food vacuole and apicoplast and mitochondrion dynamics.' has been provisionally accepted for publication in PLOS Pathogens.

Before your manuscript can be formally accepted you will need to complete some formatting changes, which you will receive in a follow up email. A member of our team will be in touch with a set of requests.

Please note that your manuscript will not be scheduled for publication until you have made the required changes, so a swift response is appreciated.

IMPORTANT: The editorial review process is now complete. PLOS will only permit corrections to spelling, formatting or significant scientific errors from this point onwards. Requests for major changes, or any which affect the scientific understanding of your work, will cause delays to the publication date of your manuscript.

Should you, your institution's press office or the journal office choose to press release your paper, you will automatically be opted out of early publication. We ask that you notify us now if you or your institution is planning to press release the article. All press must be co-ordinated with PLOS.

Thank you again for supporting Open Access publishing; we are looking forward to publishing your work in PLOS Pathogens.

Best regards,

Dominique Soldati-Favre

Section Editor

PLOS Pathogens

Sumita Bhaduri-McIntosh

Editor-in-Chief

PLOS Pathogens

orcid.org/0000-0003-2946-9497

Michael Malim

Editor-in-Chief

PLOS Pathogens

orcid.org/0000-0002-7699-2064

***********************************************************

Reviewer Comments (if any, and for reference):

# Editor report: Acceptance letter

**DOI:** 10.1371/journal.ppat.1014526.r006

**Authors:** Dominique Soldati-Favre

Dear Dr Richard,

We are delighted to inform you that your manuscript, "The malaria parasite Plasmodium falciparum Vps15 is a Phosphatidylinositol 3-kinase interactor important for host-

cell cytosol delivery to the food vacuole and apicoplast and mitochondrion dynamics.," has been formally accepted for publication in PLOS Pathogens.

We have now passed your article onto the PLOS Production Department who will complete the rest of the pre-publication process. All authors will receive a confirmation email upon publication.

The corresponding author will soon be receiving a typeset proof for review, to ensure errors have not been introduced during production. Please review the PDF proof of your manuscript carefully, as this is the last chance to correct any scientific or type-setting errors. Please note that major changes, or those which affect the scientific understanding of the work, will likely cause delays to the publication date of your manuscript. Note: Proofs for Front Matter articles (Pearls, Reviews, Opinions, etc...) are generated on a different schedule and may not be made available as quickly.

Soon after your final files are uploaded, the early version of your manuscript, if you opted to have an early version of your article, will be published online. The date of the early version will be your article's publication date. The final article will be published to the same URL, and all versions of the paper will be accessible to readers.

For Research Articles, you will receive an invoice from PLOS for your publication fee after your manuscript has reached the completed accept phase. If you receive an email requesting payment before acceptance or for any other service, this may be a phishing scheme. Learn how to identify phishing emails and protect your accounts at https://explore.plos.org/phishing.

Thank you again for supporting open-access publishing; we are looking forward to publishing your work in PLOS Pathogens.

Best regards,

Sumita Bhaduri-McIntosh

Editor-in-Chief

PLOS Pathogens

orcid.org/0000-0003-2946-9497

Michael Malim

Editor-in-Chief

PLOS Pathogens

orcid.org/0000-0002-7699-2064
